# Tegileridine for moderate-to-severe acute pain following abdominal surgery: A randomized, double-blind, phase 3 clinical trial

**DOI:** 10.1016/j.xcrm.2025.102477

**Published:** 2025-12-08

**Authors:** Tingting Wang, Yafeng Wang, Haihui Xie, Zhilin Wu, Shuchun Yu, Yangwen Ou, Mingjun Xu, Wanwei Jiang, Liang Ge, Ju Gao, Qiang Wang, Hexin Gao, Yanjuan Huang, Ping Zhao, Yonghao Yu, He Huang, Jinghua Ren, Zhengyuan Xia, Jiaqiang Zhang, Jianbo Yu, Xiangdong Chen

**Affiliations:** 1 Department of Anesthesiology, Union Hospital, Tongji Medical College, Huazhong University of Science and Technology, Wuhan 430022, China; 2 Institute of Anesthesia and Critical Care Medicine, Union Hospital, Tongji Medical College, Huazhong University of Science and Technology, Wuhan 430022, China; 3 Key Laboratory of Anesthesiology and Resuscitation (Huazhong University of Science and Technology), Ministry of Education, Wuhan, China; 4 Department of Anesthesiology, Affiliated Dongguan People’s Hospital of Southern Medical University, Dongguan, Guangdong 523018, China; 5 Department of Anesthesiology, The Second Affiliated Hospital of Nanchang University, Nanchang 330000, China; 6 Department of Anesthesiology, The Third Xiangya Hospital, Central South University, Changsha 410013, China; 7 Department of Anaesthesiology, Beijing Obstetrics and Gynaecology Hospital, Capital Medical University, Beijing, China; 8 Department of Anesthesiology, The Affiliated Zhongshan Hospital of Dalian University, Dalian, Liaoning Province 116001, China; 9 Department of Anesthesiology, The First Hospital, Jilin University, Changchun, China; 10 Department of Anesthesiology, Clinical Medical College of Yangzhou University (Northern Jiangsu People’s Hospital), Yangzhou 225001, China; 11 Department of Anesthesiology & Center for Brain Science, The First Affiliated Hospital of Xi’an Jiaotong University, Xi’an, Shaanxi, China; 12 Department of Anesthesiology, Maternal and Child Health Hospital of Xinjiang Uyghur Autonomous Region, Urumqi, Xinjiang, Uygur Autonomous Region, China; 13 Department of Anesthesiology, Nanning Second People’s Hospital, Nanning 530031, China; 14 Department of Anesthesiology, Shengjing Hospital of China Medical University, Shenyang, China; 15 Department of Anesthesiology, Tianjin Medical University General Hospital, Tianjin 300052, China; 16 Department of Anesthesiology, The Second Affiliated Hospital of Chongqing Medical University, Chongqing 400010, China; 17 Department of Anesthesiology, The Second People’s Hospital of Yibin, Yibin, China; 18 State Key Laboratory of Pharmaceutical Biotechnology, Department of Medicine, The University of Hong Kong, Pokfulam, Hong Kong, China; 19 Department of Anesthesiology, Affiliated Hospital of Guangdong Medical University, Zhanjiang, Guangdong, China; 20 Department of Anesthesiology and Perioperative Medicine, People’s Hospital of Zhengzhou University, Henan Provincial People’s Hospital, Zhengzhou, Henan, China; 21 Department of Anesthesiology and Critical Care Medicine, Tianjin Nankai Hospital, Tianjin Medical University, Tianjin, China

**Keywords:** tegileridine, opioid, postoperative pain, patient-controlled analgesia, morphine, analgesia, abdominal surgery, opioid receptor

## Abstract

Tegileridine is a biased μ-opioid receptor agonist that selectively activates the G protein pathway, designed to provide analgesia with fewer opioid-related adverse effects. In this phase 3 study, 526 patients with postoperative pain after abdominal surgery were randomized and received placebo, tegileridine at 0.75 mg, tegileridine at 1.0 mg, or morphine. For the primary outcome of effective analgesia, the mean (SD) summed pain intensity difference at rest over the first 24 h from time 0 (SPID_24_) scores are −61.15 (28.25) and −68.98 (30.33) for the 0.5 and 0.75 mg doses of tegileridine, respectively, compared with −49.63 (29.35) in the placebo group (all *p* < 0.001) and −71.16 (34.76) in the morphine group. The total pain relief scores (mean [SD]) at 24 h were 58.76 (21.79) with 0.75 mg tegileridine and 61.95 (18.94) with 1.0 mg tegileridine, compared with 47.56 (21.00) with placebo and 59.09 (19.34) with morphine. In summary, tegileridine provides effective analgesia that was significantly superior to placebo and comparable to morphine.

## Highlights

• Tegileridine, a biased MOR agonist, selectively activates the G protein pathway
• This trial assessed tegileridine for moderate-to-severe acute postoperative pain
• Tegileridine was superior to placebo in analgesia and was comparable to morphine

## Graphical abstract

**Figure:**
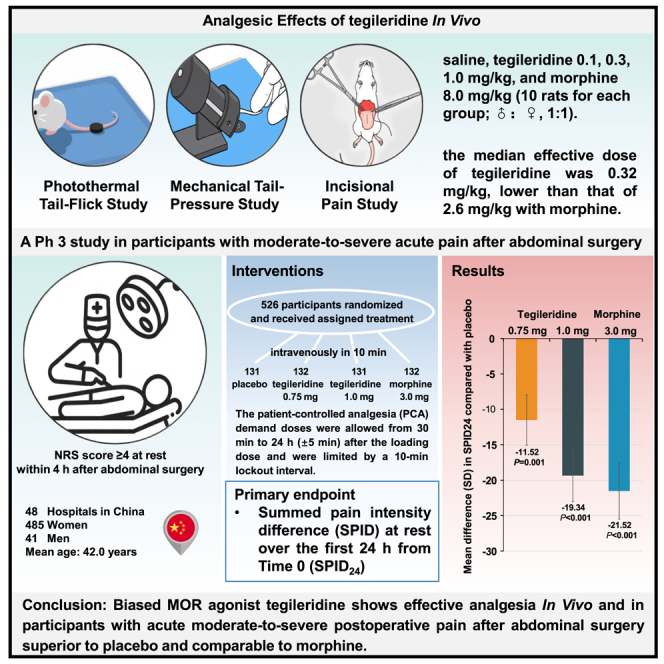

## Teaser

Wang et al. demonstrate the clinical efficacy of tegileridine, an MOR-biased agonist, which is comparable to morphine. They provide data on analgesic effects of tegileridine *in vivo* (in rats) and in participants with moderate-to-severe acute pain after abdominal surgery with an acceptable safety profile.

## Introduction

Approximately 75% of patients experience moderate-to-severe acute postoperative pain, and 50% do not achieve adequate pain relief.^1^^,^^2^ Opioids represent the primary therapeutic modality for managing postsurgical pain, although new analgesics are increasingly being discovered.^3^ Traditional opioids such as morphine are balanced μ-opioid receptor (MOR) agonists that activate both G protein and β-arrestin-2 pathways.^4^ The G protein pathway has been associated with rapid and effective analgesia, whereas the β-arrestin-2 pathway is implicated in opioid-related adverse effects, including gastrointestinal and respiratory dysfunctions.^2^^,^^5^^,^^6^ Opioid-related adverse effects can increase costs, prolong hospital stays, and decrease survival during hospital resuscitation if not effectively managed.^7^^,^^8^

Mice lacking β-arrestin-2 expression demonstrated improved analgesia and reduced side effects after morphine administration.^9^^,^^10^ The biased MOR ligand oliceridine was approved to manage adult acute pain severe enough to require intravenous opioid analgesics based on its superior analgesia over placebo and potentially fewer adverse effects than opioids.^11^^,^^12^^,^^13^ However, the medication is not available to all patients globally.

Tegileridine (SHR8554) is a new biased MOR agonist that selectively activates the G protein pathway. The effect of tegileridine on activation of the β-arrestin-2 signaling pathway was approximately 10% of that of morphine. Previous phase 2 studies involving participants undergoing laparoscopy and orthopedic surgeries have demonstrated the superiority of tegileridine over placebo, coupled with a favorable safety profile (NCT04794738). We further conducted a phase 3 study to assess the efficacy and safety of tegileridine in Chinese participants with moderate-to-severe acute pain after abdominal surgery. This report presents the findings of this phase 3 study, along with the preclinical studies of tegileridine.

## Results

### Analgesic effects of tegileridine *in vivo*

The chemical structure of tegileridine is shown in Figure S1. The effective dose of tegileridine was 0.3 mg/kg for photothermal and mechanical pain in rats, and the analgesic effect of 1.0 mg/kg tegileridine was comparable to that of 8.0 mg/kg morphine (equieffective dose ratio of tegileridine to morphine, 1:8; Figures S2A and S2B). Tegileridine also demonstrated analgesic effects on incisional pain in rats (Figure S2C), and the median effective dose of tegileridine was 0.32 mg/kg, lower than that of 2.6 mg/kg with morphine (equieffective dose ratio of tegileridine to morphine, approximately 1:9).

### Demographic and baseline clinical characteristics

**Figure 1:**
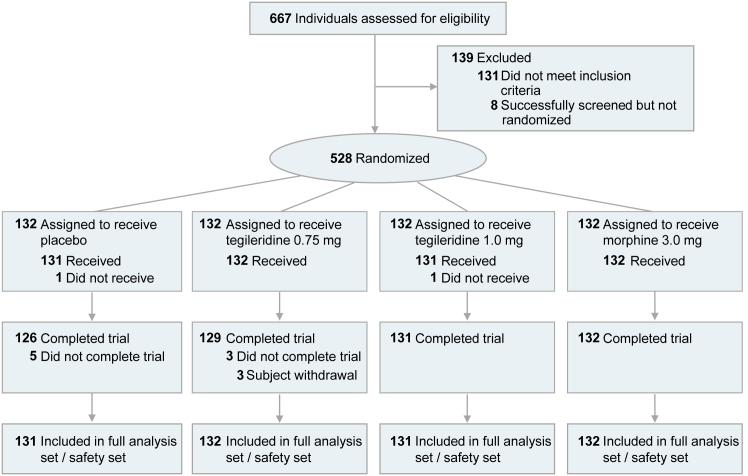
CONSORT flow diagram One participant allocated to the placebo group did not receive the study medication due to investigation decision. The investigator deemed the participant ineligible to continue in the study because the Fridericia-corrected QT (QTC_f_) interval was 500 ms. One participant allocated to the 1.0 mg tegileridine group decided to withdraw from the study and did not receive the study medication.

A total of 667 participants were screened in the phase 3 study, and 528 eligible participants from 48 hospitals in China (Table S1) were randomly assigned to receive placebo, tegileridine at 0.75 mg, tegileridine at 1.0 mg, or morphine (132 each). Of these, 526 were treated with the assigned study medications (131, 132, 131, and 132 in the four groups, respectively). The majority of the participants (518, 98.5%) completed the study (Figure 1; Table S2).

The demographic data and baseline disease characteristics were generally balanced across all groups (Table 1). This study was designed to enroll participants with American Society of Anesthesiologists classification of 1–2, mainly due to considerations of participant safety, study population homogeneity, and reliability of results. A large proportion of the patients enrolled in this study underwent gynecological surgery, and these patients were mostly young, non-obese females.

**Table 1:** Demographic and baseline characteristics

Characteristics	Placebo (*n* = 131)	Tegileridine 0.75 mg (*n* = 132)	Tegileridine 1.0 mg (*n* = 131)	Morphine (*n* = 132)
Age, mean (SD), years	40.8 (10.5)	42.7 (9.2)	43.2 (9.4)	41.2 (9.7)

**Sex, no. (%)**				

Female	116 (88.5)	121 (91.7)	122 (93.1)	126 (95.5)
Male	15 (11.5)	11 (8.3)	9 (6.9)	6 (4.5)

**Ethnicity, no. (%)**				

Han	117 (89.3)	121 (91.7)	121 (92.4)	121 (91.7)
Other	14 (10.7)	11 (8.3)	10 (7.6)	11 (8.3)
Height, mean (SD), cm	161.1 (7.1)	161.3 (6.2)	161.1 (6.0)	160.6 (6.0)
Weight, mean (SD), kg	60.3 (8.4)	60.3 (8.3)	60.3 (8.1)	59.9 (8.1)
BMI, mean (SD), kg/m^2^	23.2 (2.5)	23.1 (2.5)	23.2 (2.4)	23.2 (2.5)

**ASA Physical Status Classification System class, no. (%)**				

1	57 (43.5)	38 (28.8)	48 (36.6)	53 (40.2)
2	74 (56.5)	94 (71.2)	83 (63.4)	79 (59.8)

**Anesthetic method, no. (%)**				

Total intravenous anesthesia	26 (19.8)	31 (23.5)	37 (28.2)	27 (20.5)
Combined intravenous-inhalation anesthesia	105 (80.2)	101 (76.5)	94 (71.8)	105 (79.5)

**Type of abdominal surgery, no. (%)**				

Laparoscopy	112 (85.5)	114 (86.4)	111 (84.7)	111 (84.1)
Laparotomy	19 (14.5)	18 (13.6)	20 (15.3)	21 (15.9)
Duration of surgery, median (IQR), min	100.0 (74.0–131.0)	100.0 (76.5–127.5)	106.0 (78.0–140.0)	96.0 (75.5–137.0)

Abbreviations: ASA, American Society of Anesthesiologists; BMI, body mass index (calculated as weight in kilograms divided by height in meters squared); IQR, interquartile range.

There were no missing data.

### Primary outcome

#### Summed pain intensity difference at rest over the first 24 h from time 0

The pain relief dynamics over time are shown in Figure S3. The mean (SD) summed pain intensity difference at rest over the first 24 h from time 0 (SPID_24_) in the placebo, 0.75 mg tegileridine, 1.0 mg tegileridine, and morphine groups was −49.63 (29.35), −61.15 (28.25), −68.98 (30.33), and −71.16 (34.76), respectively (Table 2; Table S3). The primary endpoint was met, as demonstrated by the statistically significant reduction in SPID_24_ after the administration of tegileridine relative to placebo (0.75 mg tegileridine vs. placebo: difference [SD], −11.52 [3.55]; 95% confidence interval [CI], −18.52 to −4.53; *p* = 0.001; 1.0 mg tegileridine vs. placebo: −19.34 [3.69]; −26.60 to −12.08; *p* < 0.001). Tegileridine at 1.0 mg showed a similar magnitude of effect in decreasing SPID_24_ compared with morphine, whereas the SPID_24_ decrease with tegileridine at 0.75 mg was numerically lower.

**Table 2:** Primary and key secondary efficacy outcomes (SPID and TOTPAR)

Measures	Placebo	Tegileridine 0.75 mg	Tegileridine 1.0 mg	Morphine
	(*n* = 131)	(*n* = 132)	(*n* = 131)	(*n* = 132)
Baseline NRS score^a^, mean (SD)	4.8 (1.0)	4.7 (1.0)	5.0 (1.2)	5.1 (1.4)
Primary endpoint	–	–	–	–

**SPID**_**24**_				

Mean (SD)	−49.63 (29.35)	−61.15 (28.25)	−68.98 (30.33)	−71.16 (34.76)
Difference with placebo (SD)	ND	−11.52 (3.55)	−19.34 (3.69)	−21.52 (3.97)
95% CI; *p*	ND	(−18.52 to −4.53); *p* = 0.001	(−26.60 to −12.08); *p* < 0.001	(−29.34 to −13.71); *p* < 0.001
**Key secondary efficacy endpoints**	–	–	–	–

**SPID**_**6**_				

Mean (SD)	−5.25 (9.23)	−11.91 (8.74)	−14.51 (7.43)	−12.44 (9.46)
Difference with placebo (SD)	ND	−6.66 (1.11)	−9.25 (1.04)	−7.19 (1.15)
95% CI; *p*	ND	(−8.84 to −4.48); *p* < 0.001	(−11.29 to −7.21); *p* < 0.001	(−9.46 to −4.92); *p* < 0.001

**SPID**_**12**_				

Mean (SD)	−17.44 (14.70)	−26.02 (14.89)	−30.23 (14.53)	−30.11 (17.04)
Difference with placebo (SD)	ND	−8.58 (1.82)	−12.79 (1.81)	−12.67 (1.96)
95% CI; *p*	ND	(−12.18 to −4.99); *p* < 0.001	(−16.35 to −9.24); *p* < 0.001	(−16.54 to −8.81); *p* < 0.001

**SPID**_**18**_				

Mean (SD)	−31.91 (21.61)	−42.29 (20.91)	−48.64 (21.62)	−48.61 (25.50)
Difference with placebo (SD)	ND	−10.38 (2.62)	−16.73 (2.67)	−16.70 (2.92)
95% CI; *p*	ND	(−15.54 to −5.22); *p* < 0.001	(−21.99 to −11.48); *p* < 0.001	(−22.44 to −10.96); *p* < 0.001

**SPID**_**12–24**_				

Mean (SD)	−32.20 (17.54)	−35.10 (15.80)	−38.70 (17.44)	−41.00 (19.78)
Difference with placebo (SD)	ND	−2.94 (2.06)	−6.55 (2.16)	−8.85 (2.31)
95% CI; *p*	ND	(−6.99 to 1.11); *p* = 0.155	(−10.80 to −2.29); *p* = 0.003	(−13.39 to −4.31); *p* < 0.001

**TOTPAR**_**6**_				

Mean (SD)	6.29 (6.13)	11.94 (6.59)	13.39 (5.73)	10.85 (6.65)
Difference with placebo (SD)	ND	5.64 (0.78)	7.10 (0.73)	4.56 (0.79)
95% CI; *p*	ND	(4.10–7.19); *p* < 0.001	(5.65–8.54); *p* < 0.001	(3.00–6.11); *p* < 0.001

**TOTPAR**_**12**_				

Mean (SD)	18.29 (10.90)	25.94 (11.99)	28.47 (10.53)	25.73 (10.76)
Difference with placebo (SD)	ND	7.64 (1.41)	10.18 (1.32)	7.44 (1.34)
95% CI; *p*	ND	(4.86–10.43); *p* < 0.001	(7.57–12.79); *p* < 0.001	(4.81–10.07); *p* < 0.001

**TOTPAR**_**18**_				

Mean (SD)	32.31 (15.98)	41.80 (16.99)	44.78 (14.74)	41.68 (15.10)
Difference with placebo (SD)	ND	9.49 (2.03)	12.47 (1.90)	9.38 (1.92)
95% CI; *p*	ND	(5.49–13.50); *p* < 0.001	(8.73–16.21); *p* < 0.001	(5.60–13.15); *p* < 0.001

**TOTPAR**_**24**_				

Mean (SD)	47.56 (21.00)	58.76 (21.79)	61.95 (18.94)	59.09 (19.34)
Difference with placebo (SD)	ND	11.20 (2.64)	14.39 (2.47)	11.53 (2.49)
95% CI; *p*	ND	(6.00–16.39); *p* < 0.001	(9.53–19.26); *p* < 0.001	(6.63–16.43); *p* < 0.001

**TOTPAR**_**12–24**_				

Mean (SD)	29.30 (12.48)	32.80 (11.45)	33.50 (10.36)	33.40 (10.62)
Difference with placebo (SD)	ND	3.55 (1.48)	4.21 (1.42)	4.10 (1.43)
95% CI; *p*	ND	(0.64–6.46); *p* = 0.017	(1.42–7.00); *p* = 0.003	(1.28–6.91); *p* = 0.005

Data were analyzed in all randomized participants who received study treatments. There were no missing data for baseline NRS score. All missing data (Table S3) for SPID and TOTPAR were imputed.

Comparisons of tegileridine or morphine with placebo were conducted using group *t* test. *p* < 0.05 for the primary endpoint (SPID_24_) for the tegileridine and placebo groups was considered to be statistically significant. *p* values for other comparisons were nominal.

Abbreviations: SPID, summed pain intensity difference; TOTPAR, total pain relief score; NRS, numerical rating scale; ND, no data.

a The baseline NRS score represents the NRS score after surgery and before the administration of study treatment.

The sensitivity analysis for the intention-to-treat population was consistent with the primary analysis for SPID_24_ conducted in the full analysis set (Table S4). The sensitivity analysis without imputation for scores after rescue analgesia also revealed a statistically significant difference in SPID_24_ between 1.0 mg tegileridine and placebo groups; although the difference between the 0.75 mg tegileridine and placebo groups was not statistically significant, the mean point estimate with 0.75 mg tegileridine was numerically superior to placebo (Table S4).

### Secondary efficacy outcomes

#### SPID_6_, SPID_12_, SPID_18_, and SPID_12–24_

When compared to placebo, the mean difference (SD) in SPID_6_ with 0.75 mg tegileridine, 1.0 mg tegileridine, and morphine was −6.66 (1.11), −9.25 (1.04), and −7.19 (1.15), respectively; the mean decrease (SD) in SPID_12_ was −8.58 (1.82), −12.79 (1.81), and −12.67 (1.96), respectively; and the mean decrease (SD) in SPID_18_ was −10.38 (2.62), −16.73 (2.67), and −16.70 (2.92), respectively (all nominal *p* < 0.05; Table 2). The effects of 1.0 mg tegileridine and morphine on SPID_6_, SPID_12_, and SPID_18_ were comparable, whereas the effects of 0.75 mg tegileridine were numerically lesser than those of morphine.

The 1.0 mg tegileridine and morphine groups led to greater reductions in SPID_12–24_ than placebo (nominal *p* = 0.003 and <0.001, respectively). However, 0.75 mg tegileridine did not (Table 2).

### Total pain relief score

The mean difference (SD) in total pain relief score (TOTPAR)_24_ was 11.20 (2.64), 14.39 (2.47), and 11.53 (2.49) in the 0.75 mg tegileridine, 1.0 mg tegileridine, and morphine groups, when compared with the placebo group (Table 2). Tegileridine and morphine also showed superiority in comparison with placebo in terms of TOTPAR_6_, TOTPAR_12_, TOTPAR_18_, and TOTPAR_12–24_ (all nominal *p* < 0.05; Table 2). The increase in TOTPAR with the administration of 1.0 and 0.75 mg tegileridine was higher and comparable to that of morphine, respectively.

### Usage of rescue medication within 24 h post loading dose administration

**Figure 2:**
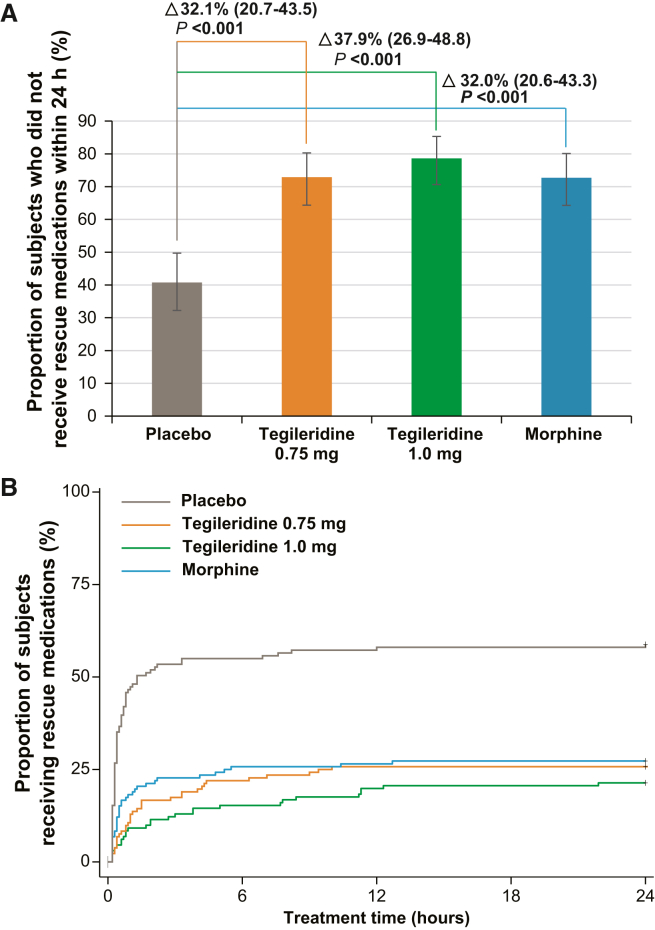
Usage of rescue medication (A) The proportion of participants who did not receive rescue medications in the first 24 h after loading dose administration. The data are presented as percentage (95% CI) for each group and for the differences between groups. Comparisons between groups were conducted using Fisher’s exact test. All *p* values were nominal. (B) Kaplan-Meier plot of first use of rescue medication. Comparisons between groups were conducted using log rank. A participant who used rescue medication at 24 h and 5 min is not shown in the figure.

The proportions of participants who did not need rescue medications were higher in the tegileridine and morphine groups than that in the placebo group: 72.9% (95% CI, 64.3–80.3) for 0.75 mg tegileridine, 78.6% (95% CI, 70.6–85.3) for 1.0 mg tegileridine, 72.7% (95% CI, 64.3–80.1) for morphine, and 40.8% (95% CI, 32.2–49.7) for placebo (all nominal *p* < 0.05; Figure 2A).

The times to first use of rescue medication in the 0.75 mg tegileridine, 1.0 mg tegileridine, and morphine groups were longer than those in the placebo group (Figure 2B).

The mean cumulative consumption of rescue medications (parecoxib sodium and sufentanil) was reduced in the 0.75 mg tegileridine, 1.0 mg tegileridine, and morphine groups relative to the placebo group (Table S5).

### Participant and investigator satisfaction scores at 24 h post dose

The median (interquartile range [IQR]) participant satisfaction scores in the 0.75 mg tegileridine, 1.0 mg tegileridine, and morphine groups were 9.0 (8.0–10.0), 10.0 (8.0–10.0), and 10.0 (9.0–10.0), respectively, compared with 9.0 (8.0–10.0) in the placebo group (Table S6).

The median (IQR) investigator satisfaction scores in the 0.75 mg tegileridine, 1.0 mg tegileridine, and morphine groups were 9.0 (8.0–10.0), 9.0 (8.0–10.0), and 9.0 (8.0–10.0), respectively, compared with 8.0 (7.0–9.0) in the placebo group (Table S6).

### Post hoc analyses

#### Proportion of participants who had numerical rating scale scores of ≤3 during the first 6 h after the loading dose

The proportions of participants with numerical rating scale (NRS) pain scores of ≤3 were higher in the tegileridine and morphine groups than those in the placebo group at each time point during the first 6 h. The proportions in the 1.0 and 0.75 mg tegileridine groups were higher than those in the morphine group during the first 2 h. The proportion in the 1.0 mg tegileridine group was higher than that in the morphine group from 3 to 6 h, whereas the 0.75 mg tegileridine and morphine groups had similar values (Figure S4).

### Safety outcomes

#### Summary

Treatment-emergent adverse events (TEAEs) were reported in 74.0% (97/131), 77.3% (102/132), 77.9% (102/131), and 75.0% (99/132) patients in the placebo, 0.75 mg tegileridine, 1.0 mg tegileridine, and morphine groups, respectively (Table 3). Treatment-related adverse events (AEs) occurred in 42.0% (55/131), 47.0% (62/132), 48.9% (64/131), and 49.2% (65/132) of patients in the four groups, respectively.

**Table 3:** Summary of TEAEs

Measures	Placebo	Tegileridine 0.75 mg	Tegileridine 1.0 mg	Morphine
	(*n* = 131)	(*n* = 132)	(*n* = 131)	(*n* = 132)
Any TEAEs	97 (74.0)	102 (77.3)	102 (77.9)	99 (75.0)
Serious TEAEs	1 (0.8)	0	0	1 (0.8)
TEAEs leading to study discontinuation	0	0	0	0
TEAEs of special interest	42 (32.1)	45 (34.1)	48 (36.6)	51 (38.6)

**TEAEs in severity**				

Mild	60 (45.8)	56 (42.7)	54 (41.2)	55 (42.0)
Moderate	37 (28.2)	46 (35.1)	48 (36.6)	43 (32.8)
Severe	0	0	0	1 (0.8)

**Most common TEAE**^a^				

Nausea	30 (22.9)	29 (22.0)	25 (19.1)	32 (24.2)
Vomiting	27 (20.6)	31 (23.5)	29 (22.1)	38 (28.8)
Abdominal distension	16 (12.2)	10 (7.6)	7 (5.3)	15 (11.4)
Anemia	13 (9.9)	28 (21.2)	22 (16.8)	22 (16.7)
Pyrexia	11 (8.4)	8 (6.1)	14 (10.7)	14 (10.6)
Blood potassium decreased	8 (6.1)	6 (4.5)	4 (3.1)	2 (1.5)
Urine ketone body present	8 (6.1)	5 (3.8)	3 (2.3)	8 (6.1)
Hematuria	7 (5.3)	2 (1.5)	4 (3.1)	3 (2.3)
Hypokalemia	6 (4.6)	10 (7.6)	10 (7.6)	10 (7.6)
Neutrophil count increased	5 (3.8)	10 (7.6)	7 (5.3)	6 (4.5)
Hypoproteinemia	5 (3.8)	7 (5.3)	3 (2.3)	7 (5.3)
Cough	5 (3.8)	3 (2.3)	7 (5.3)	2 (1.5)
Productive cough	4 (3.1)	0	7 (5.3)	2 (1.5)
White blood cell count increased	3 (2.3)	9 (6.8)	6 (4.6)	7 (5.3)
Urinary occult blood: positive	3 (2.3)	8 (6.1)	4 (3.1)	10 (7.6)
Urine red blood cells: positive	3 (2.3)	7 (5.3)	6 (4.6)	10 (7.6)
Blood bilirubin increased	3 (2.3)	5 (3.8)	1 (0.8)	7 (5.3)

Abbreviations: TEAE, treatment-emergent adverse event.

Data were analyzed in all enrolled participants who received study treatments. There were no missing data.

a TEAEs that occurred in ≥5% of participants in either group.

The most common TEAEs were vomiting, nausea, abdominal distension, and anemia (Table 3). All TEAEs were mild or moderate, except for severe vomiting reported by one (0.8%) participant in the morphine group.

Serious adverse events (SAEs) were reported in one (0.8%) participant each in the morphine (increased alanine aminotransferase) and placebo (abdominal infection) groups, and neither of them was deemed treatment related by the investigator. No deaths occurred, and no TEAEs led to discontinuation of the study.

### AEs of special interest

Nausea, vomiting, dizziness, and respiratory depression were AEs of special interest (AESIs) in this study. Overall, 32.1% (42/131), 34.1% (45/132), 36.6% (48/131), and 38.6% (51/132) of patients in the placebo, 0.75 mg tegileridine, 1.0 mg tegileridine, and morphine groups reported at least one AESI, respectively. Specifically, the percentage of participants who experienced individual AESIs was similar across the groups (Table S7).

No obvious differences were found among the respiratory rates of the groups after treatment (Table S8). Only one (0.8%) participant in the 0.75 mg tegileridine group reported respiratory depression (deemed treatment related by the investigator). The transdermal oxygen saturation (SpO_2_) of this participant was 89% during the treatment period (from the start of the loading dose injection to the end of patient-controlled analgesia [PCA] use), which increased to 99% after 4 min without any treatment.

### Usage of antiemetic drugs

Thirty-two (24.4%), 35 (26.5%), 33 (25.2%), and 43 (32.6%) participants in the placebo, 0.75 mg tegileridine, 1.0 mg tegileridine, and morphine groups, respectively, used the antiemetic drug with tropisetron during the treatment period (from the start of the loading dose injection to the end of PCA use). The mean (SD) cumulative dosages for all treated participants were 0.94 (1.85), 0.87 (1.63), 0.91 (1.89), and 1.15 (2.02) mg (dosage for participants who did not use tropisetron was calculated as 0), respectively.

## Discussion

Tegileridine was designed to improve the benefit-risk profile of existing conventional opioids for individuals with moderate-to-severe pain by providing rapid onset of analgesia and sustained pain relief while reducing opioid-induced adverse effects. This randomized, double-blind, placebo- and active-controlled phase 3 study assessed the efficacy and safety of tegileridine in participants with moderate-to-severe pain after abdominal surgery, a recognized and extensively used soft tissue pain model.

Tegileridine at both dosages showed statistically significant superiority to placebo in the primary endpoint of SPID_24_. The sensitivity analysis without imputation for the scores after rescue analgesia showed a similar result for the comparison in SPID_24_ between 1.0 mg tegileridine and placebo, whereas the difference between 0.75 mg tegileridine and placebo was not statistically significant. This was mainly because more participants in the placebo group received rescue analgesia and had higher cumulative consumption of rescue medication, which resulted in a greater degree of post-rescue pain intensity score improvement with placebo. Nonetheless, the mean point estimate of SPID_24_ with 0.75 mg tegileridine was still numerically superior to placebo.

The results of the secondary endpoints support the primary endpoint, as evidenced by the improved SPID and TOTPAR at different time points and time intervals, higher proportion of participants who did not require rescue medications, prolonged time to first use of rescue medications, and reduced cumulative consumption of rescue medication with tegileridine relative to placebo. Generally, compared with morphine, 1.0 mg tegileridine showed a comparable magnitude of effect in decreasing SPID and a numerically higher increase in TOTPAR, while 0.75 mg tegileridine did not. Tegileridine and morphine showed similar proportions of participants not receiving rescue medications and comparable cumulative consumption of rescue medications. These results indicated that tegileridine provides sustained relief of acute post-surgical pain that was comparable in potency to that of morphine.

Notably, the times to the first use of rescue medications were slightly prolonged in the two tegileridine groups compared with the morphine group. The proportions of the participants with NRS pain scores of ≤3 were numerically higher in the 1.0 and 0.75 mg tegileridine groups than those in the morphine group at each time point within the first 2 h after the loading dose administration. These results revealed a potentially faster onset of analgesia with tegileridine than with morphine, which may be attributed to its selectivity for activating the G protein pathway. The analgesic efficacy of tegileridine was observed as early as 10 min after the completion of the loading dose administration.

Both investigators and participants reported higher satisfaction than the placebo group with the 1.0 mg dose of tegileridine. Although satisfaction was high with the 0.75 mg dose among both investigators and participants, only the investigators’ satisfaction reached statistical significance compared to the placebo group. This discrepancy might arise from more participants in the placebo group receiving rescue analgesia and having higher cumulative consumption of rescue medication. In addition, there were distinct criteria between investigators and participants for evaluating pain treatment satisfaction. Investigators’ satisfaction primarily focused on objective measures, such as the degree of reduction in pain scores, the need for rescue medications, and the percentage of participants experiencing AEs. In contrast, participants’ satisfaction was multidimensional and experiential, mainly affected by their expectations for pain management, their engagement in treatment, their satisfaction with AE management, and whether decrease in pain scores can improve their sleep, emotions, and physical function.

Tegileridine was safe and well tolerated. There were no reported deaths or TEAEs leading to study withdrawal, and no SAEs occurred in the tegileridine groups. The side effects of classic opioids, including respiratory depression and gastrointestinal dysfunction (such as nausea and vomiting), precluded more effective dosing in the postoperative setting. Tegileridine was hypothesized to attenuate these side effects as a G protein-biased ligand of the MOR. In this study, the percentages of participants who experienced nausea and vomiting were similar between the tegileridine and morphine groups. However, the percentage of participants experiencing moderate or severe vomiting was slightly lowered by 3.8% in the tegileridine groups compared with the morphine group (moderate, 12.9% vs. 15.9%; severe, 0 vs. 0.8%). No group differences were detected statistically. Moreover, the percentage of participants requiring antiemetic drugs during the treatment period decreased by 6.7% in the tegileridine groups relative to that of the morphine group (25.9% vs. 32.6%). Our gastrointestinal safety findings were consistent with those of oliceridine.^12^^,^^13^ Only one participant in the 0.75 mg tegileridine group reported respiratory depression during treatment. This precluded robust comparisons and conclusions. One possible reason for the very rare respiratory depression was the permitted use of prophylactic oxygen therapy, which confounded the interpretation of the results. The percentage of participants who experienced dizziness was low, and all the events were mild or moderate in severity; however, considering the safety findings from previous phase 1 studies, dizziness should be monitored closely in future tegileridine studies. Pruritus was extremely infrequent, with the same percentage in the tegileridine and morphine groups. In addition, no participants in the study experienced sedation. Further long-term investigations are required to explore the safety advantages of tegileridine relative to classic opioids.

In conclusion, this study demonstrated that tegileridine provided effective analgesia that was significantly superior to placebo and comparable to morphine. Tegileridine may have a more favorable benefit-risk profile than conventional opioids. Tegileridine, as an MOR-biased agonist that selectively activates the G protein pathway, is promising for the management of postoperative acute moderate-to-severe pain under sufficient post-anesthesia care unit monitoring. Based on the findings of this study, tegileridine has been approved for the management of moderate-to-severe acute pain following abdominal surgery in China.

### Limitations of the study

This study had several limitations. First, it was not powered to compare the safety between tegleridine and morphine. Second, this study included a 24-h treatment period (with study medications administered as a loading dose followed by infusions via a PCA pump) and a 4-day follow-up period. The analgesic effect of tegileridine requires validation with prescribed repeated doses and long-term follow-up. Third, the potential efficacy and safety of tegileridine in opioid-tolerant and elderly patient populations require further investigation.

## Resource availability

### Lead contact

Further information and requests for resources and reagents should be directed to and will be fulfilled by the lead contact, Dr. Xiangdong Chen (xdchen@hust.edu.cn).

### Materials availability

This study did not generate new unique reagents.

### Data and code availability

• All data generated in this study are included in this published article. All other relevant detailed clinical data can be available upon reasonable request from the lead contact.
• No custom computer codes are reported in this paper. The code for data analysis is available upon request.
• Any additional information required to reanalyze the data reported in this work paper is available from the lead contact upon request.

## Acknowledgments

This study was funded by 10.13039/100018731Jiangsu Hengrui Pharmaceuticals Co., Ltd.. We thank all participants and their families for participating in this trial, as well as all investigators and site personnel.

## Author contributions

X.C. had full access to all of the data in the study and takes responsibility for the integrity of the data and the accuracy of the data analysis. Concept and design, J.Z., J.Y., and X.C.; drafting of the manuscript, T.W., Y.W., H.X., Z.X., J.Z., J.Y., and X.C.; acquisition, analysis, or interpretation of data, T.W., Y.W., H.X., Z.W., S.Y., Y.O., M.X., W.J., L.G., J.G., Q.W., H.G., Y.H., P.Z., Y.Y., H.H., J.R., J.Z., J.Y., and X.C.; supervision, J.Z., J.Y., and X.C.

## Declaration of interests

The authors declare no competing interests.

## STAR★Methods

### Key resources table

**Table:** 

REAGENT or RESOURCE	SOURCE	IDENTIFIER
**Chemicals, peptides, and recombinant proteins**		

Tegileridine (SHR8554) 1mL:1mg and 5mL:5mg	Jiangsu Hengrui Pharmaceuticals Co, Ltd.	CAS ID 2095345-66-5
Morphine hydrochloride injection (1 mL: 10 mg)	Northeast Pharmaceutical Group Shenyang No.1 Pharmaceutical Co., LTD	Cat#130308-2
0.9% sodium chloride solution	Jiangsu Hengrui Pharmaceuticals Co, Ltd.	N/A

**Software and algorithms**		

SAS	SAS Institute Inc. SAS® Studio. Cary, NC: SAS Institute Inc.	Version 9.4.

### Experimental models and study participant details

#### *In vivo* preclinical studies

The analgesic effects of tegileridine were investigated in rats using the photothermal tail-flick, mechanical tail-pressure, and incisional models. They were compared with those of saline and morphine. Analgesia *in vivo* studies were performed at Shanghai Institute of Materia Medica, Chinese Academy of Sciences (Shanghai, China), with Sprague-Dawley rats (supplied by Shanghai Xipuer-Bikai Laboratory Animal Co., Ltd. (Production License No.: SCXK (Hu) 2013-0016), with the laboratory animal quality certificate serial number 2008001662269) housed in standard conditions with a 12-h light/dark cycle. Ten rats (5 males and 5 females) were aged over 8 weeks prior to dosing, with female rats weighing 162.6–199.3 g and male rats weighing 186.5–227.1 g. The studies were approved by the respective Institutional Animal Care and Use Committees.

#### Human subjects and ethical approval

Eligible participants were men and women aged 18–65 years who were scheduled to undergo abdominal surgery under general anesthesia. Other key inclusion criteria included a surgical duration of at least 1 h, ASA classification of 1–2, and NRS score ≥4 at rest within 4 h after surgery.

The key postoperative exclusion criteria included a previous history of difficult airway; continuous opioid analgesic use for >10 days within 3 months before randomization; SpO_2_ <90% during screening; and use of medications, including selective α2 adrenergic receptor agonists, opioid agonists/antagonists, nonsteroidal anti-inflammatory drugs, sedatives, monoamine oxidase inhibitors, glucocorticoids, CYP2D6, CYP3A4 and CYP3A5 inhibitors/inducers, antihistamines, or antipsychotics, within 5 half-lives before randomization.

The study adhered to the guidelines of Good Clinical Practice and the Helsinki Declaration. The protocol was approved by the institutional review boards of each site, and all participants provided written informed consent.

### Method details

#### Clinical study design

This multicenter, randomized, double-blind, placebo- and active-controlled, phase 3 study was conducted at 48 sites in China from March to November 2021 (NCT04766463). The study was designed in accordance with pain-related clinical trial guidelines and referenced the APOLLO-2 study of oliceridine (an approved biased MOR ligand).^12^^,^^14^^,^^15^^,^^16^

#### Randomization and masking

Eligible participants were randomized (1:1:1:1) to receive placebo, 0.75 mg tegileridine, 1.0 mg tegileridine, or 3.0 mg morphine. Randomization was performed using a centralized interactive web response system. The participants, investigators, and all personnel involved in the study operation or clinical assessment (except the unblinded drug management team) were masked to treatment assignment.

#### Procedures

Tegileridine is a white or almost white powder that is easily soluble in methanol, soluble in anhydrous ethanol and 0.1 mol/L hydrochloric acid, and slightly soluble in water. Tegileridine injection was produced by Jiangsu Hengrui Pharmaceutical Co., Ltd, and diluted with 0.9% sodium chloride before administration. Participants received a loading dose of treatments intravenously over 10 min on day 1 after the randomization (0.75 mg or 1.0 mg tegileridine, 3.0 mg morphine, or volume-matched placebo [0.9% sodium chloride]; all in 5 mL). The single infusion dose via PCA pump was 0.05 mg tegileridine, 0.05 mg tegileridine, 1.0 mg morphine, or a volume-matched placebo (0.9% normal saline) (1 mL). The maximum allowable dose per hour was 0.3 mg tegileridine, 0.3 mg tegileridine, 6.0 mg morphine, or volume-matched placebo (0.9% normal saline) (6 mL). The PCA demand doses were allowed from 30 min to 24 h after the loading dose and were limited by a 10-min lockout interval. The time of administration of the first dose of the study drug was designated as Time 0.

Rescue medications were permitted for inadequate analgesia as determined by the investigator. Parecoxib sodium was the first choice, and sufentanil was administered when the cumulative maximum dose of parecoxib sodium was reached and analgesia was still warranted for desirable PR.

The NRS PI score and PR score were assessed within 5 min before the beginning of the loading dose infusion, immediately after the end of the loading dose infusion, and at 20 min, 30 min, 45 min, 1 h, 1.5 h, 2 h, 3 h, 4 h, 5 h, 6 h, 8 h, 10 h, 12 h, 18 h, and 24 h after completing the loading dose infusion. Safety outcomes were assessed from the screening to Day 4.

#### Outcomes

The primary endpoint was SPID_24_. The secondary endpoints included (1) SPID at 6, 12, and 18 h after the loading dose and SPID from 12 to 24 h post loading dose administration (SPID_6_, SPID_12_, SPID_18_, and SPID_12-24_); (2) TOTPAR_6_, TOTPAR_12_, TOTPAR_18_, TOTPAR_24_, and TOTPAR_12-24_; (3) time to first use of rescue medications; (4) cumulative amount of rescue medications from 0 to 24 h; (5) percentage of participants who did not use rescue medications over 24 h from Time 0; (6) treatment satisfaction scores of the participants and investigators at 24 h; and (7) safety assessments, including TEAEs, laboratory tests, 12-lead ECG, vital signs, SpO_2_, and complete physical examination.

SPID_24_ and TOTPAR_24_ are widely accepted standard measures that integrate the magnitude and duration of relief into a single composite score for evaluating analgesics.^17^^,^^18^^,^^19^ The time-weighted SPID score was measured using a PI NRS ranging from 0 to 10 (0 = no pain, 10 = very severe pain). The difference from the baseline PI score was recorded as PID and calculated using the formula SPIDt = ∑[PIDt∗ (TIMEt-TIMEt-1)](h). For example, SPID_2_=(PI_2_-PI_0_)×(2-1)+(PI_1_-PI_0_)×(1-0.5)+ (PI_0.5_-PI_0_)×(0.5–0). The theoretical SPID_24_ ranged from −240 to 240. A higher absolute value of SPID indicated greater cumulative pain reduction. For TOTPAR, the investigator instructed the participant to assess the degree of PR using the Likert scale ranging from 0 to 4 (0, no response; 1, mild response; 2, moderate response; 3, significant response; 4, complete response). The TOTPAR was calculated according to the formula TOTPARt = ∑[PRt∗ (TIMEt-TIMEt-1)](h). A larger TOTPAR value demonstrated greater PR.

The participants were asked to recall the postoperative analgesic effect and rate their satisfaction on a scale of 0–10 points, with 0 representing dissatisfied and 10 representing very satisfied. The study investigators independently assessed the postoperative analgesic effect in the participant using the same 0-to-10-point scale.

### Quantification and statistical analysis

Data in analgesia *in vivo* studies were 10 rats (5 male and 5 female) for each group of saline, tegileridine 0.1 mg/kg, tegileridine 0.3 mg/kg, tegileridine 1.0 mg/kg, and morphine 8.0 mg/kg treatment groups in the photothermal tail-flick, mechanical tail-pressure, and incisional models. The analysis was used one-way analysis of variance, followed by pairwise comparisons between tegileridine and morphine versus saline at each time point using Tukey’s posthoc multiple comparison test.

In the clinical study, the sample size of 118 per group could provide 90% power to demonstrate the superiority of tegileridine to placebo at a 2-sided α of 0.05, assuming mean (SD) SPID_24_ values of 88 (45) and 70 (40) for the 0.75 mg tegileridine and placebo groups, respectively. The planned total sample size was 528 participants (132 per group) based on a randomization rate of 1:1:1:1 for the placebo, 0.75 mg tegileridine, 1.0 mg tegileridine, and 3.0 mg morphine groups and a dropout rate of 10%.

The efficacy outcomes were analyzed in the full analysis set, which included all randomized participants who received study treatments. The safety outcomes were analyzed in all enrolled participants who received study treatments. A sequential testing approach was used to control the type I error rate at a 2-sided α of 0.05 for the comparisons in the primary endpoint. Comparison in SPID_24_ between 1.0 mg tegileridine versus placebo was done using group *t* test at the 0.05 significance level. If the result was significant, 0.75 mg tegileridine was tested against placebo at the 0.05 significance level; otherwise, only exploratory analysis of 0.75 mg tegileridine versus placebo was performed. No statistical tests were performed for comparisons of the two tegileridine doses and for tegileridine and morphine.

For secondary efficacy outcomes, SPID and TOTPAR were analyzed using method similar to that for SPID_24_. The time to first use of rescue medications was analyzed using the Kaplan-Meier method (for participants who did not use rescue analgesics, the data were censored at 24 h), and comparisons with placebo were conducted using the log rank test. The Wilcoxon rank-sum test was used to analyze the cumulative consumption of rescue medication from 0 to 24 h. The percentages of participants who did not use rescue medication at 24 h were described descriptively, and the 95% CIs were calculated using the Clopper-Pearson method; comparisons to placebo were performed using Fisher’s exact test. The Wilcoxon rank-sum test was used to compare treatment satisfaction scores for the treatment and placebo groups.

When evaluating SPID and TOTPAR, missing data for PI and PR scores were imputed according to the following rules. If a participant did not have a PI score during night sleep, an NRS of 3 was imputed; if a participant did not have a PR score during night sleep, the previous PR score was imputed. For missing data within 6 h after rescue analgesia with parecoxib sodium and within 2 h after rescue analgesia with sufentanil, PI/PR scores at pre-specified time points were imputed using the PI/PR scores recorded before rescue analgesia. In other cases, missing PI/PR scores were imputed using the last observation carried forward method. Missing data for other secondary efficacy variables and safety evaluations were not imputed. In addition, sensitivity analyses for the primary endpoint were done in the full analysis set with scores after rescue analgesia not being imputed and in the intention-to-treat population (which included all randomized participants).

*p* < 0.05 for the primary endpoint between tegileridine and placebo was considered to be statistically significant, and *p* values for other comparisons were nominal. Descriptive statistics were used for safety assessments. All statistical analyses were conducted using SAS version 9.4.

#### Additional resources

This clinical trial has been registered on https://clinicaltrials.gov (NCT04766463).

## Supplemental information

Document S1. Figures S1–S4, Tables S1–S8, and supplemental methods

Data S1. Clinical trial-related documents Data S1 include the protocol and statistical analysis plan. This information is related to STAR Methods.

Data S2. CONSORT checklist

Document S2. Article plus supplemental information
