# Supplementary material for: Tegileridine for moderate-to-severe acute pain following abdominal surgery: A randomized, double-blind, phase 3 clinical trial
Source: Cell Rep Med. 2025 Dec 8;6(12):102477. doi: 10.1016/j.xcrm.2025.102477 (PMC12765830; doi:10.1016/j.xcrm.2025.102477)
Supplement: Document S1. Figures S1–S4, Tables S1–S8, and supplemental methods [file mmc1.pdf]

Cell Reports Medicine, Volume *6*

Supplemental information

Tegileridine for moderate-to-severe acute pain following abdominal surgery: A randomized, double-blind, phase 3 clinical trial

Tingting Wang, Yafeng Wang, Haihui Xie, Zhilin Wu, Shuchun Yu, Yangwen Ou, Mingjun Xu, Wanwei Jiang, Liang Ge, Ju Gao, Qiang Wang, Hexin Gao, Yanjuan Huang, Ping Zhao, Yonghao Yu, He Huang, Jinghua Ren, Zhengyuan Xia, Jiaqiang Zhang, Jianbo Yu, and Xiangdong Chen

Supplemental information

Supplementary Figures

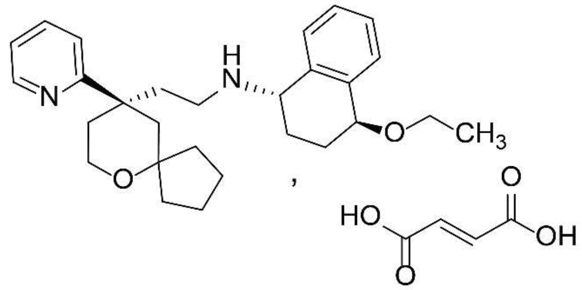

SHR8554 structure
$C_{28}H_{38}N_2O_2 \cdot C_4H_4O_4$

Figure S1. Chemical structure for tegileridine

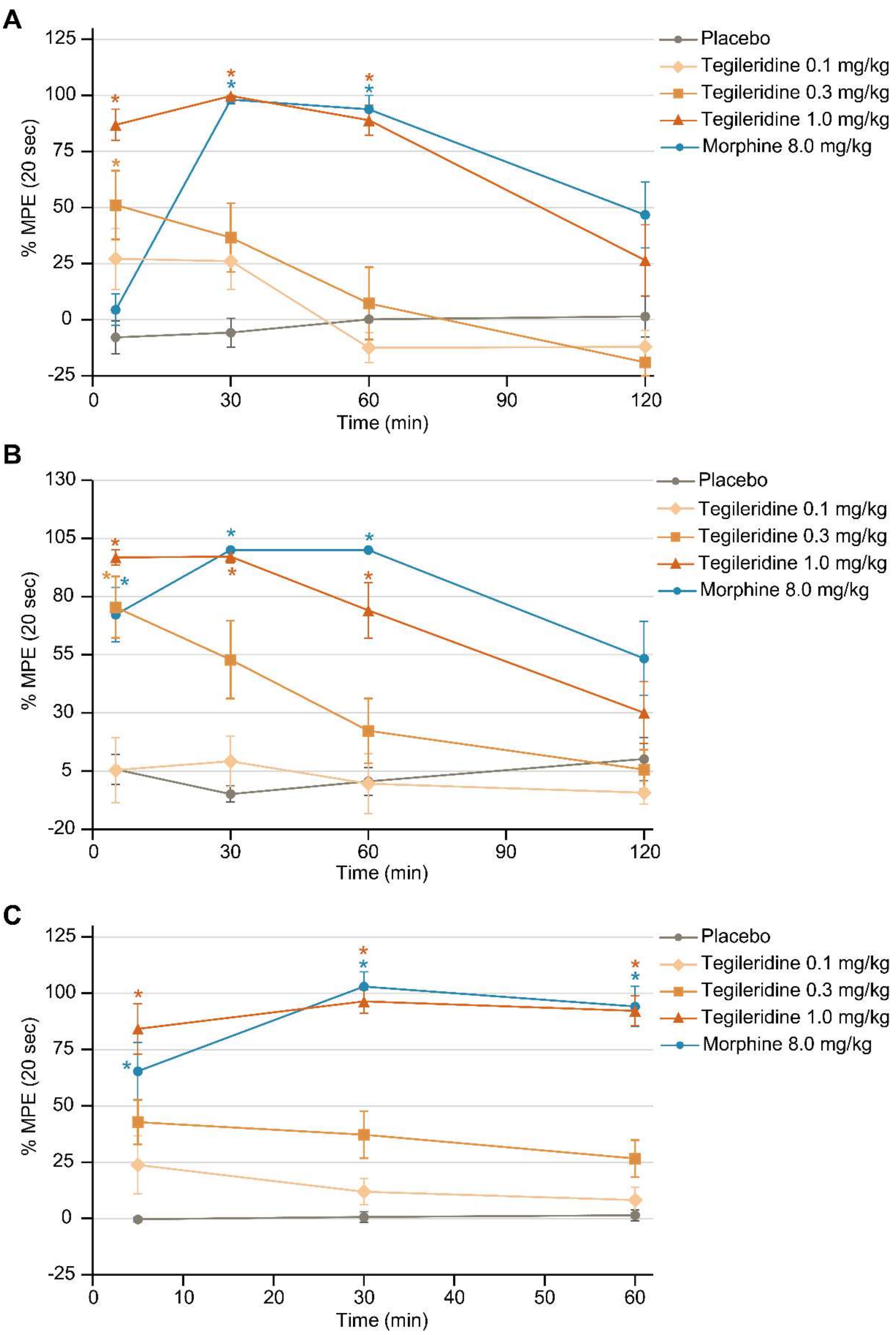

Figure S2. Analgesic effects of tegileridine *in vivo* studies

A, Time-course of tegileridine and morphine analgesic effect on photothermal pain. **B**, Time-course of tegileridine and morphine analgesic effect on mechanical pain. **C**, Time-course of tegileridine and morphine analgesic effect on incision-related pain.

* indicates $P < .05$ versus saline. Data are mean ± SE. Abbreviations: MPE, maximum possible effect.

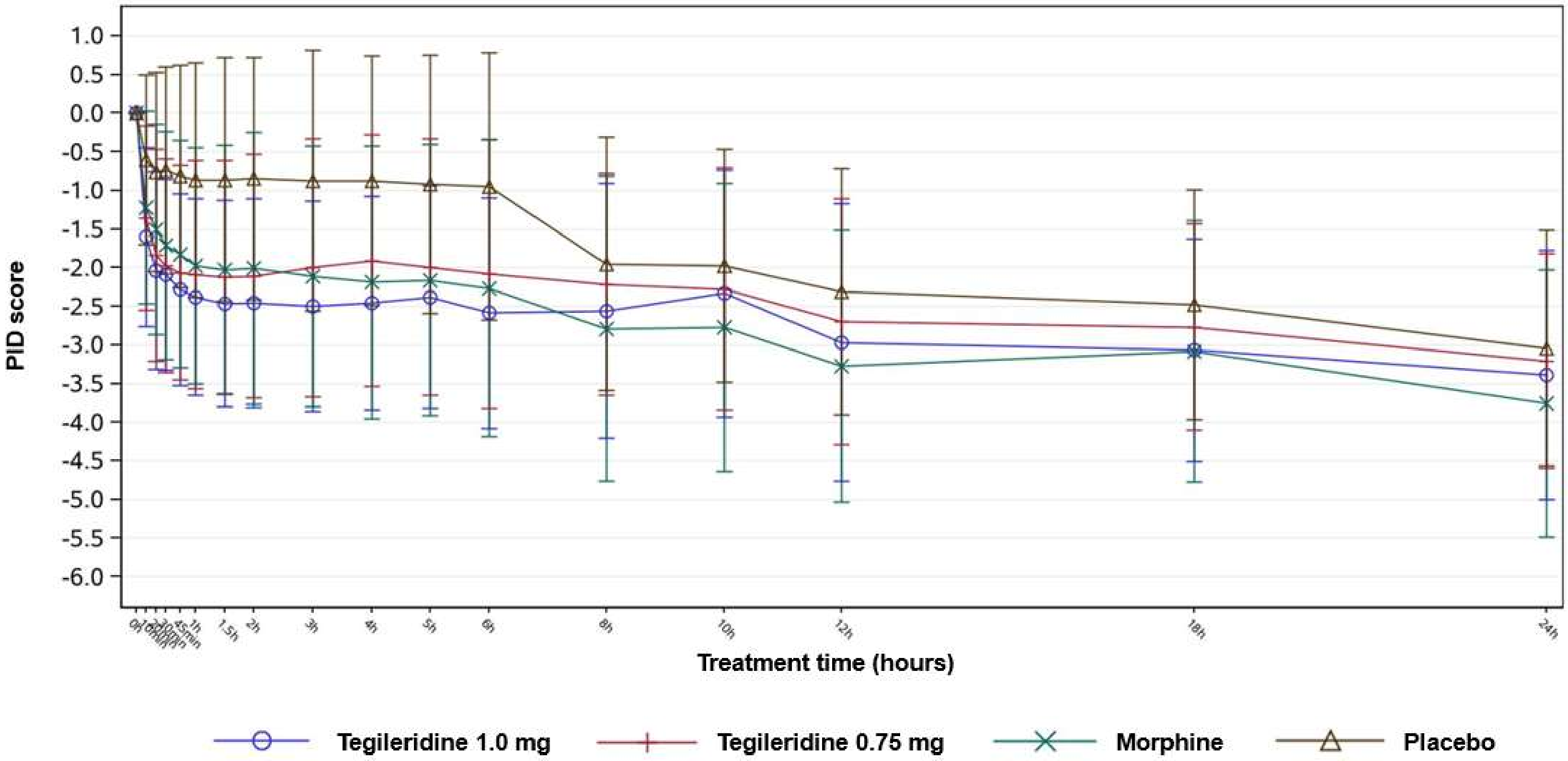

Figure S3. PID over time

PID, pain intensity difference from baseline.

Data are mean (SD).

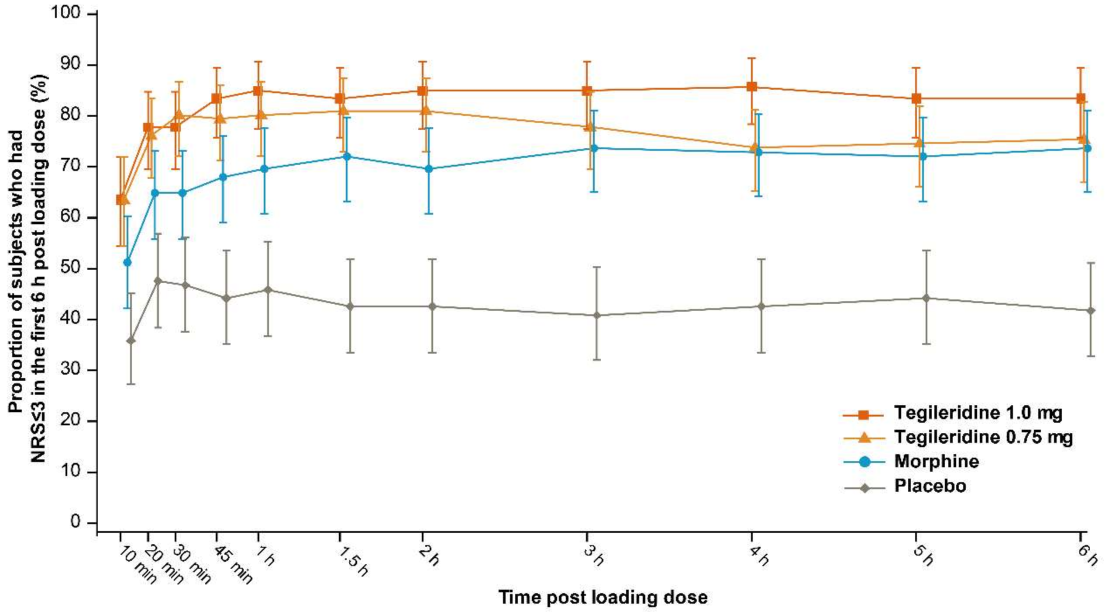

Figure S4. The proportion of subjects who had a pain score of NRS≤3 in the first 6 hours post loading dose

NRS, numerical rating scale.
Data are % (95% CI).

Supplementary Tables

Table S1. List of investigators

Principal investigators	Site name	No. of patients
Chen Xiangdong	Union Hospital, Tongji Medical College, Huazhong University of Science and Technology	14
Yu Shuchun	The Second Affiliated Hospital of Nanchang University	7
Ou Yangwen	The Third Xiangya Hospital, Central South University	9
Zhou Qi	Chifeng Clinical Medical College of inner Mongolia Medical University	6
Xu Mingjun	Beijing Obstetrics and Gynaecology Hospital, Capital Medical University	12
Han Ruquan	Beijing Tiantan Hospital, Capital Medical University.	2
Jiang Wanwei	The Affiliated Zhongshan Hospital of Dalian University	32
Ge Liang	The First Hospital, Jilin University	26
Chen Junping	Ningbo No.2 Hospital	4
Ai Dengbin	Qingdao Municipal Hospital	4
Wang Shoushi	Qingdao Central Hospital	10
Yu Jianbo	Tianjin Nankai Hospital, Tianjin Medical University	25
Xie Haihui	Affiliated Dongguan People's Hospital of Southern Medical University	45
Tian Yi	Haikou People's Hospital	12
Zhao Zhibin	The First People's Hospital of Lianyungang	14
Gao Ju	Northern Jiangsu People's Hospital	15
Sun Canlin	Taizhou People's Hospital	0
Zhang Linzhong	Second Hospital of Shanxi Medical University	4
Wang Qiang	The First Affiliated Hospital of Xi'an Jiaotong University	21
Gao Hexin	Maternal and Child Health Hospital of Xinjiang Uyghur Autonomous Region	24
Min Su	The First Affiliated Hospital of Chongqing Medical University	9
Huang Yanjuan	Nanning Second People's Hospital	20
Guo Huajing	The First People's Hospital of Changde	1
Peng Jian	Wuhan Third Hospital	2
Yan Hong	The Central Hospital of Wuhan	8
Wang Guyan	Beijing TongRen Hospital, Capital Medical University	5
Zhang Longzhen	Meihekou Central Hospital	20
Li Changrong	Siping Central People's Hospital	0
Zhang Benfa	Tonghua Central Hospital	8
Zhaoping	Shengjing Hospital of China Medical University	21
He Huanzhong	Huzhou Central Hospital	10
Wu Cheng	The Second Hospital of Jiaxing	1
Zhou Xuyan	The First Hospital of Jiaxing	6
Liu Huacheng	The Second Affiliated Hospital of Wenzhou Medical University	11

Jia Li	The Fourth Hospital of Hebei Medical University	5
Jin Shuan	Jinan Central Hospital	9
Zhang Nianliang	Rizhao People's Hospital	6
Su Zhen	Huai'an First People's Hospital	1
Gao Yuanli	Ma'anshan People's Hospital	13
Yan Wenjun	Gansu Provincial Hospital	7
Zhang Jiaqiang	People's Hospital of Zhengzhou University, Henan Provincial People's Hospital	8
Han Chongfang	Shanxi Bethune Hospital	8
Jia Jintai	Heping Hospital Affiliated to Changzhi Medical College	10
Lu Jianhua	Liuzhou Worker's Hospital	2
Yu Yonghao	Tianjin Medical University General Hospital	10
Huang He	The Second Affiliated Hospital of Chongqing Medical University	10
Ren Jinghua	The Second People's Hospital of Yibin	22
Jin Hua	The First People's Hospital of Yunnan Province	9

Table S2. Details for participants who received the assigned study medication but did not complete the study

Group	Consent Date	Withdrawal Date	Loading Dose Infusion Amount	PCA Pump Usage Time	Medication Delivered via PCA Pump	Withdrawal Reason
Placebo	Apr 13, 2021	Apr 14, 2021	5 ml	0 min	0 ml	Participant Decision
Placebo	Aug 15, 2021	Aug 18, 2021	5 ml	1 h 34 min	2 ml	Participant Decision
Placebo	Jul 27, 2021	Jul 29, 2021	5 ml	2 h 18 min	8 ml	Participant Decision
Placebo	Oct 8, 2021	Oct 9, 2021	5 ml	4 h 56 min	10 ml	Participant Decision
Placebo	May 10, 2021	May 15, 2021	5 ml	5 h 37 min	22 ml	Participant Decision
0.75 mg tegileridine	Jul 30, 2021	Aug 2, 2021	5 ml	33 min	0 ml	Participant Decision
0.75 mg tegileridine	Apr 20, 2021	Apr 21, 2021	5 ml	3 h 7 min	3 ml	Participant Decision
0.75 mg tegileridine	Aug 1, 2021	Aug 5, 2021	5 ml	3 h 35 min	8 ml	Participant Decision

A total of 8 participants did not complete the study. All these patients received the assigned treatment. They withdrew from the study due to their own decision.

PCA, patient-controlled analgesia.

Table S3. Number of participants who had missing data for NRS PI score and PR score

	Placebo (n=131)	Tegileridine 0.75 mg (n=132)	Tegileridine 1.0 mg (n=131)	Morphine (n=132)
**NRS PI score**				
**Time immediately after the end of the loading dose infusion**				
Missing	0	0	0	0
**20 min after treatment**				
Missing	0	3	2	3
Missing reason: night sleep state	0	3	2	3
**30 min after treatment**				
Missing	0	5	1	3
Missing reason: night sleep state	0	5	1	3
**45 min after treatment**				
Missing	1	5	1	5
Missing reason: night sleep state	1	4	1	5
Missing reason: others	0	1	0	0
**1 h after treatment**				
Missing	2	2	3	2
Missing reason: night sleep state	2	1	3	2
Missing reason: others	0	1	0	0
**1.5 h after treatment**				
Missing	4	4	0	4
Missing reason: night sleep state	4	3	0	4
Missing reason: others	0	1	0	0
**2 h after treatment**				
Missing	2	2	1	2
Missing reason: night sleep state	2	1	1	2
Missing reason: others	0	1	0	0
**3 h after treatment**				
Missing	0	1	2	0
Missing reason: night sleep state	0	0	2	0
Missing reason: others	0	1	0	0
**4 h after treatment**				
Missing	11	12	10	7
Missing reason: night sleep state	9	10	10	7
Missing reason: others	2	2	0	0
**5 h after treatment**				
Missing	17	10	13	14
Missing reason: night sleep state	15	8	13	14
Missing reason: others	2	2	0	0
**6 h after treatment**				
Missing	6	9	4	5
Missing reason: night sleep state	4	6	4	5

Missing reason: others	2	3	0	0
**8 h after treatment**				
Missing	28	33	39	25
Missing reason: night sleep state	23	30	39	25
Missing reason: others	5	3	0	0
**10 h after treatment**				
Missing	43	45	57	50
Missing reason: night sleep state	38	42	57	50
Missing reason: others	5	3	0	0
**12 h after treatment**				
Missing	21	9	6	7
Missing reason: night sleep state	16	6	6	7
Missing reason: others	5	3	0	0
**18 h after treatment**				
Missing	37	32	18	28
Missing reason: night sleep state	32	29	18	28
Missing reason: others	5	3	0	0
**24 h after treatment**				
Missing	5	3	0	0
Missing reason: others	5	3	0	0
**PR score**				
**Time immediately after the end of the loading dose infusion**				
Missing	0	0	0	0
**20 min after treatment**				
Missing	0	3	2	3
Missing reason: night sleep state	0	3	2	3
**30 min after treatment**				
Missing	0	5	1	3
Missing reason: night sleep state	0	5	1	3
**45 min after treatment**				
Missing	1	5	1	5
Missing reason: night sleep state	1	4	1	5
Missing reason: others	0	1	0	0
**1 h after treatment**				
Missing	2	2	3	2
Missing reason: night sleep state	2	1	3	2
Missing reason: others	0	1	0	0
**1.5 h after treatment**				
Missing	4	4	0	4
Missing reason: night sleep state	4	3	0	4
Missing reason: others	0	1	0	0
**2 h after treatment**				
Missing	2	2	1	2
Missing reason: night sleep state	2	1	1	2
Missing reason: others	0	1	0	0

**3 h after treatment**				
Missing	1	1	2	0
Missing reason: night sleep state	0	0	2	0
Missing reason: others	1	1	0	0
**4 h after treatment**				
Missing	11	12	10	7
Missing reason: night sleep state	9	10	10	7
Missing reason: others	2	2	0	0
**5 h after treatment**				
Missing	17	11	12	14
Missing reason: night sleep state	15	8	12	14
Missing reason: others	2	3	0	0
**6 h after treatment**				
Missing	6	9	4	5
Missing reason: night sleep state	4	6	4	5
Missing reason: others	2	3	0	0
**8 h after treatment**				
Missing	28	33	39	25
Missing reason: night sleep state	23	30	39	25
Missing reason: others	5	3	0	0
**10 h after treatment**				
Missing	43	45	58	50
Missing reason: night sleep state	38	42	58	50
Missing reason: others	5	3	0	0
**12 h after treatment**				
Missing	21	9	7	7
Missing reason: night sleep state	16	6	7	7
Missing reason: others	5	3	0	0
**18 h after treatment**				
Missing	37	32	18	28
Missing reason: night sleep state	32	29	18	28
Missing reason: others	5	3	0	0
**24 h after treatment**				
Missing	5	3	0	0
Missing reason: others	5	3	0	0

NRS, numerical rating scale; PI, pain intensity; PR, pain relief.

Table S4. Sensitivity analyses of SPID24

	Placebo (n=131)	Tegileridine 0.75 mg (n=132)	Tegileridine 1.0 mg (n=131)	Morphine (n=132)
**Scores after rescue analgesia not being imputed** [a]				
Mean (SD)	-59.18 (29.37)	-65.06 (27.49)	-72.14 (31.79)	-75.72 (36.29)
Difference with placebo (SD)		-5.88 (3.51)	-12.95 (3.78)	-16.53 (4.07)
95% CI		-12.78 to 1.03	-20.40 to -5.51	-24.55 to -8.51
P value		0.095	<0.001	<0.001
**In the intention-to-treat population** [b]				
Mean (SD)	-49.26 (29.55)	-61.15 (28.25)	-68.46 (30.81)	-71.16 (34.76)
Difference with placebo (SD)		-11.90 (3.56)	-19.20 (3.72)	-21.90 (3.97)
95% CI		-18.90 to -4.89	-26.51 to -11.88	-29.72 to -14.08
P value		0.0010	<0.0001	<0.0001

[a] Participants in the full analysis set were included for analysis. Scores after rescue analgesia used the actually observed values without statistical imputation. Other missing data were imputed following the same rules as the primary analysis for $SPID_{24}$.

[b] Participants in the intention-to-treat population were included for analysis, with the same rules as the primary analysis for $SPID_{24}$.

Data were analyzed in all randomized subjects who received study treatments. All missing data were imputed.

Table S5. Accumulative consumption of rescue medication in the first 24 hours post loading dose

	Placebo (n=130)	Tegileridine 0.75 mg (n=129)	Tegileridine 1.0 mg (n=131)	Morphine (n=132)
**Parecoxib sodium (mg)**				
n (n missing)	130 (1)	129 (3)	131 (0)	132 (0)
Median (IQR)	20.0 (0.0-40.0)	0.0 (0.0-20.0)	0.0 (0.0-20.0)	0.0 (0.0-20.0)
Z; *P*		-5.3557; *P* <0.0001	6.4669; *P* <0.0001	5.5480; *P* <0.0001
**Sufentanil (μg)**				
n (n missing)	128 (3)	129 (3)	131 (0)	132 (0)
Median (IQR)	0.0 (0.0-0.0)	0.0 (0.0-0.0)	0.0 (0.0-0.0)	0.0 (0.0-0.0)
Z; *P*		1.3921; *P* =0.0819	2.4029; *P* =0.0081	2.4253; *P* =0.0076

IQR, interquartile range.

Wilcoxon rank sum test was used.

The dosage for subjects who completed the treatment and did not use rescue medication was calculated as 0.

Table S6. Satisfaction scores at 24 h post dose

	Placebo (n=131)	Tegileridine 0.75 mg (n=132)	Tegileridine 1.0 mg (n=131)	Morphine (n=132)
**Subject satisfaction scores**				
n (n missing)	127 (4)	129 (3)	131 (0)	131 (1)
Median (IQR)	9.0 (8.0-10.0)	9.0 (8.0-10.0)	10.0 (8.0-10.0)	10.0 (9.0-10.0)
Z; *P*		0.13; *P* = 0.8938	2.25; *P* = 0.0247	2.86; *P* = 0.0042
**Investigator satisfaction scores**				
n (n missing)	127 (4)	129 (3)	131 (0)	132 (0)
Median (IQR)	8.0 (7.0-9.0)	9.0 (8.0-10.0)	9.0 (8.0-10.0)	9.0 (8.0-10.0)
Z; *P*		4.36; *P* <0.0001	4.04; *P* <0.0001	4.86; *P* <0.0001

IQR, interquartile range.

Wilcoxon rank sum test was used.

Table S7. Adverse event of special interest

	Placebo (n=131)				Tegileridine 0.75 mg (n=132)				Tegileridine 1.0 mg (n=131)				Morphine (n=132)			
	All	Severity			All	Severity			All	Severity			All	Severity		
		Mild	Moderate	Severe		Mild	Moderate	Severe		Mild	Moderate	Severe		Mild	Moderate	Severe
**Any,** n (%)	42 (32.1)	22 (16.8)	20 (15.3)	0	45 (34.1)	19 (14.4)	26 (19.7)	0	48 (36.6)	28 (21.4)	20 (15.3)	0	51 (38.6)	22 (16.7)	28 (21.2)	1 (0.8)
Vomiting	27 (20.6)	12 (9.2)	15 (11.5)	0	31 (23.5)	12 (9.1)	19 (14.4)	0	29 (22.1)	14 (10.7)	15 (11.5)	0	38 (28.8)	16 (12.1)	21 (15.9)	1 (0.8)
Nausea	30 (22.9)	19 (14.5)	11 (8.4)	0	29 (22.0)	13 (9.8)	16 (12.1)	0	25 (19.1)	16 (12.2)	9 (6.9)	0	32 (24.2)	20 (15.2)	12 (9.1)	0
Dizziness	0	0	0	0	2 (1.5)	1 (0.8)	1 (0.8)	0	3 (2.3)	3 (2.3)	0	0	1 (0.8)	1 (0.8)	0	0
Respiratory depression	0	0	0	0	1 (0.8)	1 (0.8)	0	0	0	0	0	0	0	0	0	0

Data were analyzed in all randomized subjects who received study treatments. There were no missing data.

Table S8. Change in respiratory rate from baseline

	Placebo (n=131)	Tegileridine 0.75 mg (n=132)	Tegileridine 1.0 mg (n=131)	Morphine (n=132)
1 h after treatment				
n (n missing)	127 (4)	125 (7)	128 (3)	130 (2)
Mean (SD)	0.5 (3.9)	0.6 (3.6)	0.0 (3.3)	0.8 (5.8)
2 h after treatment				
n (n missing)	125 (6)	125 (7)	128 (3)	130 (2)
Mean (SD)	0.4 (3.8)	-0.2 (4.2)	-0.1 (4.8)	0.8 (5.1)
3 h after treatment				
n (n missing)	125 (6)	124 (8)	128 (3)	130 (2)
Mean (SD)	0.7 (4.4)	0.7 (4.3)	0.5 (4.8)	1.4 (5.0)
6 h after treatment				
n (n missing)	125 (6)	124 (8)	127 (4)	129 (3)
Mean (SD)	1.1 (4.7)	1.1 (4.2)	1.2 (4.8)	2.2 (7.1)
12 h after treatment				
n (n missing)	122 (9)	124 (8)	127 (4)	129 (3)
Mean (SD)	1.1 (4.3)	1.8 (4.8)	1.8 (5.2)	1.6 (5.7)
24 h after treatment				
n (n missing)	122 (9)	124 (8)	127 (4)	129 (3)
Mean (SD)	2.2 (4.7)	2.5 (4.6)	2.4 (5.4)	2.3 (6.6)
At the end of study				
n (n missing)	125 (6)	125 (7)	127 (4)	130 (2)
Mean (SD)	1.5 (3.7)	1.9 (3.6)	1.3 (4.7)	2.1 (4.5)

Supplemental Methods S1

Supplemental Methods S1.1 In Vivo Studies

Analgesia *in vivo* studies were performed at Shanghai Institute of Materia Medica, Chinese Academy of Sciences (Shanghai, China), with Sprague-Dawley rats (male or female) housed in standard conditions with a 12-hour light/dark cycle. The studies were approved by the respective Institutional Animal Care and Use Committees.

For each study, rats (approximately 200 g) were randomly assigned to saline, tegileridine 0.1 mg/kg, tegileridine 0.3 mg/kg, tegileridine 1.0 mg/kg, and morphine 8.0 mg/kg treatment groups (10 rats for each group; male:female, 1:1). All compounds were administered subcutaneously in a volume of 1.0 mL/kg. Experimenter was blind to the treatment allocation during behavioral observations.

Data were analyzed using one-way analysis of variance, followed by pairwise comparisons between tegileridine and morphine versus saline at each time point using Tukey's posthoc multiple comparison test.

Supplemental Methods S1.2 Rat Photothermal Tail-Flick Study

Rats were restrained in specially designed restraint barrels, with only their tails exposed. Prior to the experiment, the rat tails were cleaned with 75% ethanol, and the distal third of the tail was stimulated by using SW-200 photothermal tail pain meter (Chengdu Taimeng Technology Co., Ltd., Chengdu, China). The latency of the rat to withdraw the tail from the stimulus was recorded as a pain threshold. After baseline testing, rats received a subcutaneous injection of saline, morphine, or test compound. At 5, 30, 60, and 120 minutes post-administration, rats were retested. A cutoff time of 20 seconds was used to prevent injury to the rat. The percent maximum possible antinociceptive effect (% maximum possible effect [MPE]) was determined using the formula:

$$\%MPE = (\text{Post drug latency} - \text{baseline latency}) / (20 - \text{baseline latency}) \times 100$$

Supplemental Methods S1.3 Rat Mechanical Tail-Pressure Study

Rats were restrained in specially designed restraint barrels, with only their tails exposed. Prior to the experiment, the rat tails were cleaned with 75% ethanol, and the distal third of the tail was stimulated by using using YLS-3E electronic pressure apparatus (Jinan Technology & Market Co., Ltd., Shandong, China). The pressure value at which the rat exhibited a tail withdrawal or a full-body flinch response was considered as the pain threshold. To prevent tissue damage, the pressure at which the pain threshold value doubles was set as the cut-off pressure. After baseline testing, rats received a subcutaneous injection of saline, morphine, or test compound. At 5, 30, 60, and 120 minutes post-administration, rats were retested. The %MPE was determined using the formula:

$$\%MPE = (\text{Post drug pain threshold} - \text{baseline pain threshold}) / \text{baseline pain threshold} \times 100$$

Supplemental Methods S1.4 Rat Incisional Pain Study

Prior to the incision surgery, rats were acclimated to the experimental apparatus once daily for at least two consecutive days. Thereafter, the pain threshold of the left hindlimb was tested once daily for two consecutive days, with the average value taken as the preoperative baseline pain threshold. An incision surgery was performed on the plantar aspect of the left hindlimb. Two or 3 days after surgery, rats received a subcutaneous injection of saline, morphine, or test compound. Tactile allodynia was measured at baseline, 5, 30, and 60 minutes after drug administration.

Supplemental Methods S1.5 Human study design considerations

The study was designed in accordance with pain-related clinical trial guidelines and referenced the APOLLO-2 study of oliceridine (an approved biased MOR ligand).

This study employed the numerical rating scale (NRS) scale for pain assessment and utilized morphine as the positive control, consistent with the APOLLO-2 study. Selection of the target population in this study was optimized based on the actual situation in China:

(1) The surgical types were not limited to abdominoplasty but extended to post-abdominal surgeries with a wide range of surgical types, enhancing adaptability.

(2) The target NRS score within 4 hours post-surgery was set at ≥4 (compared to 5 in the APOLLO-2 study).

(3) The inclusion and exclusion criteria were optimized, such as age and BMI.

Moreover, in line with the EMA guidelines, this study set the primary efficacy endpoint as summed pain intensity difference (SPID) at rest over the first 24 h from Time 0 ($SPID_{24}$), rather than response rate (refer to following note for defination). The two studies demonstrated the cumulative response to therapeutic interventions from different perspectives.

Note. Response rate in the APOLLO-2 study was defined as the proportion of patients who responded to study medication compared to placebo at the end of the randomized 24-hour treatment period: (1) at least a 30% improvement in time-weighted (SPID) from baseline at 24 hours, (2) no use of protocol-specified rescue pain medicine, (3) no early discontinuation of study medication for any reason, and (4) did not reach protocol-specified study medication dosing limit.

Supplemental Methods S1.6 Human study rationale for dose selection

The doses of study medications in this study were selected based on the dose equivalence relationship studies and prior clinical studies:

(1) Preclinical studies demonstrated that tegileridine exhibited dose-dependent analgesic effect in rat pain models (including postoperative pain, thermal pain, mechanical pain, and chemical pain), and its equieffective dose ratio to morphine was approximately 1:8 to 1:9.

(2) Based on the results from the single ascending dose phase 1 study of tegileridine, modeling integrating the relationship between in vivo exposure and adverse events determined the recommended doses for the abdominal postoperative analgesia phase 2 study were a loading dose of 0.5, 0.75, or 1.0 mg with a 0.05-mg PCA pump dose of tegileridine, compared with placebo and a loading dose of 3 mg with a 1-mg PCA pump dose of morphine. This regimen was compared against morphine (loading dose: 3 mg + PCA bolus: 1 mg). According to the efficacy and safety outcomes of the phase 2 study, this phase 3 study selected the 0.75 mg and 1.0 mg loading doses of tegileridine for validation versus the 3 mg morphine loading dose.
