# Supplementary material for: Tegileridine for moderate-to-severe acute pain following abdominal surgery: A randomized, double-blind, phase 3 clinical trial
Source: Cell Rep Med. 2025 Dec 8;6(12):102477. doi: 10.1016/j.xcrm.2025.102477 (PMC12765830; doi:10.1016/j.xcrm.2025.102477)
Supplement: Data S1. Clinical trial-related documents — Data S1 include the protocol and statistical analysis plan. This information is related to STAR Methods. [file mmc2.pdf]

The Supplement contains the following items:

1. Final protocol (V3.0) and amendments

2. Final Statistical analysis plan (V1.0)

EFFICACY AND SAFETY OF SHR8554 INJECTION FOR POSTOPERATIVE ANALGESIA AFTER ABDOMINAL SURGERY: A MULTICENTER, RANDOMIZED, DOUBLE-BLIND, PLACEBO/ACTIVE-CONTROLLED PHASE 3 CLINICAL TRIAL

Study protocol

**Protocol No.:**	SHR8554-301
**Drug No.:**	SHR8554
**Drug Name:**	SHR8554 Injection
**Study Director:**	Yong Cao
**Clinical Study Leading Site:**	Union Hospital, Tongji Medical College, Huazhong University of Science and Technology
**Principal Investigator:**	Professor Xiangdong Chen
**Version Number:**	3.0
**Version Date:**	13 April 2021
**Sponsor:**	Jiangsu Hengrui Pharmaceuticals Co., Ltd.
	No. 7 Kunlunshan Road, Economic and Technological Development Area, Lianyungang, Jiangsu Province, 222047

Confidentiality Statement

The information contained in this protocol is confidential and is only intended for use by clinical investigators and shall not be disclosed unless required by current laws or regulations. Its copyright is the property of Jiangsu Hengrui Pharmaceuticals Co., Ltd. or its subsidiaries, and must not be copied or distributed to any person who has not participated in this clinical study, except for those who have signed a confidentiality agreement with Jiangsu Hengrui Pharmaceuticals Co., Ltd. or its subsidiaries.

VERSION HISTORY/REVISION HISTORY

Documents	Version No.	Version Date	Reason for revision and summary of changes
Initial Version	1.0	20 Nov 2020	Not applicable
Previous version	2.0	20 Dec 2020	**Revised in accordance with ethical comments:**<br>1. The description of dosing regimen was revised to:<br>Phase 2b study:<br>SHR8554 Injection 0.75 mg group: the loading dose is 0.75 mg (5 mL), and the single infusion dose by PCA pump is 0.05 mg (1 mL).<br>SHR8554 Injection 1.0 mg group: the loading dose is 1.0 mg (5 mL), and the single infusion dose by PCA pump is 0.05 mg (1 mL).<br>Morphine hydrochloride injection group: the loading dose is 3.0 mg (5 mL) and the single infusion dose by PCA pump is 1.0 mg (1 mL).<br>Placebo (saline, i.e., 0.9% sodium chloride injection) group: the loading dose is 5 mL of normal saline and the single infsion dose by the PCA pump is 1 mL of normal saline.<br>2. The selection of control drug was revised to:<br>In 2007, the Center for Drug Evaluation (CDE) of the National Medical Products Administration issued "Key Points to Consider in Clinical Trials for Analgesic Drug Registration in China", which states: "Randomized controlled studies comparing with placebo are required". A placebo control will be used to investigate the true adverse events of the investigational product and to compare the efficacy of the investigational product with that of placebo. The analgesic effect of morphine is clear, so it is used as a positive control to compare with the safety and efficacy of the investigational drug.<br>3. The version times were added to the regulations:<br>Revised into:

			The study is designed based on the following laws and regulations:<br>1) *Drug Registration Regulation* (2020 Version)<br>2) *Good Clinical Practice (GCP)* (2020 Version)<br>3) *Technical Guidelines for Clinical Pharmacokinetic Studies of Chemical Drugs* (2019 Version)<br>4. In the informed consent process and records, "subjects will be required to read and review the ICF approved by the Ethics Committee", where "read and review" was revised into "read and understand".<br>5. In the step of withdrawal from the study, "if the subject refuses to come to the study site for further visits, his/her survival status should still be followed up", was revised into "if the subject refuses to come to the study site for further visits, his/her adverse events and outcomes should still be followed up".<br>6. Description of emergency unblinding was revised into:<br>If the treatment grouping information must be unblinded during the study due to urgent management of SAE, or to decide the subsequent treatment, the site should promptly notify the sponsor of the reasons for unblinding, and the principal investigator should submit an application for unblinding.<br>**Additional supplements:**<br>1. Added the principal investigator's institution and its responsible person (printed), and the statistical institution and its responsible person (printed) on the signature page of the protocol.
Current version	3.0	13 April 2021	**Revised according to CDE review comments:**<br>1. The study phase was revised to Phase 3, and the relevant design of Phase 2b was deleted;<br>2. The sample size was revised to 528 subjects, and the statistical hypothesis and judgement rules were modified;<br>**Other amendments:**<br>1. The screening period was revised into: from

			signing the ICF to randomization, with a maximum of no more than 7 days, and the day of randomization will be recorded as D1;<br>2. Addition of study procedures: NRS pain intensity score and pain relief score may not be rated if the subject is sleeping at the corresponding assessment point;<br>3. In the list of prohibited drugs, the hepatic drugs of enzyme inhibitors/inducers were revised into CYP2D6, CYP3A4 and CYP3A5 inhibitors/inducers, and the corresponding hepatic drugs of enzyme inhibitors/inducers in the exclusion criteria were also revised;<br>4. In Exclusion Criteria 2. General examination/laboratory tests, "excluding abnormalities during anesthesia and surgery" was specified as "excluding abnormalities from entering the operation room to postoperative extubation of endotracheal intubation";<br>5. Other errata.

Sponsor Signature Page

We have read and confirmed this clinical study protocol (Protocol No.: SHR8554-301, Version No.: 3.0, Date: 13 April 2021). I agree to perform the relevant responsibilities in accordance with Chinese laws, *the Declaration of Helsinki*, China GCP and this protocol.

Sponsor: Jiangsu Hengrui Pharmaceuticals Co., Ltd.

Yong Cao		
Study Director (Printed)	Study Director (Signature)	Signature Date (DD/MM/YYYY)

Principal Investigator Signature Page (Leading Site)

Protocol Title: Efficacy and Safety of SHR8554 Injection for Postoperative Analgesia after Abdominal Surgery: A Multicenter, Randomized, Double-Blind, Placebo/Active-Controlled Phase 3 Clinical Trial

Protocol No.: SHR8554-301.

Version number and date: 3.0, 13 April 2021.

By signing this protocol signature page, the investigator confirms and agrees that:

I have read the above protocol and its appendixes.

I have fully discussed the contents of this protocol with the sponsor, Jiangsu Hengrui Pharmaceuticals Co., Ltd.

I agree to conduct the study in accordance with this protocol and to perform relevant responsibilities in accordance with China GCP, local regulations and other applicable regulations.

This document contains confidential information and shall not be disclosed without written authorization from the sponsor, except for those directly involved in the conduct of the study or in the ethical/regulatory review.

I agree that all staff involved in this study will be informed of their obligations in fulfilling these commitments.

Study Site: Union Hospital, Tongji Medical College, Huazhong University of Science and Technology

Xiangdong Chen		
Principal Investigator (Printed)	Principal Investigator (Signature)	Signature Date (DD/MM/YYYY)

Principal Investigator Signature Page (Study Site)

Protocol Title: Efficacy and Safety of SHR8554 Injection for Postoperative Analgesia after Abdominal Surgery: A Multicenter, Randomized, Double-Blind, Placebo/Active-Controlled Phase 3 Clinical Trial

Protocol No.: SHR8554-301.

Version number and date: 3.0, 13 April 2021.

By signing this protocol signature page, the investigator confirms and agrees that:

I have read the above protocol and its appendixes.

I have fully discussed the contents of this protocol with the sponsor, Jiangsu Hengrui Pharmaceuticals Co., Ltd.

I agree to conduct the study in accordance with this protocol and to perform relevant responsibilities in accordance with China GCP, local regulations and other applicable regulations.

This document contains confidential information and shall not be disclosed without written authorization from the sponsor, except for those directly involved in the conduct of the study or in the ethical/regulatory review.

I agree that all staff involved in this study will be informed of their obligations in fulfilling these commitments.

Study Site:____

____________________	____________________	____________________
Principal Investigator (Printed)	Principal Investigator (Signature)	Signature Date (DD/MM/YYYY)

Signature Page of Statistical Institution

We have read and confirmed this clinical study protocol (Protocol No.: SHR8554-301, Version No.: 3.0, Date: 13 April 2021). I agree to perform the relevant responsibilities in accordance with Chinese laws, *the Declaration of Helsinki*, China GCP and this protocol.

Statistical Institution: Department of Biostatistics, School of Public Health, Nanjing Medical University

Hao Yu		
Responsible Person (Printed)	Responsible Person (Signature)	Signature Date (DD/MM/YYYY)

Table of Contents

Table of Contents 1
Synopsis 1
Clinical Study Flow Chart 10
Abbreviations 15
1. Introduction: Study Background and Scientific Rationale 16
1.1. Study Background 16
1.2. Scientific Rationale for Study Design 24
2. Study Objective and Efficacy Variables 26
2.1. Study Objective 26
2.2. Efficacy Variables 26
3. Study Design 26
4. Subject Selection and Withdrawal 29
4.1. Inclusion Criteria 29
4.2. Exclusion Criteria 29
5. Investigational Products 31
5.1. Overview of Investigational Products 31
5.2. Randomization and Blinding 32
5.3. Emergency Unblinding 32
5.4. Project Unblinding 33
5.5. Treatments 33
5.6. Rescue Treatments 33
5.7. Concomitant Medications 34
6. Study Procedures 35
6.1. Screening Period: From Signing the ICF to Randomization, With a Maximum of No More Than 7 Days, and the Day of Randomization Will Be Recorded as D1 35
6.2. Treatment Period: From Randomization to 24 h, With the Time to Start Infusion of the Loading Dose of Investigational Product Recorded as 0 h. 36
6.3. Follow-up Period: 24 h to D4 ± 1 38
6.4. Unscheduled Visits 38
6.5. Withdrawal by the Subject 38
6.6. Procedures for Subject Withdrawal from the Study 39

6.7. Premature Termination or Suspension of the Study 39
6.8. End of Study Definition 39
7. Evaluation 39
7.1. Efficacy Evaluation 39
7.2. Safety Evaluation 40
7.3. PK Evaluation 42
8. Adverse Events Reporting 43
8.1. Adverse Events (AEs) 43
8.2. Serious Adverse Events (SAEs) 44
8.3. Pregnancy 45
8.4. Reporting Procedures 46
9. Clinical Monitoring 47
10. Data Analysis/Statistical Method 47
10.1. Sample Size 47
10.2. Statistical Analysis Plan 47
10.3. Statistical Hypotheses and Discriminant Rules 48
10.4. Analysis Populations 48
10.5. Statistical Methods 48
11. Data Management Methods 50
11.1. Data Collection 50
11.2. Data Management 50
12. Source Data and Source Documents 51
13. Quality Assurance and Quality Control 52
14. Regulations, Ethics, Informed Consent and Subject Protection 52
14.1. Regulatory Considerations 52
14.2. Ethics 53
14.3. Ethics Committee 53
14.4. Informed Consent 53
14.5. Confidentiality of Subject Information 54
15. Publication of Study Results 54
16. References 55
17. Appendix 56
Appendix 1. NRS Pain Score at Rest 56

Appendix 2. Subject Satisfaction Score 57
Appendix 3. Investigator Satisfaction Score 58
Appendix 4. Subject Pain Relief Score (Likert Scale) 59
Appendix 5. ASA Score 60
Appendix 6. Fridericia's Formula 61

表目录

Table 5-1 Prohibited Medications 34

Table 7-1 Laboratory Tests 41

Table 8-1 Principles of AE/SAE/Pregnancy Collection and Follow-up Period 47

Synopsis

**Study Title**	Efficacy and Safety of SHR8554 Injection for Postoperative Analgesia after Abdominal Surgery: a Multicenter, Randomized, Double-Blind, Placebo/Active-Controlled Phase 3 Clinical Trial
**Protocol No.**	SHR8554-301
**Version Number and Date**	3.0, 13 April 2021
**Study Phase**	Phase 3
**Investigational Products**	1. SHR8554 Injection: 1 mL: 1 mg, 5 mL: 5 mg, manufactured and provided by Jiangsu Hengrui Pharmaceuticals Co., Ltd.;<br>2. Morphine hydrochloride injection: 1 mL: 10 mg, manufactured by Shenyang First Pharmaceutical Co., Ltd., Northeast Pharmaceutical Group, purchased and provided by Jiangsu Hengrui Pharmaceuticals Co., Ltd.<br>3. Placebo: 0.9% sodium chloride solution, strength: 5 mL, manufactured and provided by Jiangsu Hengrui Pharmaceuticals Co., Ltd.
**Sponsor**	Jiangsu Hengrui Pharmaceuticals Co., Ltd.
**Principal Investigator**	Xiangdong Chen
**Study Objective**	To evaluate the efficacy and safety of SHR8554 Injection for analgesia after abdominal surgery.
**Study Subjects**	Subjects who will undergo selective abdominal surgery (laparotomy or laparoscopy) under general anesthesia and have an NRS ≥ 4 points within 4 h after the surgery.

**Study Design**	The study will use a multicenter, randomized, double-blind, placebo/active-controlled trial design.<br>The study process consists of a screening period, a treatment period, and a follow-up period.<br>**Screening period: from signing the ICF to randomization, with a maximum of no more than 7 days, and the day of randomization will be recorded as D1.**<br>The subject will sign the informed consent form and complete the screening according to the study flow chart. The subject will undergo abdominal surgery (laparotomy or laparoscopy) under general anesthesia (total intravenous anesthesia or intravenous-inhalation combined anesthesia). Sevoflurane is selected as inhalation anesthetics, propofol injection is selected as intravenous anesthetics, and the dose of general anesthetics will be determined on an individual basis. Sufentanil injection (0.2-0.6 μg/kg) is selected as opioid analgesics during the anesthetic induction period. Remifentanil injection (0.1-0.5 μg/kg/min) is selected as opioid analgesics during the anesthetic maintenance period, sufentanil injection (0.1-0.2 μg/kg/time) can be added, and the doses can be adjusted appropriately according to the needs of surgery and the subject's individual conditions. The type and dose of muscle relaxants and other anesthetic adjuvant drugs are at the discretion of the anesthesiologist and/or surgeon. 2.5 μg of sufentanil will be intravenously injected immediately (± 5 min) after the surgery (the last suture is completed) to prevent postoperative breakthrough pain.<br>Within 4 hours after the surgery, the investigator will instruct the subjects to assess the pain intensity (PI) at rest using the Numeric Rating Scale (NRS, 0-10 for varying degrees of pain, 0 for painlessness, larger numbers for more severe pain, and 10 for the worst pain), and it is recommended that the interval between the two assessments should not exceed 30 minutes. The subjects with NRS score ≥ 4 at rest at any time within 4 hours after the surgery can be randomized (it is recommended to be randomized within 10 min after the NRS score reaches 4 or more for the first time). The subjects will be randomized in a 1:1:1:1 ratio to one of the following groups: SHR8554 Injection 0.75 mg group, SHR8554 Injection 1.0 mg group, morphine hydrochloride injection group, and placebo group.<br>**Treatment period: from randomization to 24 h, with the time to start infusion of the loading dose of investigational product recorded as 0 h.**<br>After randomization, the investigator should infuse the investigational product to the subject as soon as possible (within 15 min after the randomization). The loading dose of the investigational product will be 5 mL, administered by an infusion pump, and the intravenous infusion time will be approximately 10 min. The Patient

	Controlled Analgesia (PCA) pump containing the investigational product can be initiated approximately 30 min after the start of infusion of the loading dose. The subject can receive 1 mL of the investigational product at a single press of PCA pump, and the minimum interval between two adjacent effective presses is 10 minutes. The subject will continue using the PCA pump until 24 h (± 5 min).<br>After the end of of loading dose infusion, rescue analgesia may be given if the investigator determines that the investigational product can not meet the subject's analgesic needs (NRS ≥ 4 points). The first choice of rescue analgesics is parecoxib sodium for injection (20 mg for the first intravenous injection, followed by additional doses as needed, with a total dose of not more than 80 mg over 24 h), and sufentanil injection (2.5 μg for a single intravenous injection) can be used after reaching the maximum cumulative dose of parecoxib sodium for injection.<br>After randomization, the investigator will instruct the subject to assess the NRS score at rest within 5 minutes prior to the start of infusion of loading dose as baseline. The investigator will instruct the subject to assess the NRS scores at rest immediately after the end of loading dose infusion and 20 min, 30 min, 45 min (± 1 min as the assessment point window from immediately after the end of loading dose infusion to 45 min), 1 h, 1.5 h, 2 h, 3 h, 4 h, 5 h, 6 h (± 5 min as the assessment point window from 1 h to 6 h), 8 h, 10 h, 12 h (± 10 min as the assessment point window for 8 h, 10 h, and 12 h), 18 h, and 24 h (± 20 min as the assessment point window for 18 h and 24 h). At the same time as each assessment of NRS score, the investigator will instruct the subject to assess the degree of pain relief (PR) using the Likert scale (0 as no response, 1 mild response, 2 moderate response, 3 significant response, 4 complete response).<br>PK blood sampling: three PK blood samples will be collected from each subject for plasma concentration analysis and drug metabolizing enzyme gene analysis as specified in the *Clinical Sample Operation Manual*. The PK blood sampling time points include: immediately after the end of the infusion of loading dose of investigational product, and 20 min, 45 min, 1 h, 3 h, 6 h, 12 h and 24 h after the infusion (see *Clinical Sample Operation Manual* for the specific blood sampling points for each subject).<br>Blood samples at each time point can be collected at any time within the time window (see the *Clinical Sample Operation Manual* for the time window of blood sample collection), and the time of blood collection should be accurately recorded. If the PK sampling time coincides with the pain scoring time, the pain scoring should be performed first. Approximately 3 mL of venous blood will be collected

	at each collection point into K_2EDTA tubes, pretreated and preserved as required until use for analysis.<br>The subject will be required to have continuous ECG monitoring (brief interruptions are allowed due to transit and the subject's necessary activities) from 0 h to stopping the infusion of investigational product.<br>The investigator should perform 12-lead ECG and record the subject's vital signs (blood pressure [BP], respiratory rate [RR], heart rate [HR], body temperature [T]) and transdermal oxygen saturation (SpO_2) from randomization to 0 h, and at 1 h, 2 h, 3 h, 6 h (± 5 min as the assessment point window from 1 h to 6 h), 12 h and 24 h (± 15 min as the assessment point window for 12 h and 24 h). The investigator will decide whether additional 12-lead ECG is required according to the subject's clinical condition.<br>During the treatment period, the time to the first use of rescue analgesics, the cumulative amount of rescue analgesics from the end of the infusion of loading dose to 24 h, the number of treatments of rescue analgesics, the total number of PCA pump presses within 24 h, the number of effective presses of PCA pump, and the time of effective presses of PCA pump will be recorded. Within 30 min after stopping the PCA pump at 24 h (± 5 min), the subject and the investigator will score their satisfaction with analgesia of the investigational product, respectively.<br>**Follow-up Period: 24 h to D4 ± 1**<br>During the follow-up period, the subject will complete relevant examinations and complete the safety assessment according to the study flow chart.
**Dosage Regimen**	SHR8554 Injection 0.75 mg group: the loading dose is 0.75 mg (5 mL), and the single infusion dose by PCA pump is 0.05 mg (1 mL).<br>SHR8554 Injection 1.0 mg group: the loading dose is 1.0 mg (5 mL), and the single infusion dose by PCA pump is 0.05 mg (1 mL).<br>Morphine hydrochloride injection group: the loading dose is 3.0 mg (5 mL) and the single infusion dose by PCA pump is 1.0 mg (1 mL).<br>Placebo (saline, i.e., 0.9% sodium chloride injection) group: the loading dose is 5 mL of normal saline and the single infusion dose by the PCA pump is 1 mL of normal saline.
**Rescue Analgesia**	After the end of of loading dose infusion of the investigational product, parecoxib sodium for injection will be first used as a rescue analgesic if the investigator determines that the investigational product can not meet the subject's analgesic needs (NRS ≥ 4 points). Dose of parecoxib sodium for injection: 20 mg will be injected intravenously for the first time, followed by additional doses as needed,

	with a total dose of not more than 80 mg over 24 h. Sufentanil injection (2.5 μg for a single intravenous injection) can be used after reaching the maximum cumulative dose of parecoxib sodium for injection. Rescue analgesics should be truthfully documented in the original records and eCRFs. After the treatment period, the subject's subsequent analgesics and dosages will be at the discretion of the investigator according to the hospital's routine practices.
**Antiemetic Therapy**	From the end of surgery to the end of the follow-up period, the investigator may first use tropisetron hydrochloride injection for antiemetic treatment according to the condition of nausea and vomiting, with the first intravenous injection of 2.5 mg, followed by additional doses as needed, with a total dose of not more than 10 mg over 24 h. If antiemetic therapy is still required after reaching the maximum dose of tropisetron hydrochloride injection, the investigator may use other antiemetic agents. Prophylactic antiemetic therapy is not allowed.
**Inclusion Criteria**	Subjects will be eligible for enrollment into the study if all of the following criteria are met:<br>1) Before starting any trial-related activity, the subject must voluntarily sign the ICF, fully understand the purpose and significance of this trial, and willingly comply with the study procedures;<br>2) Subjects who will undergo selective abdominal surgery (laparotomy or laparoscopy) under general anesthesia for ≥ 1 h;<br>3) 18 to 65 years of age, male or female;<br>4) 18.0 kg/m^2 ≤ body mass index (BMI) ≤ 28.0 kg/m^2;<br>5) ASA I-II;<br>6) NRS ≥ 4 at rest at any time within 4 h after the surgery.
**Exclusion Criteria**	Subjects will be ineligible for enrollment into the study if any of the following criteria is met:<br>1. Disease conditions<br>1) Have a prior history of vestibular dysfunction or have dizziness, nausea, retching or vomiting within 1 week prior to screening;<br>2) Have a prior history of ischemic stroke or transient ischemic attack (TIA);<br>3) Have a prior history of difficult airway, such as obstructive sleep apnea syndrome;<br>4) Have prior reflux esophagitis;<br>5) Have a history of myocardial infarction or unstable angina pectoris, or severe arrhythmia such as atrioventricular block grade II or higher, or NYHA

	functional class II or higher within 6 months prior to screening;<br>6) Have a prior history of psychiatric disorders (e.g., schizophrenia, depression, etc.), cognitive dysfunction, epilepsy, or Parkinson's syndrome;<br>7) Have advanced malignancies or malignant tumors with extensive metastases;<br>2. General examination/laboratory tests<br>1) Subjects who do not receive formal antihypertensive therapy or have inadequate blood pressure control (sitting systolic blood pressure ≥ 160 mmHg or ≤ 90 mmHg at screening, and/or diastolic blood pressure ≥ 100 mmHg or ≤ 60 mmHg at screening, excluding abnormalities from entering the operation room to postoperative extubation of endotracheal intubation);<br>2) QTc at screening: > 450 ms for males and > 470 ms for females (QTc calculated by Fridericia's formula);<br>3) Transdermal oxygen saturation (SpO_2) < 90% at screening (excluding abnormalities from entering the operation room to postoperative extubation of endotracheal intubation);<br>4) Poor glycemic control at screening: random blood glucose ≥ 11.1 mmol/L;<br>5) Abnormal liver function at screening: aspartate aminotransferase (AST) and/or alanine aminotransferase (ALT) ≥ 1.5 × ULN and/or total bilirubin (TBIL) ≥ 1.5 × ULN;<br>6) Abnormal renal function at screening: serum creatinine (Cr) ≥ 1.5 × ULN;<br>7) Coagulation abnormalities at screening: prolonged prothrombin time (PT) exceeding the upper limit of normal by 3 seconds and/or prolonged activated partial thromboplastin time (APTT) exceeding the upper limit of normal by 10 seconds;<br>8) Have positive infectious disease screening for hepatitis B surface antigen (HBsAg), hepatitis C antibody (HCVAb), treponema pallidum antibody or human immunodeficiency virus (HIV) antibody at screening.<br>3. Drug use<br>1) Known allergies to opioids and other drugs that may be used during the trial, such as nonsteroidal anti-inflammatory drugs, antiemetics, etc.;<br>2) Consecutive use of opioid analgesics for more than 10 days for any reason within 3 months prior to randomization, or the time of last dose is less than 5 half-lives from randomization, including but not limited to the following drugs with analgesic effect: selective α_2-adrenoceptor agonists, opioid agonists/antagonists, nonsteroidal anti-inflammatory drugs, sedatives, monoamine oxidase inhibitors, glucocorticoids, CYP2D6, CYP3A4 and CYP3A5 inhibitors/inducers, antihistamines, antiepileptics, anxiolytics,

	antidepressants, etc. Refer to the Table of Prohibited Drugs for specific drugs.<br>4. Others<br>1) Have a history of drug abuse and/or alcohol abuse within 1 year prior to screening, i.e., more than 15 g of alcohol per day on average (15 g alcohol is equivalent to 450 mL of beer or 150 mL of wine or 50 mL of low-alcoholic liquor);<br>2) Positive drug abuse screening result at screening;<br>3) Pregnant or lactating women;<br>4) Subjects who have a birth plan, are unwilling or unable to take effective contraceptive measures from 30 days before screening to half a year after the end of the trial;<br>5) Subjects who participated in other drug clinical trials (defined as receiving investigational product or placebo) within 3 months prior to screening;<br>6) Other conditions that in the opinion of the investigator make the subject unsuitable to participate in this clinical trial.
**Withdrawal criteria**	1. Subjects may withdraw from the study at any time.<br>2. The investigator may decide to discontinue the investigational product and/or withdraw the subject from the study if any medical condition occurs prior to dosing or during the administration, which may put the subject at risk, or if the subject is unable to complete the study as specified in the protocol.
**Efficacy Assessments**	**Primary Efficacy Variable**<br>Time-weighted Sum of Pain Intensity Differences at rest up to 24 h after starting infusion of loading dose of investigational product ($SPID_{24}$).<br>**Secondary Efficacy Variables**<br>1) Time-weighted Sum of Pain Intensity Differences at rest at 6 h, 12 h, 18 h, and 12-24 h ($SPID_6$, $SPID_{12}$, $SPID_{18}$, $SPID_{12-24}$);<br>2) Time-weighted Total Pain Relief at 6 h, 12 h, 18 h, 24 h, and 12-24 h ($TOTPAR_6$, $TOTPAR_{12}$, $TOTPAR_{18}$, $TOTPAR_{24}$, $TOTPAR_{12-24}$);<br>3) Time to the first use of rescue analgesics;<br>4) Cumulative amount of rescue analgesics from 0 h to 24 h;<br>5) Number of treatments of rescue analgesia from 0 h to 24 h;<br>6) Total number of presses and number of effective presses of PCA pump from 0 h to 24 h;<br>7) Percentage of subjects who do not use rescue analgesics from 0 h to 24 h;<br>8) The subject's satisfaction score with analgesic treatment;<br>9) The investigator's satisfaction score with analgesic treatment.

**Safety Assessments**	Safety assessments will include on-study AEs, laboratory tests (hematology, blood chemistry, urinalysis, coagulation), 12-lead ECG, vital signs (blood pressure [BP], respiratory rate [RR], heart rate [HR], body temperature [T]), transdermal oxygen saturation (SpO_2), and complete physical examination.<br>The following AEs need to be focused on and described statistically separately: nausea, vomiting, dizziness, and respiratory depression (respiratory depression is defined as respiratory rate < 8 beats/min and/or oxygen saturation < 90%).
**PK Assessments**	PPK model parameters CL, V, etc.
**Sample Size**	With reference to the results of the foreign study TRV130-3002, assuming that the primary efficacy variable $SPID_{24}$ in the SHR8554 0.75 mg group in this study is comparable to the efficacy in the 0.35 mg group in the TRV130-3002 study, and conservatively assuming that the mean (standard deviation) of $SPID_{24}$ in the low dose group of this study is 88 (45), and the mean (standard deviation) of $SPID_{24}$ in the placebo group is 70 (40), it's estimated to need 118 subjects in each group by the sample size calculation formula (PASS) of comparing the means of two samples to achieve the power of 90% with $\alpha = 0.05$ (two-sided), provided that the four groups are designed in a 1:1:1:1 ratio. Considering the dropout rate of 10%, each group is planned to enroll 132 subjects, with a total of 528 subjects.
**Data Analysis / Statistical Methods**	The general principles for descriptive statistical analysis in this study are as follows: Count data will be summarized using frequencies and percentages. Measurement data will be summarized using mean, standard deviation, median, maximum, minimum, etc. Plasma concentration data will be summarized using mean or geometric mean, standard deviation, coefficient of variation or geometric coefficient of variation, median, maximum, and minimum. SAS 9.4 will be used for calculation, and all statistical tests will be performed using two-sided tests, with $P \leq 0.05$ considered as statistically significant.<br>**Efficacy Analysis**<br>The primary efficacy variable $SPID_{24}$ will be compared between groups using analysis of variance, and $SPID_{24}$ in each group will be statistically described. The high dose group and low dose group will be tested sequentially, and descriptive statistics will be performed in the morphine group. For the secondary efficacy variables, analysis of variance, chi-square test, Fisher's exact probability method, and nonparametric test will be used for difference analysis according to the nature of the data.<br>**Safety Analysis**

	The incidence of AEs and ADRs in each group will be compared using chi-square test or Fisher's exact probability method, and frequency and percentage will be used to describe the AEs and ADRs.<br>**PK Analysis**<br>The population PK model parameters (CL, V, etc.) will be estimated using the pharmacometric method.

Clinical Study Flow Chart

Study Periods	Screening Period	Treatment Period (from randomization to 24 h, with the time to start infusion of the loading dose of investigational product recorded as 0 h)																		Follow-up Period
Visit (Day)	From signing the ICF to randomization, with a maximum of no more than 7 days, and the day of randomization will be recorded as D1	Before starting infusion of the loading dose of investigational product	Start infusion of the loading dose of investigational product																	24 h ~ D4±1
Time			0 h	Immediately after the infusion	20 min	30 min	45 min	1 h	1.5 h	2 h	3 h	4 h	5 h	6 h	8 h	10 h	12 h	18 h	24 h	
Signing the ICF	X																			
Eligibility criteria review	X																			
Medical histories (1)	X																			
Demographics	X																			
Complete physical examination (2)	X																			X
ECG monitoring (3)			X																	
Vital signs (4)	X	X						X		X	X			X			X		X	X
Oxygen saturation (SpO_2) (5)	X	X						X		X	X			X			X		X	X
12-Lead ECG (6)	X	X						X		X	X			X			X		X	X
Drug abuse screening	X																			

**Study Periods**	**Screening Period**	**Treatment Period (from randomization to 24 h, with the time to start infusion of the loading dose of investigational product recorded as 0 h)**																		**Follow-up Period**
**Visit (Day)**	**From signing the ICF to randomization, with a maximum of no more than 7 days, and the day of randomization will be recorded as D1**	**Before starting infusion of the loading dose of investigational product**	**Start infusion of the loading dose of investigational product**																	**24 h ~ D4±1**
**Time**			0 h	Immediately after the infusion	20 min	30 min	45 min	1 h	1.5 h	2 h	3 h	4 h	5 h	6 h	8 h	10 h	12 h	18 h	24 h	
(7)																				
Pregnancy test	X																			
Infectious disease screening (8)	X																			
Laboratory test (9)	X																			X
Pain intensity score (10)		X		X	X	X	X	X	X	X	X	X	X	X	X	X	X	X	X	
Pain relief score (11)				X	X	X	X	X	X	X	X	X	X	X	X	X	X	X	X	
Subject satisfaction score (12)																				X
Study doctor satisfaction score (13)																				X
Randomization	X																			
Administration of investigational			X																	

<table>
<tr><th>Study Periods</th><th>Screening Period</th><th colspan="17">Treatment Period (from randomization to 24 h, with the time to start infusion of the loading dose of investigational product recorded as 0 h)</th><th>Follow-up Period</th></tr>
<tr><th rowspan="2">Visit (Day)</th><th rowspan="2">From signing the ICF to randomization, with a maximum of no more than 7 days, and the day of randomization will be recorded as D1</th><th>Before starting infusion of the loading dose of investigational product</th><th colspan="16">Start infusion of the loading dose of investigational product</th><th>24 h ~ D4±1</th></tr>
<tr><th>Time</th><th>0 h</th><th>Immediately after the infusion</th><th>20 min</th><th>30 min</th><th>45 min</th><th>1 h</th><th>1.5 h</th><th>2 h</th><th>3 h</th><th>4 h</th><th>5 h</th><th>6 h</th><th>8 h</th><th>10 h</th><th>12 h</th><th>18 h</th><th>24 h</th><th></th></tr>
<tr><td>product</td><td></td><td></td><td colspan="17"></td><td></td></tr>
<tr><td>PK blood sampling (14)</td><td></td><td></td><td></td><td colspan="16">X</td><td></td></tr>
<tr><td>Recording the amount of rescue analgesics</td><td></td><td></td><td></td><td colspan="16">X</td><td></td></tr>
<tr><td>Recording the amount of antiemetic drug</td><td colspan="20">X</td></tr>
<tr><td>Concomitant medications (15)</td><td colspan="20">X</td></tr>
<tr><td>AEs (16)</td><td colspan="20">X</td></tr>
</table>

Notes:

Each test and procedure will be performed according to the flow chart. At screening, laboratory test results and electrocardiograms within 7 days prior to randomization will be acceptable, and infectious disease screening results within 30 days prior to randomization will be acceptable.

1) Medical histories: including past medical histories and treatments, medication before screening, recent participation in drug or medical device trials, smoking and alcohol consumption history, allergy history, etc.;
2) Complete physical examination: including overall appearance, skin and mucosa, lymph nodes, head and neck, chest, abdomen, muscles and bones, nervous system, and other parts of the body;

3) ECG monitoring: the subject will be required to have continuous ECG monitoring (brief interruptions are allowed due to transit and the subject's necessary activities) from 0 h to stopping the infusion of investigational product;
4) Vital signs: include blood pressure [BP], respiratory rate [RR], heart rate [HR], and body temperature [T]. At screening, vital signs should be measured before entering the operation room; during the treatment period: from randomization to 0 h, and at 1 h, 2 h, 3 h, 6 h (± 5 min as the assessment point window from 1 h to 6 h), 12 h and 24 h (± 15 min as the assessment point window for 12 h and 24 h assessments), vital signs should be recorded once by the investigator; and once during the follow-up period;
5) Oxygen saturation (SpO_2): at screening: the measurement time of SpO_2 should be before entering the operation room; during the treatment period: from randomization to 0 h, and at 1 h, 2 h, 3 h, 6 h (± 5 min as the assessment point window from 1 h to 6 h), 12 h and 24 h (± 15 min as the assessment point window for 12 h and 24 h assessments), the investigator should record SpO_2 once; and once during the follow-up period;
6) 12-lead ECG: at screening: the examination time should be before the operation; during the treatment period: from randomization to 0 h, and at 1 h, 2 h, 3 h, 6 h (± 5 min as the assessment point window from 1 h to 6 h), 12 h and 24 h (± 15 min as the assessment point window for 12 h and 24 h assessments), the investigator should perform a 12-lead ECG for the subject. The investigator will decide whether additional 12-lead ECG is required according to the subject's clinical condition; it will be measured once during the follow-up period;
7) Drug abuse screening: including methamphetamine, dimethyldioxyamphetamine, tetrahydrocannabinol acid, ketamine, and morphine;
8) Infectious disease screening: including hepatitis B surface antigen, hepatitis C antibody, treponema pallidum antibody, HIV antibody;
9) Laboratory tests: including hematology: red blood cells (RBC), white blood cells (WBC), neutrophils (NEUT), hemoglobin (Hb), and platelets (PLT); blood chemistry: aspartate aminotransferase (AST), alanine aminotransferase (ALT), total bilirubin (TBIL), Urea nitrogen or Urea (BUN or Urea), serum creatinine (Cr), potassium (K^+), sodium (Na^+), and fasting or non-fasting blood glucose (FBG or BG); urinalysis: urine glucose, urine protein, urine red blood cells, urine white blood cells; coagulation function: prothrombin time (PT), activated partial thromboplastin time (APTT), thrombin time (TT), and fibrinogen (FIB);
10) Pain intensity score: after randomization, the investigator will instruct the subject to assess the NRS score at rest within 5 minutes prior to the start of infusion of loading dose as baseline. The investigator will instruct the subject to assess the NRS scores at rest immediately after the end of loading dose infusion and 20 min, 30 min, 45 min (± 1 min as the assessment point window from immediately after the end of loading dose infusion to 45 min), 1 h, 1.5 h, 2 h, 3 h, 4 h, 5 h, 6 h (± 5 min as the assessment point window from 1 h to 6 h), 8 h, 10 h, 12 h (± 10 min as the assessment point window for 8 h, 10 h, and 12 h), 18 h and 24 h (± 20 min as the assessment point window for 18 h and 24 h) after the infusion;
11) Pain relief score: the investigator will instruct the subject to assess the degree of pain relief (PR) using the Likert scale (0 as no response, 1 mild response, 2 moderate response, 3 significant response, 4 complete response) immediately after the end of loading dose infusion and 20 min, 30 min, 45 min (± 1 min as the assessment point window from immediately after the end of loading dose infusion to 45 min), 1 h, 1.5 h, 2 h, 3 h, 4 h, 5 h, 6 h (± 5 min as the assessment point window from 1 h to 6 h), 8 h, 10 h, 12 h (± 10 min as the assessment point window for 8 h, 10 h, and 12 h), 18 h and 24 h (± 20 min as the assessment point window for 18 h and 24 h) after the infusion;
12) Subject satisfaction score: within 30 min after stopping the PCA pump at 24 h (± 5 min), the subject will score his/her satisfaction with analgesia of the investigational product;
13) Study doctor satisfaction score: within 30 min after stopping the PCA pump at 24 h (± 5 min), the investigator will score his/her satisfaction with analgesia of the investigational product;
14) PK blood sampling: immediately After the end of of loading dose infusion of investigational product, and 20 min, 45 min, 1 h, 3 h, 6 h, 12 h and 24 h after the infusion (see *Clinical Sample Operation Manual* for the specific blood sampling points for each subject);
15) Concomitant medications: the subject's use of any medication other than the protocol-specified investigational products is a concomitant medication. All

concomitant medications taken within 7 days prior to randomization and throughout the study must be recorded in the eCRF, and the record of opioid analgesics must be extended to 3 months prior to randomization;

16) Adverse events: from the time the subject signs the ICF until the end of the safety follow-up period.

Abbreviations

Abbreviations	Full Name
AE	Adverse event
ALT	Alanine aminotransferase
ASA	American society of anesthesiologists
AST	Aspartate aminotransferase
AUC	Area under curve
BMI	Body mass index
BUN	Blood urea nitrogen
CI	Confidence interval
C_{max}	Maximum concentration
Cr	Creatinine
CYP450	Cytochrome P450
D	Day
eCRF	Electronic case report form
FAS	Full analysis set
GCP	Good clinical practice
GLU	Blood glucose
GMP	Good manufacturing practices
h	Hour
Hb	Hemoglobin
IEC	Independent ethics committee
IRB	Institutional review board
ITT	intention to treat
kg	Kilogram
MedDRA	Medical Dictionary for Regulatory Activities
mg	Milligram
min	Minimum
mL	Milliliter
MOR	μ-opioid receptor
MTD	Maximum tolerated dose
NEUT	Neutrophil
NMPA	National medical products administration
NOAEL	No-observed-adverse-effect level
NRS	Numeric rating scales
PLT	Blood platelet
PPS	Per-protocol set
PRO	Protein in urine
PT	Preferred term
RBC	Red blood cell count
SAE	Serious adverse event
SAP	Statistical analysis plan
SOC	System organ classification
SS	Safety set
$t_{1/2}$	Half life
TEAE	Treatment emergent adverse event
T_{max}	Time to maximum concentration
U-GLU	Urine glucose
WBC	White blood cell count

Introduction: Study Background and Scientific Rationale

Study Background

Postoperative pain [1] is an acute pain occurring immediately after surgery, usually lasting no more than 7 days. Postoperative pain is common in thoracic surgery with major trauma and arthroplasty requiring prolonged functional exercise, sometimes needing analgesia for several weeks. Improper management of postoperative pain may lead to a variety of adverse events, such as myocardial ischemia, myocardial infarction, impaired pulmonary function, paralytic ileus, urinary retention, thromboembolism, impaired immune function, and anxiety; poorly controlled pain may also develop into chronic pain, and its nature may also change into neuropathic pain or mixed pain. Therefore, timely and effective postoperative analgesia can not only relieve the patient's pain, but also help the recovery of the disease and have great social and economic benefits.

Opioids are the most commonly used drugs for the treatment of moderate and severe acute and chronic pain. μ-opioid receptor (MOR) is one of the opioids with strong analgesic effect, such as morphine and fentanyl. MOR is one of the G Protein-Coupled Receptors, and classic MOR agonists activate both the G protein-coupled pathway and the β-arrestin-2 signaling pathway. Activation of G protein-coupled pathway is beneficial to produce strong central analgesic effect, but activation of the β-arrestin-2 pathway causes side effects such as gastrointestinal adverse reactions and respiratory depression, which seriously affects the clinical application of such drugs [2].

SHR8554 is a biased MOR agonist that selectively activates the G protein-coupled pathway, whereas it has only a weak activation effect on the β-arrestin-2 pathway. This selective effect ensures that SHR8554 has central analgesic effect while relieving gastrointestinal adverse reactions and reducing the incidence of respiratory depression [3,4].

SHR8554 Injection is an innovative drug independently developed by Jiangsu Hengrui Pharmaceuticals Co., Ltd. It is classified as New Chemical Drug Registration Category 1, and received the clinical approval by the National Medical Products Administration (NMPA) on 25 September 2017. A single-dose Phase 1 clinical study in healthy subjects and a Phase 2 clinical study of analgesia after laparoscopic surgery in the lower abdomen have been completed.

Preclinical Data

Preclinical Pharmacodynamic Studies

In vitro pharmacodynamic results showed that SHR8554 was highly selective for MOR, with dissociation constants (Ki) of 4.2, 1384 and > 5000 nM for the three isoforms of μ-, δ- and κ-

opioid receptor, respectively. SHR8554 activated the G protein-coupled signaling pathways of hMOR, hKOR and hDOR with EC_{50} values of 7.65, 1164 and 559.8 nM, respectively. SHR8554 activated the MOR G protein-coupled pathway in mice, rats, dogs and monkeys with EC_{50} values ranging from 0.22 to 1.17 nM. The effect of SHR8554 on the β-arrestin signaling pathway was approximately 10% of that of morphine. These results suggested that SHR8554 is a highly active and selective agonist of MOR, whereas it has a weak activation effect on the β-arrestin signaling pathway.

In vivo pharmacodynamic study showed that SHR8554 Injection and morphine exhibited similar dose-dependent analgesic effects in various pain treatments in rodents, such as heat-induced pain, mechanical pain, and chemical pain. The equivalent dose ratio of SHR8554 to morphine ranged from approximately 1:3 to 1:9.

Preclinical Toxicology Studies

The results of the acute toxicity study showed that the maximum tolerated dose (MTD) was 4.0 mg/kg in Sprague-Dawley (SD) rats and cynomolgus monkeys after a single intravenous injection.

In the long-term toxicity study in SD rats injected once daily for 28 consecutive days, no abnormalities were observed in general condition, body weight, food consumption, clinical pathology, hematology and blood chemistry, ophthalmic examination, bone marrow examination, urinalysis, gross anatomy, organ weight and coefficient, and histopathological examination, except for transient decreased activity and/or increased muscle tone, decreased flexor reflex, and decreased perineal reflex in rats in each SHR8554 group. Bone marrow micronucleus test was negative. SHR8554 was administered intravenously to SD rats for 28 consecutive days with NOAEL of 2.0 mg/kg. At this dose level, AUC_{last} was 425 ± 48.2 h·ng/mL in females and 514 ± 45.7 h·ng/mL in males after the last dose.

In the long-term toxicity study in cynomolgus monkeys injected once daily for 28 consecutive days, transient symptoms such as transient somnolence, forced convulsions, shallow breathing, and decreased activity occurred after the first dose in monkeys in the 2.0 mg/kg group, which were related to the pharmacological effects of this product. The food consumption was slightly decreased in monkeys in each SHR8554 group during the treatment period and recovered after drug withdrawal. In addition, no obvious abnormal changes were observed in general condition, neurological observation, withdrawal syndrome observation, body weight, body temperature, ophthalmic examination, lead II electrocardiogram indicators, respiratory rate, blood pressure, hematology and blood chemistry, urinalysis, bone marrow examination, gross anatomical observation, organ weight and coefficient, and histopathological examination in monkeys in

SHR8554 groups. SHR8554 was administered intravenously to cynomolgus monkeys for 28 consecutive days with NOAEL of 1.0 mg/kg. At this dose level, AUC_{last} was 417 ± 70.9 h·ng/mL in females and 501 ± 90.6 h·ng/mL in males after the last dose.

No genotoxicity was observed in preclinical studies with SHR8554 Injection, and the risk of genotoxicity in the clinic is presumed to be small.

The NOAEL of SHR8554 Injection was 2.0 mg/kg for male fertility, 1.0 mg/kg for female fertility, and 1.0 mg/kg for early embryonic development. The NOAEL for pregnant rats, embryonic and fetal development was 2.0 mg/kg (AUC_{last} on gestation day 17 was 470 h·ng/mL).

Preclinical Pharmacokinetic Studies

SHR8554 had a rapid onset of action following intravenous or subcutaneous injection with plasma elimination half-lives of approximately 1 and 2 hours in rats and monkeys, respectively, and high plasma clearance. There were no apparent sex differences in pharmacokinetic profiles. SHR8554 was rapidly distributed to most tissues and organs over 10 minutes postdose, with mainly unchanged drug in the plasma and not entering the red blood cells. The exposure of SHR8554 in brain was twice that in plasma and it was not the substrate of major transporters of renal and blood-brain barrier, indicating that SHR8554 could rapidly penetrate the blood-brain barrier, maintain a certain concentration, and cause central analgesic effect. SHR8554 was rapidly and highly metabolized and was excreted predominantly in urine and feces. Plasma protein binding was more than 80% in humans, monkeys, and rats, and free drug concentrations were expected to be low in clinic. *In vitro* studies showed that the major metabolizing enzymes of SHR8554 were CYP2D6, CYP3A4 and CYP3A5, with moderate inhibition of CYP2D6.

Clinical Data

Currently, a Phase 1 clinical trial in healthy adult males (Trial No.: SHR8554-101) and a Phase 2 clinical trial of analgesia after laparoscopic surgery in the lower abdomen (Trial No.: SHR8554-201P) have been completed for SHR8554 Injection.

SHR8554-101 Study Design and Results

(I) Study Design: a total of 59 healthy male subjects were enrolled in the study. A total of 3 groups (A, B, C) were included. A total of 52 subjects were enrolled in Group A, with 6 dose groups (0.25 mg, 0.75 mg, 1.5 mg, 3 mg, 2 mg, 2.5 mg). Of these, only 2 subjects were included in the lowest dose 0.25 mg group, without placebo group, all subjects received the investigational drug, and the intravenous bolus time was 30 min. Group B was an open-label, three-period study, and a total of 6 subjects were enrolled. The subjects received 0.75 mg SHR8554 on Days 1, 3, and 5, respectively, and the intravenous bolus time was 15 min, 5 min,

and 2 min, respectively. During the screening for Groups A and B, if the CYP2D6 enzyme phenotype was found to be poor metabolizers, the subjects would not enter into Group A or B. After completion of the 0.25 mg and 0.75 mg groups, a poor metabolizer group of CYP2D6 enzymes was set up separately. The dose of 1.5 mg was administered by intravenous bolus injection over 30 min. The safety and PK assessments were performed with reference to Group A.

(II) Results:

Safety

A total of 5 moderate TEAEs occurred, all of which were vomiting and all drug-related. All other AEs were mild in severity; there were no SAEs and no AEs leading to withdrawal from the trial. The most common ADRs in Group A included dizziness: in 1 subject (12.5%) in the SHR8554 1.5 mg group, 4 subjects (50.0%) in the 2.5 mg group, and 4 subjects (80.0%) in the 3 mg group, all of which were mild; nausea: in 2 subjects (25.0%) in the SHR8554 2.5 mg group and 4 subjects (80.0%) in the 3 mg group, all of which were mild; vomiting: in 2 subjects (25%) in the SHR8554 1.5 mg group, 1 subject (12.5%) in the 2.5 mg group, and 4 subjects (80.0%) in the 3 mg group, and it was mild in 2 subjects in the 1.5 mg group, and moderate in 5 subjects in the 2.5 mg group and 3 mg group. The most common ADRs in Group B included dizziness: in 4 subjects (66.7%), and the rest were reported in single subjects (16.7%), including white blood cell count decreased, blood uric acid increased, blood pressure decreased, and sinus arrhythmia. ADRs in the poor metabolizers of CYP2D6 enzymes in Group C included white blood cell count decreased and platelet count decreased once each.

PK

After a single dose of SHR8554 by intravenous bolus injection over 30 min in Group A, the plasma concentrations in each dose group of 0.75-3 mg peaked at the end of the intravenous bolus injection, and the median T_{max} values in each dose group were around 0.33-0.50 h; then, there was a bi-exponential decrease in plasma concentration, with no significant difference in the mean $t_{1/2}$ between each dose groups, which was within the range of 6.08-6.96 h, and no dose-dependent trend was observed. After a single 30-min intravenous bolus injection of SHR8554 Injection over the dose range of 0.75 to 3 mg, the exposure of SHR8554 in plasma increased slightly less than dose-proportionally in the high dose group than in the low dose group. In Group B, there was no significant difference in overall AUC over time between different intravenous bolus time groups (2 min, 5 min, 15 min). Since only one subject with poor CYP2D6 enzyme metabolism was included in this study, no clear conclusion could be drawn regarding the effect of CYP2D6 enzyme on PK.

PD

The PD characteristics of the drug were investigated by the duration of non-dominant hand immersion in a 2 °C low-temperature circulating water bath (pain tolerance) and the pain score at different time points before and after the treatment.

The results of pain tolerance showed that, with the exception of 0.25 mg (N = 2, small sample size at this dose level) in Group A, the mean percentage change from baseline in pain tolerance at each measurement time point after administration in other 5 dose levels of the SHR8554 group improved to varying degrees; there was no obvious consistent trend in the percentage change from baseline at each measurement time point during the observation period of pain tolerance in Group B (N = 6). The results of pain score showed that all pain scores at each measurement time point after administration at 2.5mg in Group A improved from the mean baseline, and the trend of change from baseline was not obvious in other dose groups; all pain scores at each measurement time point in the observation period improved from the mean baseline in subjects in Group B (N = 6).

There was no clear trend of correlation between plasma SHR8554 exposure/dose and response in healthy subjects.

Further study background information is provided in the Investigator’s Brochure.

SHR8554-201P Study Design and Results

(1) Study design: a multicenter, randomized, double-blind, placebo/active-controlled trial design was used to investigate the efficacy and safety of SHR8554 Injection for postoperative analgesia after elective laparoscopic surgery in the lower abdomen under general anesthesia. A total of 50 subjects with NRS score at rest ≥ 4 after elective laparoscopic surgery in the lower abdomen under general anesthesia were planned to be enrolled, including 3 SHR8554 Injection groups, 10 subjects each in each dose group, 10 subjects in the placebo group, and 10 subjects in the active control morphine group. If the subject had at least one score ≥ 4 after laparoscopic surgery in the lower abdomen, SHR8554/placebo/morphine will be administered intravenously by an infusion pump at a constant speed over 10 minutes (bolus speed: 30 mL/h) immediately (within 5 minutes after the most recent scoring) for the first time, starting doses: SHR8554 Injection 0.5 mg, 0.75 mg or 1 mg, or morphine 3 mg. Thirty minutes after the first dose, the subjects may self-administer SHR8554 (0.05 mg/dose)/placebo/morphine (1 mg/dose) as needed via Patient Controlled Analgesia (PCA) pump every 10 minutes. PCA administration was stopped 24 h after the starting dose. 3 mL of venous blood was collected before the first dose and at 10 min, 16 h and 24 h after the first dose, respectively, for the analysis of plasma SHR8554 and morphine concentrations.

After administration, the investigator could give antiemetic treatment according to the subject's condition of nausea and vomiting, and tropisetron was preferred for antiemetics (2.5 mg by intravenous injection for the first time, followed by additional doses as needed, and the total dose within 24 h was not more than 10 mg). After administration, for subjects who required rescue analgesia as judged by the investigator due to inadequate analgesia and had NRS score at rest ≥ 4, parecoxib sodium (20 mg by intravenous injection for the first time, followed by additional doses as needed, and the total dose within 24 h was not more than 80 mg) was preferred within 48 h of administration. If the analgesic and antiemetic needs were still not met, the investigator could use other rescue measures as appropriate, and should truthfully record it in the original records and eCRFs.

During the treatment period (D1-D2), the percentage of subjects in each group who did not use rescue analgesics from 0 to 24 h post-dose was calculated. For subjects who received rescue analgesics, the cumulative amount of rescue analgesics was calculated for each group. During the study (D1-D3), the incidence and severity of AEs at 0-48 h post-dose were recorded in each group; the cumulative amount of antiemetics at 0-48 h post-dose was calculated in each group; and postoperative analgesia satisfaction scores were rated by the subjects and the study doctor at 48 h post-dose in each group.

The subjects were discharged from the study after completing all assessments in the study flow chart on the third postoperative day.

(II) Results:

Efficacy results

Primary endpoint: percentage of subjects who did not use rescue analgesics from 0 h to 24 h postdose. In the FAS, the percentage of subjects who did not use rescue analgesics from 0 to 24 h postdose was 60.00% in the SHR8554 0.5 mg group, 60.00% in the SHR8554 0.75 mg group, 100.00% in the SHR8554 1 mg group, 60.00% in the morphine group, and 40.00% in the placebo group, respectively. The rate difference and its 95% confidence interval between SHR8554 1 mg group and placebo group was 60.00% (21.27%, 87.84%); P value was 0.0108, and the percentage of subjects who did not use rescue analgesics was higher in SHR8554 1.0 mg group than in the placebo group. There were no statistically significant differences in SHR8554 0.5 mg group versus placebo group, SHR8554 0.75 mg group versus placebo group, and morphine group versus placebo group ($p > 0.05$). The analysis conclusions in PPS were consistent with those in the FAS.

Secondary endpoints: 1. Cumulative amount of rescue analgesics from 0 h to 24 h: in the FAS, the cumulative amount of rescue analgesics from 0 h to 24 h post-dose was 16.0 ± 22.7 mg in

the SHR8554 0.5 mg group, 10.0 ± 14.1 mg in the SHR8554 0.75 mg group, 0.0 ± 0.0 mg in the SHR8554 1 mg group, 14.0 ± 21.2 mg in the morphine group, and 20.0 ± 21.1 mg in the placebo group, respectively. The inter-group difference and its 95% confidence interval between SHR8554 1 mg group and placebo group was -20.0 (-36.2, -3.8) mg; P-value was 0.0164, and the cumulative amount of rescue analgesics was less in SHR8554 1.0 mg group than in placebo group. There were no statistically significant differences in SHR8554 0.5 mg group versus placebo group, SHR8554 0.75 mg group versus placebo group, and morphine group versus placebo group ($p > 0.05$). The analysis conclusions in PPS were consistent with those in the FAS. 2. Subject satisfaction score at 48 h post-dose: in the FAS, the subject satisfaction score at 48 h post-dose in each group was 9.4 ± 0.8 in the SHR8554 0.5 mg group, 8.9 ± 1.9 in the SHR8554 0.75 mg group, 9.4 ± 1.0 in the SHR8554 1 mg group, 9.2 ± 0.9 in the morphine group, and 8.6 ± 1.6 in the placebo group, respectively. There was no statistically significant difference between the groups ($p > 0.05$). The analysis conclusions in PPS were consistent with those in the FAS. 3. Study doctor satisfaction score at 48 h post-dose: in the FAS, the study doctor satisfaction score at 48 h post-dose in each group was 7.8 ± 2.3 in the SHR8554 0.5 mg group, 8.3 ± 1.9 in the SHR8554 0.75 mg group, 8.7 ± 0.9 in the SHR8554 1 mg group, 7.8 ± 2.0 in the morphine group, and 7.3 ± 1.6 in the placebo group, respectively. There was no statistically significant difference between the groups ($p > 0.05$). The analysis conclusions in PPS were consistent with those in the FAS.

Safety Endpoints

Adverse events: 1. Overall AEs: a total of 254 adverse events occurred in 48 of 51 subjects, with an incidence of 94.12%. Of these, a total of 38 events occurred in 9 of 10 subjects in the SHR8554 0.5 mg group, with an incidence of 90.00%; a total of 50 events occurred in 9 of 10 subjects in the SHR8554 0.75 mg group, with an incidence of 90.00%; a total of 71 events occurred in 9 of 10 subjects in the SHR8554 1 mg group, with an incidence of 90.00%; a total of 50 events occurred in 11 of 11 subjects in the morphine group, with an incidence of 100.00%; and a total of 45 events occurred in 10 of 10 subjects in the placebo group, with an incidence of 100.00%. 2. Treatment-emergent adverse events: a total of 217 TEAEs occurred in 48 of 51 subjects, with an incidence of 94.12%. Of these, 9 subjects in the SHR8554 0.5 mg group had a total of 29 TEAEs, with an incidence of 90.00%; 9 subjects in the SHR8554 0.75 mg group had a total of 46 TEAEs, with an incidence of 90.00%; 9 subjects in the SHR8554 1 mg group had a total of 60 TEAEs, with an incidence of 90.00%; 11 subjects in the morphine group had a total of 46 TEAEs, with an incidence of 100.00%; and 10 subjects in the placebo group had a total of 36 TEAEs, with an incidence of 100.00%. All AEs were of mild to moderate intensity,

and no severe adverse events occurred. 3. Serious adverse events: one SAE (SHR8554 0.75 mg group) occurred during the study, with an incidence of 1.96%. The SAE was infection with the outcome of prolonged hospitalization. The SAE was judged by the investigator to be unlikely related to the study drug. 4. AE leading to withdrawal: an AE leading to withdrawal occurred in 1 of 51 subjects (morphine group), with an incidence of 1.96%. It was an anaphylactoid reaction, related to morphine, resulting in permanent discontinuation. 5. Study drug-related adverse events: a total of 63 study drug-related adverse events occurred in 31 of 51 subjects, with an incidence of 60.78%. Of these, a total of 5 events occurred in 4 of 10 subjects in the SHR8554 0.5 mg group, with an incidence of 40.00%;

a total of 14 events occurred in 5 of 10 subjects in the SHR8554 0.75 mg group, with an incidence of 50.00%; a total of 13 events occurred in 7 of 10 subjects in the SHR8554 1 mg group, with an incidence of 70.00%; a total of 17 events occurred 7 of 11 subjects in the morphine group, with an incidence of 63.64%; and a total of 14 events occurred in 8 of 10 subjects in the placebo group, with an incidence of 80.00%.

AEs with incidence ≥ 10% in the SHR8554 0.5 mg group were categorized according to the MedDRA classification system, in the order from high to low: gastrointestinal disorders (40%). AEs with incidence > 10%: vomiting (30%) and nausea (20%); the incidence of other AEs was 0%.

AEs with incidence ≥ 10% in the SHR8554 0.75 mg group were categorized according to the MedDRA classification system, in the order from high to low: gastrointestinal disorders (50%), investigations (10%), cardiac disorders (10%), and nervous system disorders (10%). AEs with incidence > 10%: nausea (40%), vomiting (40%), C-reactive protein increased (10%), blood potassium decreased (10%), sinus bradycardia (10%), and dizziness (10%); the incidence of other AEs was 0%.

AEs with incidence ≥ 10% in the SHR8554 1 mg group were categorized according to the MedDRA classification system, in the order from high to low: gastrointestinal disorders (40%), investigations (20%), cardiac disorders (20%), and skin and subcutaneous tissue disorders (10%). AEs with incidence > 10%: nausea (40%), vomiting (20%), electrocardiogram QT prolonged (10%), respiratory rate increased (10%), blood pressure decreased (10%), sinus tachycardia (10%), sinus arrhythmia (10%), and rash (10%); the incidence of other AEs was 0%.

AEs with incidence ≥ 10% in the morphine group were categorized according to the MedDRA classification system, in the order from high to low: gastrointestinal disorders (54.55%) and investigations (18.18%). AEs with incidence > 10%: nausea (45.45%) and vomiting (45.45%);

AEs with incidence > 5%: electrocardiogram QT prolonged (9.09%), oxygen saturation decreased (9.09%), dizziness (9.09%) and anaphylactoid reaction (9.09%). The incidence of other AEs was 0%.

AEs with incidence ≥ 10% in the placebo group were categorized according to the MedDRA classification system, in the order from high to low: gastrointestinal disorders (50%), investigations (40%), and cardiac disorders (10%). AEs with incidence > 10%: vomiting (40%), nausea (20%), electrocardiogram QT prolonged (20%), constipation (10%), urine ketone body present (10%), electrocardiogram T wave abnormal (10%), heart rate decreased (10%), blood pressure increased (10%), and sinus bradycardia (10%). The incidence of other AEs was 0%.

Cumulative amount of antiemetics from 0 h to 48 h: the cumulative amount of antiemetics from 0 h to 48 h post-dose in each group was 0.250 ± 0.791 mg in the SHR8554 0.5 mg group, 1.250 ± 1.768 mg in the SHR8554 0.75 mg group, 2.000 ± 3.291 mg in the SHR8554 1 mg group, 2.000 ± 2.297 mg in the morphine group, and 1.000 ± 1.748 mg in the placebo group, respectively.

Based on this multicenter, randomized, double-blind, placebo/active-controlled phase 2 clinical trial of SHR8554 Injection for analgesia after elective laparoscopic surgery in the lower abdomen under general anesthesia, the following conclusions were drawn:

In terms of efficacy, this study preliminarily showed that the SHR8554 Injection 1.0 mg dose was superior to placebo in the treatment of postoperative laparoscopic pain in subjects undergoing elective abdominal laparoscopic surgery under general anesthesia, and comparable to morphine; the SHR8554 Injection 0.75 mg dose was comparable to morphine in the treatment of postoperative laparoscopic pain, and showed certain efficacy even though without statistically significant difference compared with placebo.

In terms of safety, when SHR8554 Injection 0.75 mg and 1 mg were used for the treatment of pain after elective laparoscopic surgery in the lower abdomen under general anesthesia, all AEs experienced by the subjects were mild to moderate, with no serious adverse reactions, and the overall safety was good.

Further study background information is provided in the Investigator’s Brochure.

Scientific Rationale for Study Design

Selection of Dose

Based on the results of the Phase 1 SAD study of SHR8554 Injection, the recommended doses in the SHR8554-201P study (loading doses 0.5 mg, 0.75 mg and 1.0 mg, and a single additional 0.05 mg by PCA pump) were obtained by fitting based on the relationship between *in vivo* exposure and adverse reactions; referring to the results of the 201P study, the efficacy of

SHR8554 Injection 1.0 mg was significantly superior to the placebo, with no difference from the active control morphine. Although SHR8554 Injection 0.75 mg group did not show significant statistical difference from the control group, it showed an effective trend. In terms of safety, all adverse reactions were mild to moderate in both groups, no drug-related SAEs occurred, and the safety was good. Therefore, 0.75 mg and 1.0 mg groups were retained to continue validating their efficacy in this study. Therefore, in this study, the loading dose of SHR8554 Injection was 0.75 mg or 1.0 mg, and the single effective press dose by PCA pump was 0.05 mg.

Selection of Control Drug

In 2007, the Center for Drug Evaluation (CDE) of the National Medical Products Administration issued "Key Points to Consider in Clinical Trials for Analgesic Drug Registration in China"[5], which states: "Randomized controlled studies comparing with placebo are required". A placebo control will be used to investigate the true adverse events of the investigational product and to compare the efficacy of the investigational product with that of placebo. The analgesic effect of morphine is clear, so it is used as a positive control to compare with the safety and efficacy of the investigational drug.

Risks and Benefits

According to the results of the SHR8554 Phase 1 SAD study in healthy subjects and 201P study, common adverse reactions included dizziness, nausea, and vomiting, with no SAEs or severe AEs related to the investigational product. The safety precautions established in this trial are as follows: for the treatment of drug overdose, the main focus should be placed on airway opening and auxiliary facilities and the reconstruction of controlled ventilation. If drug overdose coincides with circulatory shock and pulmonary edema, appropriate measures (including oxygenation and vasopressors) should be taken. Cardiac arrest and arrhythmias may require cardiopulmonary resuscitation and defibrillation. As an antagonist of opioid receptor, naloxone antagonizes respiratory depression caused by SHR8554 Injection or morphine, therefore, an appropriate amount of this antagonist may be considered by intravenous infusion in case of respiratory depression. The study site must be equipped with necessary medical rescue equipment, first aid drugs and emergency measures. Emergency medical events or unexpected disasters shall be handled in accordance with clinial SOPs. In addition, the investigator can decide whether other appropriate measures are required according to the actual situation.

SHR8554 Injection has fewer side effects than classic MOR agonists in terms of mechanism of action and can provide another option for clinical postoperative pain management.

Study Objective and Efficacy Variables

Study Objective

To evaluate the efficacy and safety of SHR8554 Injection for analgesia after abdominal surgery.

Efficacy Variables

Primary Efficacy Variable

Time-weighted Sum of Pain Intensity Differences at rest up to 24 h after starting infusion of loading dose of investigational product ($SPID_{24}$).

Secondary Efficacy Variables

1) Time-weighted Sum of Pain Intensity Differences at rest at 6 h, 12 h, 18 h, and 12-24 h ($SPID_{6}$, $SPID_{12}$, $SPID_{18}$, $SPID_{12-24}$);
2) Time-weighted Total Pain Relief at 6 h, 12 h, 18 h, 24 h, and 12-24 h ($TOTPAR_{6}$, $TOTPAR_{12}$, $TOTPAR_{18}$, $TOTPAR_{24}$, $TOTPAR_{12-24}$);
3) Time to the first use of rescue analgesics;
4) Cumulative amount of rescue analgesics from 0 h to 24 h;
5) Number of treatments of rescue analgesia from 0 h to 24 h;
6) Total number of presses and number of effective presses of PCA pump from 0 h to 24 h;
7) Percentage of subjects who do not use rescue analgesics from 0 h to 24 h;
8) The subject's satisfaction score with analgesic treatment;
9) The investigator's satisfaction score with analgesic treatment.

Study Design

The study will use a multicenter, randomized, double-blind, placebo/active-controlled trial design.

The study process consists of a screening period, a treatment period, and a follow-up period.

Screening period: from signing the ICF to randomization, with a maximum of no more than 7 days, and the day of randomization will be recorded as D1

The subject will sign the informed consent form and complete the screening according to the study flow chart. The subject will undergo abdominal surgery (laparotomy or laparoscopy) under general anesthesia (total intravenous anesthesia or intravenous-inhalation combined anesthesia). Sevoflurane is selected as inhalation anesthetics, propofol injection is selected as intravenous anesthetics, and the dose of general anesthetics will be determined on an individual basis. Sufentanil injection (0.2-0.6 μg/kg) is selected as opioid analgesics during the anesthetic induction period. Remifentanil injection (0.1-0.5 μg/kg/min) is selected as opioid analgesics

during the anesthetic maintenance period, sufentanil injection (0.1-0.2 μg/kg/time) can be added, and the doses can be adjusted appropriately according to the needs of surgery and the subject's individual conditions. The type and dose of muscle relaxants and other anesthetic adjuvant drugs are at the discretion of the anesthesiologist and/or surgeon. 2.5 μg sufentanil will be intravenously injected immediately (± 5 min) after the surgery (the last suture is completed) to prevent postoperative breakthrough pain.

Within 4 hours after the surgery, the investigator will instruct the subjects to assess the pain intensity (PI) at rest using the Numeric Rating Scale (NRS, 0-10 for varying degrees of pain, 0 for painlessness, larger numbers for more severe pain, and 10 for the worst pain), and it is recommended that the interval between the two assessments should not exceed 30 minutes. The subjects with NRS score ≥ 4 at rest at any time within 4 hours after the surgery can be randomized (it is recommended to be randomized within 10 min after the NRS score reaches 4 or more for the first time). The subjects will be randomized in a 1:1:1:1 ratio to one of the following groups: SHR8554 Injection 0.75 mg group, SHR8554 Injection 1.0 mg group, morphine hydrochloride injection group, and placebo group.

Treatment period: from randomization to 24 h, with the time to start infusion of the loading dose of investigational product recorded as 0 h.

After randomization, the investigator should infuse the investigational product to the subject as soon as possible (within 15 min after the randomization). The loading dose of the investigational product will be 5 mL, administered by an infusion pump, and the intravenous infusion time will be approximately 10 min. The Patient Controlled Analgesia (PCA) pump containing the investigational product can be initiated approximately 30 min after the start of infusion of the loading dose. The subject can receive 1 mL of the investigational product at a single press of PCA pump, and the minimum interval between two adjacent effective presses is 10 minutes. The subject will continue using the PCA pump until 24 h (± 5 min).

After the end of of loading dose infusion, rescue analgesia may be given if the investigator determines that the investigational product can not meet the subject's analgesic needs (NRS ≥ 4 points). The first choice of rescue analgesics is parecoxib sodium for injection (20 mg for the first intravenous injection, followed by additional doses as needed, with a total dose of not more than 80 mg over 24 h), and sufentanil injection (2.5 μg for a single intravenous injection) can be used after reaching the maximum cumulative dose of parecoxib sodium for injection.

After randomization, the investigator will instruct the subject to assess the NRS score at rest within 5 minutes prior to the start of infusion of loading dose as baseline. The investigator will

instruct the subject to assess the NRS scores at rest immediately after the end of loading dose infusion and 20 min, 30 min, 45 min (± 1 min as the assessment point window from immediately after the end of loading dose infusion to 45 min), 1 h, 1.5 h, 2 h, 3 h, 4 h, 5 h, 6 h (± 5 min as the assessment point window from 1 h to 6 h), 8 h, 10 h, 12 h (± 10 min as the assessment point window for 8 h, 10 h, and 12 h), 18 h, and 24 h (± 20 min as the assessment point window for 18 h and 24 h) after the infusion. At the same time as each assessment of NRS score, the investigator will instruct the subject to assess the degree of pain relief (PR) using the Likert scale (0 as no response, 1 mild response, 2 moderate response, 3 significant response, 4 complete response).

PK blood sampling: three PK blood samples will be collected from each subject for plasma concentration analysis and drug metabolizing enzyme gene analysis as specified in the *Clinical Sample Operation Manual*. The PK blood sampling time points include: immediately After the end of of loading dose infusion of investigational product, and 20 min, 45 min, 1 h, 3 h, 6 h, 12 h and 24 h after the infusion (see *Clinical Sample Operation Manual* for the specific blood sampling points for each subject).

Blood samples at each time point can be collected at any time within the time window (see the *Clinical Sample Operation Manual* for the time window of blood sample collection), and the time of blood collection should be accurately recorded. If the PK sampling time coincides with the pain scoring time, the pain scoring should be performed first. Approximately 3 mL of venous blood will be collected at each collection point into K_2EDTA tubes, pretreated and preserved as required until use for analysis.

The subject will be required to have continuous ECG monitoring (brief interruptions are allowed due to transit and the subjects are allowed to perform necessary activities) from 0 h to stopping the infusion of investigational product.

The investigator should perform 12-lead ECG and record the subject's vital signs (blood pressure [BP], respiratory rate [RR], heart rate [HR], body temperature [T]) and transdermal oxygen saturation (SpO_2) from randomization to 0 h, and at 1 h, 2 h, 3 h, 6 h (± 5 min as the assessment point window from 1 h to 6 h), 12 h and 24 h (± 15 min as the assessment point window for 12 h and 24 h assessments). The investigator will decide whether additional 12-lead ECG is required according to the subject's clinical condition.

During the treatment period, the time to the first use of rescue analgesics, the cumulative amount of rescue analgesics from the end of the infusion of loading dose to 24 h, the number of treatments of rescue analgesics, the total number of PCA pump presses within 24 h, the number of effective presses of PCA pump, and the time of effective presses of PCA pump will

be recorded. Within 30 min after stopping the PCA pump at 24 h (± 5 min), the subject and the investigator will score their satisfaction with analgesia of the investigational product, respectively.

Follow-up Period: 24 h to D4 ± 1

During the follow-up period, the subject will complete relevant examinations and complete the safety assessment according to the study flow chart.

Subject Selection and Withdrawal

Inclusion of eligible subjects is a key step to achieve the intended objectives of this clinical trial. The subjects must meet the following criteria to be allowed to participate in this study. All medical or non-medical conditions of each subject are within consideration in deciding whether he/she is eligible for the study.

Inclusion Criteria

Subjects will be eligible for enrollment into the study if all of the following criteria are met:

1) Before starting any trial-related activity, the subject must voluntarily sign the ICF, fully understand the purpose and significance of this trial, and willingly comply with the study procedures;
2) Subjects who will undergo selective abdominal surgery (laparotomy or laparoscopy) under general anesthesia for ≥ 1 h;
3) 18 to 65 years of age, male or female;
4) 18.0 kg/m^2 ≤ body mass index (BMI) ≤ 28.0 kg/m^2;
5) ASA I-II;
6) NRS ≥ 4 at rest at any time within 4 h after the surgery.

Exclusion Criteria

Subjects will be ineligible for enrollment into the study if any of the following criteria is met:

1. Disease conditions

1) Have a prior history of vestibular dysfunction or have dizziness, nausea, retching or vomiting within 1 week prior to screening;
2) Have a prior history of ischemic stroke or transient ischemic attack (TIA);
3) Have a prior history of difficult airway, such as obstructive sleep apnea syndrome;
4) Have prior reflux esophagitis;
5) Have a history of myocardial infarction or unstable angina pectoris, or severe arrhythmia such as atrioventricular block grade II or higher, or NYHA functional class II or higher within 6 months prior to screening;

6) Have a prior history of psychiatric disorders (e.g. schizophrenia, depression, etc.), cognitive dysfunction, epilepsy, or Parkinson's syndrome;
7) Have advanced malignancies or malignant tumors with extensive metastases;
2. General examination/laboratory tests
1) Subjects who do not receive formal antihypertensive therapy or have inadequate blood pressure control (systolic blood pressure ≥ 160 mmHg or ≤ 90 mmHg at screening, and/or diastolic blood pressure ≥ 100 mmHg or ≤ 60 mmHg at screening, excluding abnormalities from entering the operation room to postoperative extubation of endotracheal intubation);
2) QTc at screening: > 450 ms for males and > 470 ms for females (QTc calculated by Fridericia's formula);
3) Transdermal oxygen saturation (SpO_2) < 90% at screening (excluding abnormalities from entering the operation room to postoperative extubation of endotracheal intubation);
4) Poor glycemic control at screening: random blood glucose ≥ 11.1 mmol/L;
5) Abnormal liver function at screening: aspartate aminotransferase (AST) and/or alanine aminotransferase (ALT) ≥ 1.5 × ULN and/or total bilirubin (TBIL) ≥ 1.5 × ULN;
6) Abnormal renal function at screening: serum creatinine (Cr) ≥ 1.5 × ULN;
7) Coagulation abnormalities at screening: prolonged prothrombin time (PT) exceeding the upper limit of normal by 3 seconds and/or prolonged activated partial thromboplastin time (APTT) exceeding the upper limit of normal by 10 seconds;
8) Have positive infectious disease screening for hepatitis B surface antigen (HBsAg), hepatitis C antibody (HCVAb), treponema pallidum antibody or human immunodeficiency virus (HIV) antibody at screening.
3. Drug use
1) Known allergies to opioids and other drugs that may be used during the trial, such as nonsteroidal anti-inflammatory drugs, antiemetics, etc.;
2) Consecutive use of opioid analgesics for more than 10 days for any reason within 3 months prior to randomization, or the time of last dose is less than 5 half-lives from randomization, including but not limited to the following drugs with analgesic effect: selective α_2-adrenoceptor agonists, opioid agonists/antagonists, nonsteroidal anti-inflammatory drugs, sedatives, monoamine oxidase inhibitors, glucocorticoids, CYP2D6, CYP3A4 and CYP3A5 inhibitors/inducers, antihistamines, antiepileptics, anxiolytics, antidepressants, etc. Refer to the Table of Prohibited Drugs for specific drugs;
4. Others
1) Have a history of drug abuse and/or alcohol abuse within 1 year prior to screening, i.e.,

more than 15 g of alcohol per day on average (15 g alcohol is equivalent to 450 mL of beer or 150 mL of wine or 50 mL of low-alcoholic liquor);

2) Positive drug abuse screening result at screening;
3) Pregnant or lactating women;
4) Subjects who have a birth plan, are unwilling or unable to take effective contraceptive measures from 30 days before screening to half a year after the end of the trial;
5) Subjects who participated in other drug clinical trials (defined as receiving investigational product or placebo) within 3 months prior to screening;
6) Other conditions that in the opinion of the investigator make the subject unsuitable to participate in this clinical trial.

Investigational Products

Overview of Investigational Products

Supplies

The investigational product (SHR8554 Injection and placebo) are prepared in a workshop that complies with GMP [Good Manufacturing Practices] and has passed the test according to the specifications approved by the National Medical Products Administration. Morphine hydrochloride injection is manufactured by Shenyang First Pharmaceutical Co., Ltd., Northeast Pharmaceutical Group.

Dosage Form and Strength

SHR8554 Injection, dosage form: injection, strength: 1 mL: 1 mg, 5 mL: 5 mg, manufactured by Jiangsu Hengrui Pharmaceuticals Co., Ltd. Storage conditions: sealed, stored below 30 °C.

Morphine hydrochloride injection, dosage form: injection, strength: 1 mL: 10 mg, manufactured by Shenyang First Pharmaceutical Co., Ltd., Northeast Pharmaceutical Group. Storage conditions: protected from light and sealed.

Placebo, dosage form: injection, strength: 5 mL, manufactured by Jiangsu Hengrui Pharmaceuticals Co., Ltd. Storage conditions: sealed, stored below 30 °C.

Management, Dispensing and Return

The investigational products in this clinical study are managed in accordance with relevant regulations. The unblinded drug management personnel shall prepare the investigational products according to the corresponding group of the subject randomization number. The clinical study institution shall sign the drug receipt form in duplicate, and the clinical study institution and the sponsor shall each keep one copy. The test facility should store all sealed drugs below 30 °C in a special double-locked cabinet kept by two persons according to the

storage requirements of the investigational products. Dispensing and return of each investigational product should be timely documented in the corresponding record sheet. During and after the study, the remaining drugs, packaging boxes and labels shall be promptly collected. The two parties shall sign the drug recovery form. All clinical investigational products shall be properly kept, and submitted to the sponsor after the study. The clinical investigational products shall not be transferred to any non-clinical trial participants or third parties. The unblinded monitor is responsible for monitoring the supply, use, storage of the study drugs and disposal of the remaining drugs.

Configuration of Investigational Products and Setting of PCA Pump Parameters

Refer to relevant SOPs for configuration of investigational products and setting of PCA pump parameters.

Destruction

Since SHR8554 Injection is a narcotic drug, it needs to be destroyed according to the requirements of the drug administration at each site, and the sponsor or its authorized personnel may provide guidance on the destruction of unused investigational products. If the study site is authorized to destroy the investigational products, the investigator must ensure that the destruction complies with applicable provisions such as applicable environment regulations, site policies, etc., and provide the process of destruction. All destruction should be well documented.

Randomization and Blinding

Using a central randomization method, 528 subjects will be randomized in a 1:1:1:1 ratio to 4 treatment groups: SHR8554 Injection 0.75 mg, SHR8554 Injection 1.0 mg, placebo, and morphine hydrochloride injection.

The random number table will be generated by an unblinded statistician of the statistical team using SAS 9.4, and the blinding will be uniformly completed by an unblinded statistician who does not participate in the study. Randomization will be performed using a central randomization system (Interactive Web Response Randomization System, IWRS). The subjects, investigators, and all personnel involved in the trial or clinical assessment will remain blinded to the true treatment (except the unblinded drug management team) from the randomization until the database is locked.

Emergency Unblinding

If the treatment grouping information must be unblinded during the study due to urgent management of SAE, or to decide the subsequent treatment, the site should promptly notify the sponsor of the reasons for unblinding, and the principal investigator should submit an

application for unblinding. If the investigational product is unblinded, the sponsor, the investigator at each site and the Ethics Committee must be notified as soon as possible. The unblinding time, reason and results must be recorded in the source documents. If any centrally blinded personnel become aware of the investigational product information (unblinding), the use of the investigational product should be discontinued immediately and the subject must be withdrawn from the study.

Project Unblinding

After the Blind Review, the database will be locked. Based on the application by the statistician, the investigator, statistician and sponsor will perform unblinding review at the same time, i.e., the subject's treatment group will be notified to the biostatistician for statistical analysis of all data.

Treatments

Dosage Regimen

SHR8554 Injection 0.75 mg group: the loading dose is 0.75 mg (5 mL), and the single infusion dose by PCA pump is 0.05 mg (1 mL).

SHR8554 Injection 1.0 mg group: the loading dose is 1.0 mg (5 mL), and the single infusion dose by PCA pump is 0.05 mg (1 mL).

Morphine hydrochloride injection group: the loading dose is 3.0 mg (5 mL) and the single infusion dose by PCA pump is 1.0 mg (1 mL).

Placebo (saline, i.e., 0.9% sodium chloride injection) group: the loading dose is 5 mL of normal saline and the single infusion dose by the PCA pump is 1 mL of normal saline.

Dosing Records

Site unblinded personnel will prepare and record the study drug in accordance with the study drug manual. The study site's file system should include all related or necessary information for the study drug preparation and dosing.

Rescue Treatments

After the end of of loading dose infusion of the investigational product, parecoxib sodium for injection will be first used as a rescue analgesic if the investigator determines that the investigational product can not meet the subject's analgesic needs (NRS ≥ 4 points). Dose of parecoxib sodium for injection: 20 mg will be injected intravenously for the first time, followed by additional doses as needed, with a total dose of not more than 80 mg over 24 h. Sufentanil injection (2.5 μg for a single intravenous injection) can be used after reaching the maximum cumulative dose of parecoxib sodium for injection. Rescue analgesics should be truthfully documented in the original records and eCRFs. After the treatment period, the subject's

subsequent analgesics and dosages will be at the discretion of the investigator according to the hospital's routine practices.

Concomitant Medications

The subject's use of any medication other than the protocol-specified investigational products is a concomitant medication. All concomitant medications taken within 7 days prior to randomization and throughout the study must be recorded in the eCRF, and the record of opioid analgesics must be extended to 3 months prior to randomization;

Concomitant medications that are vehicle or supplements of electrolytes, nutrients, energy and vitamins are not required to be recorded in the eCRF, except for medications for AEs.

Prohibited medications and prohibited periods from randomization to the end of the follow-up period are shown in the following table (similar drugs include but are not limited to those listed in the table below), with the exception of drugs used for general anesthesia during surgery, drugs that are allowed as specified in the protocol, and analgesics used at the discretion of the investigator during the follow-up period. Glucocorticoids used to manage postoperative complications will not be recorded as protocol violations.

Table 5-1 Prohibited Medications

Class of Drug	Drug Name	Prohibited Period
Selective α_2-adrenoceptor agonists with analgesic effects	Dexmedetomidine hydrochloride injection, clonidine, etc.	Randomization ~ end of treatment period
Opioid agonists/antagonists	Oxycodone, pentazocine, nalbuphine, butorphanol, etc.	Randomization ~ end of follow-up period
Non-steroidal anti-inflammatory drugs	Piroxicam, nabumetone, ibuprofen, celecoxib, diclofenac sodium, etc.	Randomization ~ end of treatment period
Sedatives	Benzodiazepines (e.g., diazepam, flurazepam, oxazepam, chlordiazepoxide, triazolam, alprazolam, estazolam, midazolam, etc.)	Randomization ~ end of treatment period
	Barbiturates (phenobarbital, secobarbital, amobarbital, pentobarbital, sodium thiopental, etc.)	
	Other sedatives (magnesium sulfate, chloral hydrate, etc.)	
Monoamine oxidase inhibitor	Phenelzine, tranylcypromine, selegiline, pargyline, furazolidone, procarbazine, etc.	Randomization ~ end of treatment period

Glucocorticosteroids	Prednisone, methylprednisolone, dexamethasone, betamethasone, etc.	Randomization ~ end of treatment period
CYP2D6, CYP3A4 and CYP3A5 inhibitors/inducers	Amiodarone, macrolides, isoniazid, ketoconazole, carbamazepine, rifampin, etc.	Randomization ~ end of treatment period
Antihistamines	Diphenhydramine, chlorpheniramine, promethazine, cetirizine, dramamine, etc.	Randomization ~ end of treatment period
Anxiolytic/antidepressant/antiepileptic drugs	Phenothiazines, tricyclic antidepressants, carbamazepine, etc.	Randomization ~ end of treatment period

Study Procedures

The study process consists of a screening period, a treatment period, and a follow-up period.

Screening Period: From Signing the ICF to Randomization, With a Maximum of No More Than 7 Days, and the Day of Randomization Will Be Recorded as D1

At screening, laboratory test results (hematology, urinalysis, blood biochemistry, coagulation) and electrocardiograms within 7 days prior to randomization will be acceptable, and infectious disease screening results within 30 days prior to randomization will be acceptable. Any examination result beyond its normal range should be determined to be clinically significant or not by the study doctor from a professional perspective.

- Obtain the written ICF signed by the subject;
- Medical histories: including past medical histories and treatments, medication before screening, recent participation in drug or medical device trials, smoking and alcohol consumption history, allergy history;
- Collect demographic data: date of birth (based on the valid identification card), gender, ethnicity, height, weight, etc.;
- Complete physical examination: including overall appearance, skin and mucosa, lymph nodes, head and neck, chest, abdomen, muscles and bones, nervous system, and other parts of the body;
- Vital signs: blood pressure (BP), heart rate (HR), respiratory rate (RR), body temperature (T);
- Oxygen saturation (SpO_2) measurement;
- 12-lead ECGs;
- Drug abuse screening: including methamphetamine, dimethyldioxyamphetamine, tetrahydrocannabinol acid, ketamine, and morphine;

- Pregnancy test for women: non-menopausal women should perform blood or urine pregnancy test;
- Infectious disease screening: including hepatitis B surface antigen, hepatitis C antibody, HIV antibody, treponema pallidum antibody;
- Laboratory tests: including hematology, urinalysis, blood chemistry and coagulation;
- The subject will undergo abdominal surgery (laparotomy or laparoscopy) under general anesthesia. 2.5 μg sufentanil will be intravenously injected immediately (± 5 min) after the surgery (the last suture is completed) to prevent postoperative breakthrough pain.
- The subjects with NRS score ≥ 4 at rest at any time within 4 hours after the surgery can be randomized (it is recommended to be randomized within 10 min after the NRS score reaches 4 or more for the first time). The subjects will be randomized in a 1:1:1:1 ratio to one of the following groups: SHR8554 Injection 0.75 mg group, SHR8554 Injection 1.0 mg group, morphine hydrochloride injection group, and placebo group;
- Concomitant medications and non-drug therapies as well as AEs will be collected.

Note: electrocardiograms may be retested twice at screening, at least 10 min apart, while other tests at screening can be repeated once at most.

Treatment Period: From Randomization to 24 h, With the Time to Start Infusion of the Loading Dose of Investigational Product Recorded as 0 h.

- Analgesic treatment with investigational product: after randomization, the investigator should infuse the investigational product to the subject as soon as possible (within 15 min after the randomization). The loading dose of the investigational product will be 5 mL, administered by an infusion pump, and the intravenous infusion time will be approximately 10 min. The Patient Controlled Analgesia (PCA) pump containing the investigational product can be initiated approximately 30 min after the start of infusion of the loading dose. The subject can receive 1 mL of the investigational product at a single press of PCA pump, and the minimum interval between two adjacent effective presses is 10 minutes. The subject will continue using the PCA pump until 24 h (± 5 min).
- Rescue analgesia: after the end of of loading dose infusion, rescue analgesia may be given if the investigator determines that the investigational product can not meet the subject's analgesic needs (NRS ≥ 4 points). The first choice of rescue analgesics is parecoxib sodium for injection (20 mg for the first intravenous injection, followed by additional doses as needed, with a total dose of not more than 80 mg over 24 h), and

sufentanil injection (2.5 μg for a single intravenous injection) can be used after reaching the maximum cumulative dose of parecoxib sodium for injection.

- NRS pain intensity score and pain relief score: after randomization, the investigator will instruct the subject to assess the NRS score at rest within 5 minutes prior to the start of infusion of loading dose as baseline. The investigator will instruct the subject to assess the NRS scores at rest immediately after the end of loading dose infusion and at 20 min, 30 min, 45 min (± 1 min as the assessment point window from immediately after the end of loading dose infusion to 45 min), 1 h, 1.5 h, 2 h, 3 h, 4 h, 5 h, 6 h (± 5 min as the assessment point window from 1 h to 6 h), 8 h, 10 h, 12 h (± 10 min as the assessment point window for 8 h, 10 h, and 12 h), 18 h, and 24 h (± 20 min as the assessment point window for 18 h and 24 h) after the infusion. At the same time as each assessment of NRS score, the investigator will instruct the subject to assess the degree of pain relief (PR) using the Likert scale (0 as no response, 1 mild response, 2 moderate response, 3 significant response, 4 complete response).
- ECG monitoring: the subject will be required to have continuous ECG monitoring (brief interruptions are allowed due to transit and the subject's necessary activities) from 0 h to stopping the infusion of investigational product.
- 12-lead ECG, vital signs (blood pressure [BP], respiratory rate [RR], heart rate [HR], body temperature [T]), oxygen saturation (SpO_2): the investigator should perform 12-lead ECG and record the subject's vital signs (blood pressure [BP], respiratory rate [RR], heart rate [HR], body temperature [T]) and transdermal oxygen saturation (SpO2) from randomization to 0 h, and at 1 h, 2 h, 3 h, 6 h (± 5 min as the assessment point window from 1 h to 6 h), 12 h, and 24 h (± 15 min as the assessment point window for 12 h and 24 h assessments). The investigator will decide whether additional 12-lead ECG is required according to the subject's clinical condition.
- PK blood sampling: three PK blood samples will be collected from each subject for plasma concentration analysis and drug metabolizing enzyme gene analysis as specified in the *Clinical Sample Operation Manual*. The PK blood sampling time points include: immediately after the end of of loading dose infusion of investigational product, and 20 min, 45 min, 1 h, 3 h, 6 h, 12 h and 24 h after the infusion (see *Clinical Sample Operation Manual* for the specific blood sampling points for each subject).
- Record the amount of rescue analgesics parecoxib sodium for injection and sufentanil injection as appropriate;
- Concomitant medications and non-drug therapies as well as AEs will be collected.

Note: NRS pain intensity score and pain relief score may not be rated if the subject is sleeping at the corresponding assessment point.

Follow-up Period: 24 h to D4 ± 1

- Subject satisfaction score: within 30 min after stopping the PCA pump at 24 h (± 5 min), the subject will score his/her satisfaction with analgesia of the investigational product;
- Study doctor satisfaction score: within 30 min after stopping the PCA pump at 24 h (± 5 min), the investigator will score his/her satisfaction with analgesia of the investigational product;
- Complete physical examination: including overall appearance, skin and mucosa, lymph nodes, head and neck, chest, abdomen, muscles and bones, nervous system, and other parts of the body;
- Vital signs: blood pressure (BP), heart rate (HR), respiratory rate (RR), body temperature (T);
- Oxygen saturation (SpO_2) measurement;
- 12-lead ECG;
- Laboratory tests: including hematology, urinalysis, blood chemistry and coagulation function;
- Concomitant medications and non-drug therapies as well as AEs will be collected.

During the follow-up period, the subject will complete relevant examinations and complete the safety assessment according to the study flow chart.

Unscheduled Visits

If a subject experiences an AE during the trial and requires an unscheduled visit, the following items will be recorded:

- Concomitant medications/treatments
- AE follow-ups
- Relevant tests performed

Withdrawal by the Subject

1. Subjects may withdraw from the study at any time.
2. The investigator may decide to discontinue the investigational product and/or withdraw the subject from the study if any medical condition occurs prior to dosing or during the administration, which may put the subject at risk, or if the subject is unable to complete the study as specified in the protocol.

Procedures for Subject Withdrawal from the Study

Every effort must be made to complete the efficacy and safety tests specified in the protocol at the time of withdrawal from the trial, complete the safety follow-up period, and fully record AEs and outcomes. The investigator may suggest or provide new or alternative treatments to the subject according to the subject's actual conditon.

If a subject refuses to come to the study site for further visits, his/her AE and outcome should continue to be followed unless the subject withdraws consent to disclose further information or to be contacted. In such cases, no further study evaluations should be performed and no further data should be collected. The sponsor may retain and continue to use all data prior to withdrawal of informed consent by the subject.

Premature Termination or Suspension of the Study

The study may be prematurely terminated or suspended if justified. It may be due to the decision of the regulatory authorities, opinion of the Ethics Committee (EC), the efficacy or safety concerns of the investigational product, or the decision of the sponsor. In addition, Hengrui reserves the right to discontinue the development of the investigational product at any time. The party deciding to suspend/terminate the study will issue a written notice recording the reason for the discontinuation or suspension of the study to the investigator, the sponsor and the regulatory authorities. The investigator should immediately inform the Ethics Committee and the sponsor and provide relevant reasons.

End of Study Definition

The end of study is defined as:

Last subject last visit (last scheduled or unscheduled visit, whichever is later).

Evaluation

Efficacy Evaluation

- **SPID evaluation:** the investigator will instruct the subjects to assess the pain intensity (PI) at rest using the Numeric Rating Scale (NRS, 0-10 for varying degrees of pain, 0 for painlessness, larger numbers for more severe pain, and 10 for the worst pain) and obtain the PI score. The specific assessment points are: after randomization and within 5 min before the start of infusion of loading dose of investigational product (as baseline), immediately after the end of loading dose infusion, 20 min, 30 min, 45 min (± 1 min as the assessment point window from immediately after the end of loading dose infusion to 45 min), 1 h, 1.5 h, 2 h, 3 h, 4 h, 5 h, 6 h (± 5 min as the assessment point window from 1 h to 6 h), 8 h, 10 h, 12 h (± 10 min as the assessment point

window for 8 h, 10 h, and 12 h), 18 h and 24 h (± 20 min as the assessment point window for 18 h and 24 h) after the infusion. The difference from baseline PI score will be recorded as PID, calculated using the formula SPIDt=∑[PIDt* (TIMEt-TIMEt-1)](h). For example, $SPID_2=(PI_2-PI_0)\times(2-1)+(PI_1-PI_0)\times(1-0.5)+(PI_{0.5}-PI_0)\times(0.5-0)$.

- **TOTPAR evaluation:** the investigator will instruct the subject to assess the degree of pain relief (PR) using the Likert scale (0 as no response, 1 mild response, 2 moderate response, 3 significant response, 4 complete response) and obtain the PR score. The specific assessment points are: immediately after the end of of loading dose infusion and 20 min, 30 min, 45 min (± 1 min as the assessment point window from immediately after the end of loading dose infusion to 45 min), 1 h, 1.5 h, 2 h, 3 h, 4 h, 5 h, 6 h (± 5 min as the assessment point window from 1 h to 6 h), 8 h, 10 h, 12 h (± 10 min as the assessment point window for 8 h, 10 h, and 12 h), 18 h and 24 h (± 20 min as the assessment point window for 18 h and 24 h) after the infusion. TOTPAR is calculated according to the formula TOTPAR t=∑[PRt* (TIMEt-TIMEt-1)](h).
- **Evaluation of analgesia by the investigational product:** record the total number of presses and number of effective presses of PCA pump from 0 h to 24 h.
- **Evaluation of rescue analgesia:** record the time to the first use of rescue analgesics, cumulative amount of rescue analgesics from 0 h to 24 h, number of treatments of rescue analgesia from 0 h to 24 h, and percentage of subjects who do not use rescue analgesics from 0 h to 24 h.
- **The subject's satisfaction score with analgesic treatment:** the subject recalls the postoperative analgesic effect and rates the satisfaction score, ranging from 0 to 10 points, with 0 representing dissatisfied and 10 representing very satisfied.
- **The investigator's satisfaction score with analgesic treatment:** the study doctor assesses the subject's postoperative analgesic effect, ranging from 0 to 10 points, with 0 representing dissatisfied and 10 representing very satisfied.

Safety Evaluation

Pregnancy Test

For female subjects of childbearing potential, a pregnancy test (blood or urine pregnancy test) should be performed at screening. The study treatment will be given after a negative pregnancy test result is obtained at screening.

Adverse Events

Assessment of AEs includes type, incidence, severity, time of onset, seriousness, and relationship. AEs that occur during the study will be recorded on the AE page of the eCRF.

The following AEs need to be focused on and described statistically separately: nausea, vomiting, dizziness, and respiratory depression (respiratory depression is defined as respiratory rate < 8 beats/min and/or oxygen saturation < 90%).

Clinical Laboratory Evaluation

Blood and urine samples for laboratory tests will be collected according to the Clinical Study Flow Chart and will be analyzed at the local laboratory.

Table 7-1 Laboratory Tests

Hematology	Red blood cells (RBC), white blood cells (WBC), neutrophils (NEUT), hemoglobin (HB) Platelets (PLT)
Blood chemistry	Aspartate aminotransferase (AST), alanine aminotransferase (ALT), total bilirubin (TBIL), creatinine (Cr),<br>Fasting or non-fasting blood glucose (FBG or BG), urea nitrogen (BUN) or urea (Urea), potassium (K^+), sodium (Na^+)
Urinalysis	Urine white blood cell (WBC), urine red blood cell (BLD), urine glucose (U-GLU), urine protein (PRO)
Coagulation	Thrombin time (TT), partial activated thromboplastin time (APTT), prothrombin time (PT) Fibrinogen (FIB)
Infectious diseases	Hepatitis B surface antigen (HBsAg), hepatitis C antibody (HCVAb), treponema pallidum antibody,<br>Human immunodeficiency virus antibody (HIVAb)
Pregnancy test	Blood or urine pregnancy test
Drug abuse screening	Methamphetamine, dimethyldioxyamphetamine, tetrahydrocannabinol acid, ketamine, and morphine

Vital Signs, SpO_2 and Complete Physical Examination

The subjects will examine the vital signs and SpO_2, and perform a complete physical examination at the time points in the study flow chart. Vital signs should be measured after the subject rests for at least 5 minutes. Blood pressure should be measured with the dominant arm at the same height as the heart in a sitting position.

12-Lead ECG

The subjects will undergo a 12-lead ECG at the time points in the study flow chart. It is required to be performed after the subject rests quietly for at least 5 minutes. The ECG examination should at least include: heart rate, PR, QT and QTc intervals. If QTc interval increases > 30 ms from pre-dose, up to 2 ECGs will be added with at least 10 minutes apart to determine the

accuracy of the original measurement and exclude abnormal ECGs due to incorrect lead placement.

Use of antiemetics

The use of antiemetics will be recorded from the end of surgery to the end of the follow-up period.

PK Evaluation

Blood samples will be collected from subjects at the study site according to the time points in the study flow chart. The biological sample testing unit will analyze the plasma SHR8554 concentration (the test substances will be ultimately decided according to the Method Validation Report) and the genetic testing unit will analyze the gene of drug metabolizing enzymes.

Collection and processing of PK blood samples

PK blood samples will be collected according to the protocol requirements, and 3 mL of venous blood samples will be collected into K_2EDTA tubes (in case of indwelling needle, about 0.5-1 mL of blood should be drawn and discarded before each blood collection, and the tubes will be sealed with heparin sodium or normal saline after collection is completed). Centrifuge for 10 min (centrifugal force 1500 g) at 2-8 °C within 2 h to obtain clear upper plasma (if the effect of separating plasma from blood cells is not good under this condition, the centrifuge can be adjusted appropriately, but the maximum centrifugal force should not exceed 2500 g). After centrifugation, a minimum of 0.5 mL of upper plasma will be transferred into a cryogenic cryovial (test sample), and the remaining plasma will be transferred to another vial as backup sample. The remaining lower blood cell samples after plasma removal will be stored in blood collection tubes (subjects who consent genetic testing will store the lower blood cell samples at two time points). The duration from the end of centrifugation until plasma separated and frozen in a freezer should be no more than 1 h. The duration from whole blood collection until plasma separated and frozen in a freezer should be no more than 2 h. The test plasma samples, backup plasma samples and lower blood cell samples will be stored upright in a freezer below -60 °C, until transport. See *Clinical Sample Operation Manual* for the specific operation.

Each sample has a unique sample number. The test plasma samples will be stored on dry ice and sent to the sample analysis laboratory, while the backup plasma samples will be stored at the clinical unit or at the sponsor-designated unit. Lower blood cell samples will be stored on dry ice and sent to the genetic testing unit.

The actual date and time of blood sample collection and the exact time of treatment will be recorded in the PK blood sample collection page of the study medical record and eCRF.

Problems encountered in the collection of blood samples will be noted in the study medical record and eCRF.

Adverse Events Reporting

Adverse Events (AEs)

Definitions of Adverse Events

Adverse event (AE) is defined as any untoward medical condition that occurs after a subject receives the drug, which may manifest as symptoms, signs, diseases, or laboratory abnormalities, but does not necessarily have a causal relationship with the investigational product. AEs include, but are not limited to, the following:

1) Worsening of pre-existing (before enrollment) medical conditions/diseases (including worsening of symptoms, signs, laboratory abnormalities);

2) Any new AEs: any new adverse medical conditions (including symptoms, signs, newly diagnosed diseases);

3) Abnormal and clinically significant laboratory values or results.

The study personnel should record in detail any AE, including: AE name, time of onset, severity, relationship to the investigational product, end time, action taken with the investigational product, and final results and outcome.

AE information will be collected from the time the subject signs the ICF until the end of the safety follow-up period. Physical conditions (including laboratory abnormalities) that are already present at the time of the subject's screening examination (including those newly identified at the time of the screening examination) will be recorded as pre-existing or concomitant diseases and will not be recorded as AEs. However, if a pre-existing or concomitant condition worsens during the study, an AE should be recorded.

Surgeries/treatments scheduled prior to informed consent but to be conducted during the study, or planned hospitalizations, or surgery-related events (e.g., post-operative diagnoses [including polyps, inflammation, tumors, etc.], biopsy results, etc.) will not be recorded as AE.

Adverse Event Severity and Grading Criteria

Mild, moderate, and severe will be used to describe the extent of adverse events. The severity will be graded according to the following uniform criteria:

- Grade 1 (mild): usually transient, generally does not affect activities of daily living, does not require medical treatment, or may require minimal treatment;
- Grade 2 (moderate): the subject feels unwell, his/her activities of daily living are affected, and usually requires treatment to mitigate, but there is no risk of major or permanent harm to the

subject;

- Grade 3 (severe): the subject's activities of daily living are interrupted, or the clinical condition is seriously affected, and centralized treatment and intervention are required;

Note that the severity and seriousness of AEs should be differentiated. Severe is only used to describe the extent, for example, headache may be severe in intensity, but cannot be listed as a serious adverse event (SAE) unless it meets the SAE criteria as described in **Section 8.2.1**.

Relationship of Adverse Events to the Investigational Product

Any untoward reactions or abnormal changes in objective laboratory indicators reported by the subject during treatment should be truthfully recorded, and the AE manifestations, severity, duration, treatment and outcome should be indicated. The investigator should comprehensively determine the relationship between the AE and the investigational product, such as whether the AE has a reasonable temporal relationship with the treatment, characteristics of the investigational drug, the toxicological and pharmacological effects of the investigational drug, whether the subject uses other concomitant drugs, the subject's underlying disease, medical history, family history, challenge and rechallenge reactions, etc. The possible relationship between AEs and the investigational product will be assessed according to the five-level classification of "related, possibly related, unlikely related, unrelated, and not determined".

Serious Adverse Events (SAEs)

Definition of SAE

Serious adverse event (SAE) is an event that occurs during the clinical trial that requires hospitalization or prolonged hospitalization, leads to disability or incapacity, is life-threatening or fatal, or results in congenital malformations. SAEs include the following medical events:

- Fatal;
- Life-threatening: defined as the subject is at the risk of death at the time of the event rather than assuming a possible death if the condition worsens;
- Requires hospitalization or prolonged hospitalization;
- Leading to permanent or serious disability/incapacity;
- Resulting in congenital anomalies/birth defects;
- Other medically significant events (defined as adverse events that may not immediately result in death, be life-threatening, or require hospitalization, but may jeopardize the subject or require intervention to prevent any of the above outcomes. For example, allergic bronchospasm requiring special treatment in an emergency room or at home, dyscrasia or convulsions that do not result in hospitalization, or development of drug dependence or drug abuse).

Hospitalization

AEs in clinical studies that lead to hospitalization (even if less than 24 hours) or prolonged hospitalization should be considered as SAEs.

Hospitalization does not include the following:

- Hospitalization for this abdominal operation
- Admission to rehabilitation facilities
- Admission to nursing homes
- Admission to routine emergency room
- Day surgery (such as outpatient/day/ambulatory surgery)

Any hospitalization or prolonged hospitalization due to any causes other than AE deterioration will not be considered as a SAE. For example, the following reasons are not considered as SAEs:

- Hospitalization for a pre-existing disease that does not worsen without any new AEs (e.g., hospitalization due to laboratory abnormalities that occur prior to the study and still persist);
- Hospitalization due to administrative reasons (e.g., annual routine physical examination);
- On-study hospitalization as specified in the protocol (e.g., protocol-specified procedures);
- Elective hospitalization for any cause other than worsened AEs (e.g., elective surgery);
- Pre-scheduled treatment or surgical procedures (recorded in the protocol and/or in the subject's individual baseline data);
- Hospitalization only for use of blood products.

Any diagnostic or therapeutic invasive (e.g., surgery) or non-invasive procedure should not be reported as an AE. However, the condition that results in such procedures should be reported as an AE if it meets the criteria of AE. For example, acute appendicitis that occurs during the AE reporting period should be reported as an AE, while the appendectomy thus performed should be recorded as the treatment of the AE.

Pregnancy

If a female subject becomes pregnant during the clinical study, the subject will be discharged from the study; if the partner of a male subject becomes pregnant during the clinical study, the subject will continue the clinical study. The investigator should fill the Hengrui Clinical Study Pregnancy Report/Follow-up Form and report it to the sponsor within 24 hours after becoming aware of the pregnancy event.

The investigator should follow up the pregnancy event to the final outcome (including any

premature termination of pregnancy or delivery), and follow up the delivery until 1 month after delivery. The pregnancy results should be reported to the sponsor. If the pregnancy results meet the criteria for an SAE (eg, extrauterine pregnancy, spontaneous abortion, intrauterine fetal death, death neonatal, or congenital anomaly, etc.), then it should be reported following the procedures of SAE reporting.

If a subject experiences an SAE during pregnancy, the SAE reporting procedure should also be followed.

Reporting Procedures

Serious Adverse Event Reporting

The collection period of SAEs should start from signing the ICF until the end of the safety follow-up period. In the event of an SAE, whether the initial report or the follow-up report, the investigator must immediately fill in the Clinical Study Serious Adverse Event/Adverse Event of Special Interest Report Form, sign and date, notify the sponsor immediately within 24 hours after becoming aware of it, and promptly report it to relevant institutions in accordance with local regulatory requirements.

SAEs that occur after the safety follow-up period but are suspected to be related to the investigational product should be collected. For SAEs, the symptoms, severity, relationship to the investigational product, time of occurrence, time of management, measures taken, follow-up time and manner, and outcome should be recorded in detail. If the investigator thinks that an SAE is unrelated to the investigational product but potentially related to the study conditions (e.g., termination of the pre-existing treatment, or complications during the trial), it should be described in the narrative section of the SAE report. If the severity of an ongoing SAE or its relationship to the investigational product is changed, a follow-up report should be submitted immediately. False information included in the previous SAE report per the investigator may be corrected, revoked or downgraded in the follow-up report, and reported in accordance with the SAE reporting procedures.

The sponsor of this project receives SAE (and pregnancy) reports at: hengrui_drug_safety@hrglobe.cn.

Follow-up of AEs/SAEs/Pregnancies

At each study visit, the investigator should assess whether the subject experiences an AE. All AEs/SAEs should be followed until the event disappears, resolves to baseline, stabilizes, or is reasonably explained (eg, lost to follow-up or death). Every effort should be made to ensure that the subject gets the best outcome and a clear causality assessment. The principles of collection and follow-up period of AEs/SAEs occurring in the study can be found in Table 8-1.

Table 8-1 Principles of AE/SAE/Pregnancy Collection and Follow-up Period

Interval	Collection Requirements
Signing the ICF to end of safety follow-up period	All AEs/SAEs<br>* Pregnancy events (start the collection after the first dose of investigational product)
After the safety follow-up period	Drug-related SAEs

Clinical Monitoring

Clinical monitoring of the study site is required to ensure that the subject's rights and interests are protected, the reported study data are accurate, complete and reliable, and the study is conducted in accordance with the currently approved protocol/amended protocol, Chinese GCP, and applicable regulatory requirements.

Data Analysis/Statistical Method

Sample Size

With reference to the results of the foreign study TRV130-3002, assuming that the primary efficacy variable $SPID_{24}$ in the SHR8554 0.75 mg group in this study is comparable to the efficacy in the 0.35 mg group in the TRV130-3002 study, and conservatively assuming that the mean (standard deviation) of $SPID_{24}$ in the low dose group of this study is 88 (45), and the mean (standard deviation) of $SPID_{24}$ in the placebo group is 70 (40), it's estimated to need 118 subjects in each group by the sample size calculation formula (PASS) of comparing the means of two samples to achieve the power of 90% with $\alpha = 0.05$ (two-sided), provided that the four groups are designed in a 1:1:1:1 ratio. Considering the dropout rate of 10%, each group is planned to enroll 132 subjects, with a total of 528 subjects.

Statistical Analysis Plan

The detailed methods for summarization and statistical analysis of the data collected for this study will be included in the SAP which will be finalized and filed prior to database lock. If a change is made to the protocol that will have an important impact on the SAP per the sponsor or the principal investigator and statistician, the SAP should be revised to be consistent with the protocol.

Statistical Hypotheses and Discriminant Rules

The primary efficacy variable of this trial is $SPID_{24}$, and the difference test will be used for the group comparison between the experimental group and the placebo group.

Hypothesis:

H_0: $\mu_T = \mu_C$

H_1: $\mu_T \neq \mu_C$

α level: 0.05 (two-sided).

In this study, the high and low dose groups will be tested sequentially to ensure that the Type I error rate α is controlled within 0.05 (two-sided). The test between the high dose group and the placebo group will be performed first with α of 0.05 (two-sided). If there is a significant difference between the high dose group and the placebo group, then the test between the low dose group and the placebo group will be performed with α of 0.05 (two-sided); if there is no significant difference between the high dose group and the placebo group, only exploratory analysis will be performed for the comparison of the low dose group and the placebo group. For the morphine group, only descriptive statistics will be applied.

Analysis Populations

- Full analysis set (FAS): including all subjects who received at least one dose of study drug after randomization in accordance with the intent-to-treat (ITT) principle.
- Per-protocol set (PPS): it is a subset of the FAS and includes the subjects who did not have major protocol deviations in the FAS.
- Safety set (SS): including all enrolled subjects who received the study drug.
- PK analysis set (PKAS): all enrolled subjects who received the study drug and have at least 1 valid plasma concentration measurement during the study. The subjects will not be included in the PK analysis set in case of: a) major protocol violations which affect the subject's PK characteristics; and b) use of concomitant medications which have an impact on PK parameters.

Statistical Methods

Basic Method

The general principles for descriptive statistical analysis in this study are as follows:

Count data will be summarized using frequencies and percentages. Measurement data will be summarized using mean, standard deviation, median, maximum and minimum. Plasma concentration data will be summarized using mean or geometric mean, standard deviation, coefficient of variation or geometric coefficient of variation, median, maximum, and minimum.

Efficacy Analysis

1) Time-weighted Sum of Pain Intensity Differences at rest at 6 h, 12 h, 18 h, 12h-24 h, and 24 h ($SPID_6$, $SPID_{12}$, $SPID_{18}$, $SPID_{12-24}$ and $SPID_{24}$) will be compared between groups using analysis of variance;

2) Time-weighted Total Pain Relief at 6 h, 12 h, 18 h, 24 h, and 12-24 h ($TOTPAR_6$, $TOTPAR_{12}$, $TOTPAR_{18}$, $TOTPAR_{24}$, and $TOTPAR_{12-24}$) will be compared between groups using analysis of variance;

3) The time to the first use of rescue analgesics will be compared between groups by nonparametric test;

4) Cumulative amount of rescue analgesics from 0 h to 24 h will be compared between groups using analysis of variance;

5) The number of treatments of rescue analgesia from 0 h to 24 h, the total number of presses and number of effective presses of PCA pump from 0 h to 24 h will be compared between groups by nonparametric test;

6) The percentage of subjects who do not use rescue analgesics from 0 h to 24 h will be compared between groups using chi-square test or Fisher's exact probability method;

7) The subject's satisfaction score with analgesic treatment will be compared between groups by nonparametric test;

8) The investigator's satisfaction score with analgesic treatment will be compared between groups by nonparametric test.

Handling of Missing Data

The method of imputing missing values is provided in the Statistical Analysis Plan (SAP).

Safety Analysis

Safety analysis will be tabulated and analyzed by descriptive statistics based on the Safety Set (SS), including AEs, laboratory tests, vital signs, electrocardiogram data, etc.

AEs will be coded according to MedDRA. AEs will be summarized by treatment group. The incidences of AEs, drug-related ADRs, SAEs, etc. will be summarized by system organ class (SOC) and preferred term (PT), and relevant adverse events will be tabulated. Incidences (number of subjects: number of subjects with at least one AE) will be calculated by SOC and PT; if a subject experiences the same AE for many times, the AE with the highest severity will be counted.

Other safety variables will be summarized using descriptive statistics. The mean, standard deviation, maximum, minimum and median will be used to describe each measurement and change from baseline. The frequency and percentage will be used to describe categorical

variables.

PK Data Analysis

The population PK model parameters (CL, V, etc.) will be estimated using the pharmacometric method.

The specific analytical methods are detailed in the Pharmacometric Analysis Plan. The analysis software is NONMEM (Version 7.4 or higher).

Interim Analysis

Not applicable.

Data Management Methods

An electronic data capture (EDC) system will be used for the data collection and management in this study.

Data Collection

Completion of Electronic Case Report Form (eCRF)

The eCRFs will be completed by the investigator or data entry personnel through the EDC system. The eCRFs should be completed timely and it should be ensured that the entered data can be traced to the original records. When the data is modified in EDC, the reason for data modification should be filled in according to the system prompts. Modification to CV and its reason will be recorded in the audit trail of the EDC system. The investigator or his/her authorized person will confirm the authenticity, completeness, and timeliness of the eCRF data and electronically sign in the EDC system.

Use of Electronic Data Capture (EDC) System

The data manager builds eCRFs and edit check procedures in the EDC system according to the protocol, finishes the user acceptance testing and makes it ready for online use prior to enrollment of the first subject. All EDC users must complete relevant training with the training records archived, and then gains access to the eCRFs for this study. Before the user signs an electronic signature on the eCRF, a statement of use of electronic signature needs to be confirmed and agreed upon. The account is restricted to the user's own use, and the password needs to be properly maintained and changed regularly. If there is any personnel change in the study team, the authorization needs to be revoked timely.

Data Management

eCRF Data Review

The edit check procedure of the EDC system will check the completeness and logic of entered data and inquire about the data that may have problems. The investigator or data entry personnel

may make corrections to the data, or explain and confirm the data by answering queries. The monitor, data manager, and medical reviewer will also review the eCRF data and inquire about the data in question as appropriate. The investigator should promptly respond to the queries from the system and data reviewers who may issue queries several times until the question is resolved.

Data Review Meeting and Database Lock

Prior to database lock, the study team needs to complete data cleaning, summarize all protocol deviations that occur during the conduct of the trial, and convene a data review meeting to determine the analysis population. Decisions made during the data review meeting will be documented. After being adopted by the data review meeting and confirmed by the study team, the study database in the EDC system will be locked and the data will not be changed after the lock.

Data Archiving

After completion of the study, the EDC system will generate the subject eCRFs in PDF format and submit them to the sponsor and relevant institutions for archiving, respectively, in case of audit in the future. The study data will be stored and managed in accordance with China GCP requirements, and the investigator should notify the sponsor in advance before destroying any study-related documents or records. After the investigational product is approved for marketing, or even if the clinical study is prematurely terminated, the sponsor should keep the clinical trial data for at least 5 years.

Source Data and Source Documents

According to ICH E6, relevant regulations and the requirements of testing facility for the protection of individual information, each study site must properly keep the records of treatment and scientific research related to this study. As part of the study Hengrui sponsor or participate in, each study site should allow representatives and regulatory authorities authorized by Hengrui to inspect or, if permitted by the law, reproduce the clinical records for quality review and audits, as well as evaluation of safety, progress and data validity.

Source data is necessary for the reconstruction and evaluation of the clinical study, and is the original record of clinical findings, observations or other activities. Examples of such original documents and data records include, but are not limited to, hospital records, laboratory records, memos, pharmacy dispensing records, sound records of counseling sessions, recorded data from automated instruments, copied or transcribed records that are verified as accurate and complete, microfiche, photographic plate, microfilm strip or disk, X-ray films and documents and records

of subjects retained at the pharmacies, laboratories and medico-technical departments involved in the clinical trial.

Quality Assurance and Quality Control

In order to ensure the quality of the trial, the sponsor and the investigator will jointly discuss and develop a clinical study plan before the official start of the trial. Relevant study personnel should have GCP training.

Each study site must follow the SOP for management of the investigational products, including receiving, storage, dispensing, recovery and destruction (if applicable).

In accordance with the GCP guidelines, necessary measures should be taken in the design and implementation stages of the study to ensure that data collected are accurate, consistent, complete and reliable. All observations and abnormal findings in the clinical study should be carefully verified and documented in a timely manner to ensure the reliability of the data. The instruments, equipment, reagents and reference standards etc., used for various examinations in the clinical study should follow rigorous specifications and work normally.

Information required by the protocol should be entered into eCRF by the investigator. The monitor should verify the integrity and accuracy of the entered information, and instruct the study site personnel to make necessary modifications and additions.

Drug regulatory authorities, IRB/IEC, and monitors and/or auditors of the sponsor may conduct systematic checks on study-related activities and documents to evaluate whether the study is performed in accordance with the protocol, SOPs and applicable laws and regulations, and whether the study data is recorded timely, truthfully, accurately and completely. The audits should be performed by personnel who are not directly involved in the clinical study.

Regulations, Ethics, Informed Consent and Subject Protection

Regulatory Considerations

The study is designed based on the following laws and regulations:

1) *Drug Registration Regulation* (2020 Version)
2) *Good Clinical Practice (GCP)* (2020 Version)
3) *Technical Guidelines for Clinical Pharmacokinetic Studies of Chemical Drugs* (2019 Version)
4) Consensus on international ethical guidelines, including the *Declaration of Helsinki* and international ethical guidelines of the Council for International Organizations of Medical Sciences (CIOMS)
5) ICH Guidelines

6) Other applicable laws and regulations

Ethics

This study protocol needs to be reviewed and approved in writing by the Ethics Committee of the hospital before implementation. The EC should be provided with the protocol, protocol amendments, ICF and other relevant documents, such as recruitment advertisements. This clinical study must comply with the *Declaration of Helsinki,* GCP promulgated by NMPA, and relevant laws and regulations. Approval must be obtained from the EC of the hospital prior to initiation of the study.

Without the consent of both the sponsor and the investigator, neither party may modify the protocol. The investigator may change or deviate from the study protocol prior to the approval of the Ethics Committee only in order to eliminate direct or immediate harm to subjects. Meanwhile, the deviations or changes and their causes, as well as the proposed amendments to the protocol should be submitted to the EC for review and approval as soon as possible. The investigator must explain and record any protocol deviation.

During the clinical study, any modifications made to the protocol should be submitted to the Ethics Committee, and, if necessary, other study documents should be revised accordingly at the same time, and submitted for approval according to the requirement of the Ethics Committee. It is the responsibility of the investigator to notify the Ethics Committee of the end of the trial in accordance with the relevant requirements of the Ethics Committee.

Ethics Committee

The protocol, ICF, recruitment materials, and all subjects' materials will be submitted to the Ethics Committee for review and approval. Subjects may not be enrolled until the protocol and ICF are approved. Any amendments to the protocol may not be implemented until they have been reviewed and approved by the Ethics Committee. All revisions to the ICF must also be approved by the Ethics Committee. Whether the subjects who have signed the previous ICF need to sign the new version of ICF will be determined by the Ethics Committee.

Informed Consent

ICF

The ICF includes all elements specified in ICH, Chinese GCP, and regulatory requirements and complies with the ethical principles set forth in the *Declaration of Helsinki*. Study treatment, procedures, risks of participating in the study will be described in the ICF.

Confidentiality of subjects' identification should be described in the ICF. Medical representatives of Hengrui and regulatory authorities have access to the subjects' data.

Informed Consent Process and Records

Informed consent begins before an individual's consent to participate in a clinical study and continues throughout the entire course of the clinical study. The risks and potential benefits of participating in the study will be discussed in detail and adequately with the subject or his/her legal representative. Subjects will be required to read and understand the ICF approved by the Ethics Committee. The investigator will explain the clinical study to subjects and answer any questions that subjects may ask. Subjects are only allowed to participate in the study after signing the ICF. During the study, the subjects may withdraw from the study at any time without any reason. The ICF will be provided in duplicate, one of which will be retained by the subject. Even if the candidate subjects refuse to participate in this study, their rights and interests will be fully protected and the quality of their medical care will not be affected in any way.

Confidentiality of Subject Information

The subjects' study data will be strictly kept confidential by the investigator, study participants, the sponsor and its agents. Confidentiality also covers biological samples, in addition to the subject's clinical information. The study protocol, files, data, and all other information generated therefrom will be kept strictly confidential. No relevant study or data information shall be disclosed to any unauthorized third party without the prior written approval of the sponsor.

Other authorized representatives of the sponsor, IEC or regulatory authorities, representatives of pharmaceutical companies who provide the study drugs can check all documents and records that the investigator is required to maintain, including but are not limited to medical records and treatment records. The study site should allow access to these records.

The contact information of the subjects will be securely stored at each study site and will only be used internally in the course of the study. At the end of the study, all records will continue to be kept in a safe place according to the time limit set by the local IRB and regulations.

Publication of Study Results

Intellectual property rights, data, and results related to this study are the property of the sponsor. All data and results generated from this study are the property of Jiangsu Hengrui Pharmaceuticals Co., Ltd. and its subsidiaries. If the investigator plans to publish any study-related data and information, the investigator should provide Hengrui with the full texts of all planned publications (posters, invited lectures, or guest lectures) at least 30 days prior to submission for publication or other forms of release. If patents need to be applied to protect intellectual property, the investigator should agree to postpone the release for no more than 60

days. Prior to public publication, the sponsor may request the investigator to delete any previously unpublished confidential information (other than the study results). If the study is part of a multicenter study, the investigator must agree that the first publication is the integrated result of all sites. However, if a manuscript including the integrated analysis has not been submitted for publication within 12 months after all the study sites have completed the study or after the termination of the study, the investigator may publish the results of the individual site in accordance with other requirements in this section.

References

1. Anesthesiology Branch of the Chinese Medical Association. *Expert consensus on postoperative pain management in adults.* (2017).
2. Scott M. DeWire, et al., A G Protein-Biased Ligand at the μ-Opioid Receptor Is Potently Analgesic with Reduced Gastrointestinal and Respiratory Dysfunction Compared with Morphine. J Pharmacol Exp Ther. 2013 Mar; 344:708-717.
3. Soergel DG., et al., First clinical experience with TRV130: pharmacokinetics and pharmacodynamics in healthy volunteers. J Clin Pharmacol. 2014 Mar; 54(3):351-7.
4. Soergel DG., et al., Biased agonism of the μ-opioid receptor by TRV130 increases analgesia and reduces on-target adverse effects versus morphine: A randomized, double-blind, placebo-controlled, crossover study in healthy volunteers. Pain. 2014 Sep; 155(9):1829-35.
5. *Key* Points *to Consider in Clinical Trials for Analgesic Drug Registration in China*.

Appendix

Appendix 1. NRS Pain Score at Rest

Numeric Rating Scale (NRS): NRS uses 0-10 for varying degrees of pain, 0 for painless, and 10 for severe pain. Pain intensity grading criteria are: 0: painless; 1-3: mild pain; 4-6: moderate pain; 7-10: severe pain. The subjects use the 11 numbers from 0 to 10 to describe the pain intensity at rest after surgery. The greater the number, the more severe the pain.

The investigator should ask the subject about the degree of pain and mark "√" below the corresponding number.

0	1	2	3	4	5	6	7	8	9	10

Appendix 2. Subject Satisfaction Score

The subject recalls the postoperative analgesic effect and rates the satisfaction score, ranging from 0 to 10 points, with 0 representing dissatisfied and 10 representing very satisfied. Mark "√" below the corresponding number.

0	1	2	3	4	5	6	7	8	9	10

Appendix 3. Investigator Satisfaction Score

The study doctor assesses the subject's postoperative analgesic effect, ranging from 0 to 10 points, with 0 representing dissatisfied and 10 representing very satisfied. Mark "√" below the corresponding number.

0	1	2	3	4	5	6	7	8	9	10

Appendix 4. Subject Pain Relief Score (Likert Scale)

Likert scale: 0-4 represent different degrees of response, 0 as no response, 1 mild response, 2 moderate response, 3 significant response, 4 complete response.

The investigator should ask the patient about pain relief, and mark "√" below the corresponding number.

0	1	2	3	4

Appendix 5. ASA Score

The American Society of Anesthesiologists (ASA) classifies patients into 5 levels before anesthesia according to their physical condition and surgical risk:

Grade I: normal and healthy. No systemic disease other than local lesions.

Grade II: mild or moderate systemic disease.

Grade III: severe systemic disease, limited daily activities, but not incapacitated to work.

Grade IV: severe systemic disease, incapacity to work, life-threatening.

Grade V: critically ill, life-threatening, moribund patient. In case of emergency surgery, mark "urgent" or "E" in front of one of the above grades.

In patients of grade I and II, anesthesia and operation are well tolerated, and the anesthesia goes well. Grade III patients have certain risks in anesthesia. Adequate preparations are required before anesthesia. Effective measures should be taken to prevent the possible complications in anesthesia. Grade IV patients have very great risks in anesthesia. The condition of grade V patients is very serious. Their anesthetic tolerance is very poor. There is a threat of death at any time. Anesthesia and surgery are exceptionally dangerous. The preparations before anesthesia are very important, so they must be adequate, meticulous and considerate.

Appendix 6. Fridericia's Formula

Calculated as QTc = QT/(RR ^ 0.33). RR is the normalized heart rate value, calculated as 60 divided by heart rate.

Efficacy and Safety of SHR8554 Injection for Postoperative Analgesia after Abdominal Surgery: A Multicenter, Randomized, Double-Blind, Placebo/Active-Controlled Phase 3 Clinical Trial

Statistical Analysis Plan

(V 1.0)

Sponsor: Jiangsu Hengrui Pharmaceuticals Co., Ltd.

Statistics Institution: Department of Biostatistics, School of Public HealthNanjing Medical University

Sept-2021

Statistical Analysis Plan

(Version 1.0)

Investigational Product:	SHR8554 Injection
Study Title:	Efficacy and Safety of SHR8554 Injection for Postoperative Analgesia after Abdominal Surgery: A Multicenter, Randomized, Double-Blind, Placebo/Active-Controlled Phase 3 Clinical Trial
Clinical Phase:	Phase 3
Sponsor:	Jiangsu Hengrui Pharmaceuticals Co., Ltd
Principal Investigator:	Professor Xiangdong Chen, Union Hospital, Tongji Medical College, Huazhong University of Science and Technology
Version Date:	Version: 3.0; Version Date: 13 Apr 2021

Department of Biostatistics, School of Public Health, Nanjing Medical University

Version History

**SAP Version, Date**	**Protocol Version, Date**	**Written/Reviewed by**	**Revision History**
V0.1，12 Apr 2021	V2.0，20 Dec 2020	Tianyi Zhu/Donghua Lou	
V0.2，11 May 2021	V3.0，13 Apr 2021	Tianyi Zhu/Donghua Lou	Updated per protocol version 3.0
V0.2，09 Sept 2021	V3.0，13 Apr 2021	Tianyi Zhu/Donghua Lou	1. The pairwise comparison of quantitative indicators was changed to group t-test;<br>2. Added sensitivity analysis;<br>3. Adverse reactions were updated to "Treatment Related Adverse Events";<br>4. Added analysis of drug metabolizing enzymes in subjects.
V1.0，13 Sept 2021	V3.0，13 Apr 2021	Tianyi Zhu/Donghua Lou	Version 1.0 finalization

Statistical Analysis Plan Signature Page

Study Title: Efficacy and Safety of SHR8554 Injection for Postoperative Analgesia after Abdominal Surgery: A Multicenter, Randomized, Double-Blind, Placebo/Active-Controlled Phase 3 Clinical Trial

Sponsor: Jiangsu Hengrui Pharmaceuticals Co., Ltd.

Signature __________________ Date __________________

Statistical Institution: Department of Biostatistics, School of Public Health, Nanjing Medical University

Written by __________________ Date __________________

Reviewed by _______________ Date _________________

TABLE OF CONTENTS

1. Abbreviations 1
2. Brief Description of the Protocol 1
  2.1 Study Objective 1
  2.2 Study Subjects 1
  2.3 Study Design 1
  2.4 Sample Size 4
  2.5 Method of Randomization 5
  2.6 Blinded Design 5
  2.7 Interim Analysis 5
  2.8 Modifications to the Statistical Analysis in the Protocol 5
3. Efficacy Variables 5
  3.1 Primary Efficacy Variable 5
  3.2 Secondary Efficacy Variables 6
4. Safety Variables 6
  4.1 Adverse Events 6
  4.2 Treatment-related Adverse Events 7
  4.3 Serious Adverse Events 7
  4.4 Vital Signs, SpO_2 and Physical Examination 8
  4.5 Laboratory Tests 8
  4.6 12-Lead ECG 8
5. Other Variables 8
6. Analysis Set 8
  6.1 Full Analysis Set (FAS) 9
  6.2 Per Protocol Set (PPS) 9
  6.3 Safety Set (SS) 9
7. Statistical Analysis 9
  7.1 General Principles 9
    7.1.1 Test Level 9
    7.1.2 Hypothesis Testing 10
    7.1.3 Estimation of Missing Values 10
    7.1.4 Data Conventions 11
  7.2 Study Population 13
    7.2.1 Case Distribution 13
    7.2.2 Protocol Violations 13
    7.2.3 Analysis of Subject Characteristics 14
  7.3 Efficacy Evaluation 15
    7.3.1 Analysis of Primary Efficacy Variable 15
    7.3.2 Analysis of Secondary Efficacy Variables 15
  7.4 Safety Evaluation 17
    7.4.1 Drug Exposure 17
    7.4.2 Adverse Events 17
    7.4.3 Vital Signs 18

7.4.4 Laboratory Measurements 18
7.4.5 Electrocardiogram 18
7.4.6 Physical Examination 19
7.4.7 Transdermal Oxygen Saturation (SpO_2) 19
7.4.8 Concomitant Medications and Concomitant Non-drug Therapies 19
7.5 Sensitivity Analysis 20
7.6 Subgroup Analysis 20
7.7 Other Analysis 20
8. Statistical Analysis Software 20
9. References 20
10. Appendixes 20

1. Abbreviations

AE	Adverse Event
CI	Confidence Interval
FAS	Full Analysis Set
ITT	Intention to Treat
Max	Maximum
Mean	Mean
MedDRA	Medical Dictionary for Regulatory Activities
Median	Median
Min	Minimum
PPS	Per-Protocol Set
PT	Preferred Term
Q_1	25th percentile
Q_3	75th percentile
SAE	Serious Adverse Event
SOC	System Organ Class
Std	Standard Deviation
SS	Safety Set
TEAE	Treatment-Emergent Adverse Event
TRAE	Treatment Related Adverse Event

2. Brief Description of the Protocol

2.1 Study Objective

To evaluate the efficacy and safety of SHR8554 Injection for analgesia after abdominal surgery.

2.2 Study Subjects

Subjects who electively undergo abdominal surgery (laparotomy or laparoscopy) under general anesthesia and have an NRS ≥ 4 points within 4 h after the surgery.

2.3 Study Design

The study will use a multicenter, randomized, double-blind, placebo/active-controlled trial

design.

The study process consists of a screening period, a treatment period, and a follow-up period.

Screening period: from signing the ICF to randomization, with a maximum of no more than 7 days, and the day of randomization will be recorded as D1.

The subject will sign the informed consent form and complete the screening according to the study flow chart. The subject will undergo abdominal surgery (laparotomy or laparoscopy) under general anesthesia (total intravenous anesthesia or intravenous-inhalation combined anesthesia). Sevoflurane is selected as inhalation anesthetics, propofol injection is selected as intravenous anesthetics, and the dose of general anesthetics will be determined on an individual basis. Sufentanil injection (0.2-0.6 μg/kg) is selected as opioid analgesics during the anesthetic induction period. Remifentanil injection (0.1-0.5 μg/kg/min) is selected as opioid analgesics during the anesthetic maintenance period, sufentanil injection (0.1-0.2 μg/kg/time) can be added, and the doses can be adjusted appropriately according to the needs of surgery and the subject's individual conditions. The type and dose of muscle relaxants and other anesthetic adjuvant drugs are at the discretion of the anesthesiologist and/or surgeon. 2.5 μg sufentanil will be intravenously injected immediately (± 5 min) after the surgery (the last suture is completed) to prevent postoperative breakthrough pain.

Within 4 hours after the surgery, the investigator will instruct the subjects to assess the pain intensity (PI) at rest using the Numeric Rating Scale (NRS, 0-10 for varying degrees of pain, 0 for painlessness, larger numbers for more severe pain, and 10 for the worst pain), and it is recommended that the interval between the two assessments should not exceed 30 minutes. The subjects with NRS score ≥ 4 at rest at any time within 4 hours after the surgery can be randomized (it is recommended to be randomized within 10 min after the NRS score reaches 4 or more for the first time). The subjects will be randomized in a 1:1:1:1 ratio to one of the following groups: SHR8554 Injection 0.75 mg group, SHR8554 Injection 1.0 mg group, morphine hydrochloride injection group, and placebo group.

Treatment period: from randomization to 24 h, with the time to start infusion of the loading dose of investigational product recorded as 0 h.

After randomization, the investigator should infuse the investigational product to the subject as soon as possible (within 15 min after the randomization). The loading dose of the investigational product will be 5 mL, administered by an infusion pump, and the intravenous infusion time will be approximately 10 min. The Patient Controlled Analgesia (PCA) pump containing the investigational product can be initiated approximately 30 min after the start of infusion of the loading dose. The subject can receive 1 mL of the investigational product at a single press of PCA pump, and the minimum interval between two adjacent effective presses is 10 minutes. The subject will continue using the PCA pump until 24 h (± 5 min).

After the end of of loading dose infusion, rescue analgesia may be given if the investigator determines that the investigational product cannot meet the subject's analgesic needs (NRS ≥ 4 points). The first choice of rescue analgesics is parecoxib sodium for injection (20 mg for the first intravenous injection, followed by additional doses as needed, with a total dose of not more than 80 mg over 24 h), and sufentanil injection (2.5 μg for a single intravenous injection) can be used after reaching the maximum cumulative dose of parecoxib sodium for injection.

After randomization, the investigator will instruct the subject to assess the NRS score at rest within 5 minutes prior to the start of infusion of loading dose as baseline. The investigator will instruct the subject to assess the NRS scores at rest immediately after the infusion and 20 min, 30 min, 45 min (± 1 min as the assessment point window from immediately after the infusion to 45 min), 1 h, 1.5 h, 2 h, 3 h, 4 h, 5 h, 6 h (± 5 min as the assessment point window from 1 h to 6 h), 8 h, 10 h, 12 h (± 10 min as the assessment point window for 8 h, 10 h, and 12 h), 18 h, and 24 h (± 20 min as the assessment point window for 18 h and 24 h) after the infusion. At the same time as each assessment of NRS score, the investigator will instruct the subject to assess the degree of pain relief (PR) using the Likert scale (0 as no response, 1 mild response, 2 moderate response, 3 significant response, 4 complete response).

The subject will be required to have continuous ECG monitoring (brief interruptions are allowed due to transit and the subject's necessary activities) from 0 h to stopping the

infusion of investigational product.

The investigator should perform 12-lead ECG and record the subject's vital signs (blood pressure [BP], respiratory rate [RR], heart rate [HR], body temperature [T]) and transdermal oxygen saturation (SpO2) from randomization to 0 h, 1 h, 2 h, 3 h, 6 h (± 5 min as the assessment point window from 1 h to 6 h), 12 h, and 24 h (± 15 min as the assessment point window for 12 h and 24 h assessments). The investigator will decide whether additional 12-lead ECG is required according to the subject's clinical condition.

During the treatment period, PK blood samples will be collected for plasma concentration analysis and genetic analysis of drug metabolizing enzymes CYP2D6, CYP3A4 and CYP3A5. The time to the first use of rescue analgesics, the cumulative amount of rescue analgesics from the end of the infusion of loading dose to 24 h, the number of treatments of rescue analgesics, the total number of PCA pump presses within 24 h, the number of effective presses of PCA pump, and the time of effective presses of PCA pump will be recorded. Within 30 min after stopping the PCA pump at 24 h (± 5 min), the subject and the investigator will score their satisfaction with analgesia of the investigational product, respectively.

Follow-up period: 24 h to D4 ± 1.

During the follow-up period, the subject will complete relevant examinations and complete the safety assessment according to the study flow chart.

2.4 Sample Size

With reference to the results of the foreign study TRV130-3002, assuming that the primary efficacy variable $SPID_{24}$ in the SHR8554 0.75 mg group in this study is comparable to the efficacy in the 0.35 mg group in the TRV130-3002 study, and conservatively assuming that the mean (standard deviation) of $SPID_{24}$ in the low dose group of this study is 88 (45), and the mean (standard deviation) of $SPID_{24}$ in the placebo group is 70 (40), it's estimated to need 118 subjects in each group by the sample size calculation formula (PASS) of comparing the means of two samples to achieve the power of 90% with $\alpha = 0.05$ (two-sided), provided that the four groups are designed in a 1:1:1:1 ratio. Considering the

dropout rate of 10%, each group is planned to enroll 132 subjects, with a total of 528 subjects.

2.5 Method of Randomization

Using a central randomization method, 528 subjects will be randomized in a 1:1:1:1 ratio to 4 treatment groups: SHR8554 Injection 0.75 mg, SHR8554 Injection 1.0 mg, placebo, and morphine hydrochloride injection.

The random number table will be generated by an unblinded statistician of the statistical team using SAS 9.4, and the blinding will be uniformly completed by an unblinded statistician who does not participate in the study. Randomization will be performed using a central randomization system (Interactive Web Response Randomization System, IWRS). The subjects, investigators, and all personnel involved in the trial or clinical assessment will remain blinded to the true treatment (except the unblinded drug management team) from the randomization until the database is locked.

2.6 Blinded Design

The study will be conducted in a double-blind design and subjects who meet the inclusion criteria will be randomized to each treatment group.

The subjects, investigators, and all personnel involved in the trial or clinical assessment will remain blinded to the true treatment (except the unblinded drug management team) from the randomization until the database is locked.

2.7 Interim Analysis

NA

2.8 Modifications to the Statistical Analysis in the Protocol

NA

3. Efficacy Variables

3.1 Primary Efficacy Variable

- Time-weighted Sum of Pain Intensity Differences at rest up to 24 h after starting infusion of loading dose of investigational product ($SPID_{24}$).

3.2 Secondary Efficacy Variables

- Time-weighted Sum of Pain Intensity Differences at rest at 6 h, 12 h, 18 h, and 12-24 h ($SPID_6$, $SPID_{12}$, $SPID_{18}$, $SPID_{12-24}$);
- Time-weighted Total Pain Relief at 6 h, 12 h, 18 h, 24 h, and 12-24 h ($TOTPAR_6$, $TOTPAR_{12}$, $TOTPAR_{18}$, $TOTPAR_{24}$, $TOTPAR_{12-24}$);
- Time to the first use of rescue analgesics;
- Cumulative amount of rescue analgesics from 0 h to 24 h;
- Number of treatments of rescue analgesia from 0 h to 24 h;
- Total number of presses and number of effective presses of PCA pump from 0 h to 24 h;
- Percentage of subjects who do not use rescue analgesics from 0 h to 24 h;
- The subject's satisfaction score with analgesic treatment;
- The investigator's satisfaction score with analgesic treatment.

4. Safety Variables

4.1 Adverse Events

Treatment-emergent adverse event (TEAE) is defined as any untoward medical condition that occurs after a subject receives the drug, which may manifest as symptoms, signs, diseases, or laboratory abnormalities, but does not necessarily have a causal relationship with the investigational product. AEs include, but are not limited to, the following:

1) Worsening of pre-existing (before enrollment) medical conditions/diseases (including worsening of symptoms, signs, laboratory abnormalities);

2) Any new AEs: any new adverse medical conditions (including symptoms, signs, newly diagnosed diseases);

3) Abnormal and clinically significant laboratory values or results.

The study personnel should record in detail any AE, including: AE name, time of onset,

severity, relationship to the investigational product, end time, action taken with the investigational product, and final results and outcome.

4.2 Treatment-related Adverse Events

The investigator should comprehensively assess and judge the relationship between the AE and the investigational product according to the five-level classification of "related, possibly related, unlikely related, unrelated, not determined". "Related", "possibly related" and "not determined" adverse events are listed as treatment-related adverse events (TRAEs), the sum of which will be the numerator to calculate the incidence of TRAEs, while the number of all subjects for safety evaluation is the denominator.

4.3 Serious Adverse Events

Serious adverse event (SAE) is an event that occurs during the clinical trial that requires hospitalization or prolonged hospitalization, results in disability or incapacity, is life-threatening or fatal, or results in congenital malformations. SAEs include the following medical events:

- Fatal;
- Life-threatening: defined as the subject is at the risk of death at the time of the event rather than assuming a possible death if the condition worsens;
- Requires hospitalization or prolonged hospitalization;
- Leading to permanent or serious disability/incapacity;
- Resulting in congenital anomalies or birth defects;
- Other medically significant events (defined as adverse events that may not immediately result in death, be life-threatening, or require hospitalization, but may jeopardize the subject or require intervention to prevent any of the above outcomes. For example, allergic bronchospasm requiring special treatment in an emergency room or at home, dyscrasia or convulsions that do not result in hospitalization, or development of drug dependence or drug abuse).

4.4 Vital Signs, SpO_2 and Physical Examination

- Vital signs: including blood pressure, respiratory rate, heart rate, and body temperature;
- Oxygen saturation (SpO_2)
- Physical examination: includes overall appearance, skin and mucosa, lymph nodes, head and neck, chest, abdomen, muscles and bones, nervous system, and other parts of the body.

4.5 Laboratory Tests

- Hematology: including red blood cells (RBC), white blood cells (WBC), neutrophils (NEUT), hemoglobin (HB), platelets (PLT);
- Urinalysis: including urine white blood cells (WBC), urine red blood cells (BLD), urine glucose (U-GLU), urine protein (PRO);
- Blood chemistry: including aspartate aminotransferase (AST), alanine aminotransferase (ALT), total bilirubin (TBIL), creatinine (Cr), fasting or non-fasting blood glucose (FBG or BG), urea nitrogen (BUN) or urea (Urea), potassium (K^+), sodium (Na^+);
- Coagulation function: thrombin time (TT), activated partial thromboplastin time (APTT), prothrombin time (PT), fibrinogen (Fib).

4.6 12-Lead ECG

- Includes heart rate, PR, QT, and QTc intervals.

5. Other Variables

NA

6. Analysis Set

The analysis sets include: full analysis set, per protocol set, and safety set.

6.1 Full Analysis Set (FAS)

It includes all subjects who received at least one dose of study drug after randomization in accordance with the intent-to-treat (ITT) principle.

Description of baseline demographic data and efficacy evaluation will be performed in the FAS.

Treatment groups will be based on randomization.

6.2 Per Protocol Set (PPS)

It is a subset of the FAS and includes the subjects who did not have major protocol deviations in the FAS.

Efficacy analysis of this investigational product will be performed in the FAS and PPS.

6.3 Safety Set (SS)

It Includes all enrolled subjects who received the study drug.

This set will be used for safety analysis. Treatment groups will be based on actual grouping.

7. Statistical Analysis

7.1 General Principles

For continuous variables, the number of non-missing subjects, mean, SD, median, minimum and maximum will be listed. The minimum and the maximum values will be rounded to the same decimal place as the original data recorded in the database. The mean and median values will be rounded to 1 more decimal place than the original data recorded in the database, and the standard deviation value will be rounded to 2 more decimal places than the original data recorded in the database, up to a maximum of four decimal places.

Categorical variables will be listed in a frequency table (frequency and percentage). Percentages will be rounded to one decimal place.

7.1.1 Test Level

All hypothesis tests will be performed using a two-side test, with $\alpha = 0.05$; if $P \leq 0.05$, the differences tested will be considered as statistically significant. All confidence intervals

will use 95% confidence.

7.1.2 Hypothesis Testing

The primary efficacy variable of this trial is $SPID_{24}$, and the difference test will be used for the group comparison between the experimental group and the placebo group.

Hypothesis:

H_0: $\mu_T=\mu_C$

H_1: $\mu_T\neq\mu_C$

α level: 0.05 (two-sided).

μ_T: mean $SPID_{24}$ in the experimental group;

μ_C: mean $SPID_{24}$ in the placebo group.

In this study, the high and low dose groups will be tested sequentially to ensure that the Type I error rate α is controlled within 0.05 (two-sided). The test between the high dose group and the placebo group will be performed first with α of 0.05 (two-sided). If there is a significant difference between the high dose group and the placebo group, then the test between the low dose group and the placebo group will be performed with α of 0.05 (two-sided); if there is no significant difference between the high dose group and the placebo group, only exploratory analysis will be performed for the comparison of the low dose group and the placebo group. For the morphine group, only descriptive statistics will be applied.

7.1.3 Estimation of Missing Values

For the pain intensity (PI) score, if the subject does not have the PI score during night sleep, NRS = 3 will be uniformly used for imputation;

For the pain relief (PR) score, if the subject does not have the PR score during night sleep, the previous PR score will be uniformly used for imputation.

Regarding the scores after rescue analgesia, within 6 h after rescue analgesia with parecoxib sodium and within 2 h after rescue analgesia with sufentanil, PI/PR scores at pre-specified time points will be imputed using the PI/PR scores before rescue analgesia.

In other cases, missing PI and PR scores will be imputed using the last observation carried forward (LOCF) method.

Missing values in other secondary efficacy variables and safety evaluations will not be estimated.

7.1.4 Data Conventions

① **Missing dates:** if previous dates are incomplete and affect subsequent date calculations, impute as follows without contradiction to other dates:

- **Dates related to AEs**
  - ✓ Missing day, month and year of AE onset date: the date will not be imputed and is missing;
  - ✓ Missing day and month of AE onset date: if the "year" of the onset date is the same as the "year" of the first dose date, impute as the first dose date; if the "year" of the onset date is early than the "year" of the first dose date, impute 31 December; if the "year" of the onset date is later than the "year" of the first dose date, impute 1 January.
  - ✓ Missing day of AE onset date: if the "month and year" of the onset date are the same as the "month and year" of the first dose date, impute as the first dose date; if the "month and year" of onset date are early than the "month and year" of the first dose date, impute with the last day of the month; if the "month and year" of onset date are later than the "month and year" of the first dose date, impute with 1 as the day.
  - ✓ Missing hour and minute of AE onset date: if the "day, month and year" of onset date are the same as those of the first dose date, impute as the hour and min of the first dose date; if the "day, month and year" of onset date are early than those of the first dose date, impute 23:59; if the "day, month and year" of onset date are later than those of the first dose date, impute 00:00.
  - ✓ Missing minutes of AE onset date: if the "hour and day, month and year" of the onset date are the same as those of the first dose date, impute with the minutes of the first dose date; if the "hour and day, month and year" of onset date are early

than those of the first dose date, impute 59 as the minutes; if the "hour and day, month and year" of onset date are later than those of the first dose date, impute 0 as the minute.

- ✓ Missing day, month and year of AE end date: the end date will not be imputed and will be considered as missing.
- ✓ Missing day and month of AE end date: the end date will be imputed with 31 December.
- ✓ Missing day of AE end date: impute as the last day of the month.

➢ **Dates related to concomitant medications**

- ✓ Missing day, month and year of start date of concomitant medication: the date will not be imputed and is missing;
- ✓ Missing day and month of start date of concomitant medication: if the "year" of the start date is the same as the "year" of the first dose date, impute as the first dose date; if the "year" of the start date is early than the "year" of the first dose date, impute 31 December; if the "year" of the start date is later than the "year" of the first dose date, impute 1 January.
- ✓ Missing day of start date of concomitant medication: if the "month and year" of the start date are the same as the "month and year" of the first dose date, impute as the first dose date; if the "month and year" of start date are early than the "month and year" of the first dose date, impute with the last day of the month; if the "month and year" of start date are later than the "month and year" of the first dose date, impute with 1 as the day.
- ✓ Missing hour and minute of start date of concomitant medication: if the "day, month and year" of start date are the same as those of the first dose date, impute as the hour and min of the first dose date; if the "day, month and year" of start date are early than those of the first dose date, impute 23:59; if the "day, month and year" of start date are later than those of the first dose date, impute 00:00.
- ✓ Missing minutes of start date of concomitant medication: if the "hour and day, month and year" of the start date are the same as those of the first dose date,

impute with the minutes of the first dose date; if the "hour and day, month and year" of start date are early than those of the first dose date, impute 59 as the minutes; if the "hour and day, month and year" of start date are later than those of the first dose date, impute 0 as the minute.

- ✓ Missing day, month and year of end date of concomitant medication: the end date will not be imputed and will be considered as ongoing.
- ✓ Missing day and month of end date of concomitant medication: the end date will be imputed with 31 December.
- ✓ Missing day of end date of concomitant medication: impute as the last day of the month.

②**Baseline** is defined as the last non-missing data before the first dose.

③**Change from baseline** is defined as the test value at follow-up visit after treatment - baseline value.

④**Data derivation and conversion:**

Age: in years, = (date of informed consent - date of birth)/365.25, rounded down.

7.2 Study Population

7.2.1 Case Distribution

Description of subject enrollment, dropouts, and removals from PPS at each site: number and percentage;

Subject disposition for each dataset: subject disposition will be summarized for each analysis population.

Subjects who drop out will be described one by one: medication, reason for dropout, etc.

Subjects who are removed from each analysis population will be listed (including protocol deviations and reasons for exclusion).

7.2.2 Protocol Violations

Protocol violations will be summarized and described.

7.2.3 Analysis of Subject Characteristics

Age: mean, standard deviation, maximum, minimum and median will be used to describe the age;

Sex: frequency and constituent ratio will be calculated;

Ethnicity: frequency and constituent ratio will be calculated;

Height, weight, and body mass index (BMI): mean, standard deviation, maximum, minimum and median will be used;

CYP2D6, CYP3A4 and CYP3A5 genotypes: frequency and constituent ratio will be calculated according to metabolic phenotype;

ASA classification: frequency and constituent ratio will be calculated;

Past medical history and comorbidities, surgical history, smoking, alcohol consumption and allergy history: frequency and constituent ratio will be calculated;

Surgery: frequency and constituent ratio will be calculated according to the method of anesthesia, and χ^2 test will be used for comparison between groups; surgery time will be described using mean, standard deviation, maximum, minimum and median;

Whether sufentanil is given intravenously to prevent breakthrough pain: frequency and constituent ratio will be calculated;

Baseline NRS scores: mean, standard deviation, maximum, minimum and median will be used to describe the scores;

Baseline vital signs (blood pressure, respiratory rate, heart rate, body temperature)/oxygen saturation (SpO_2): mean, standard deviation, maximum, minimum and median will be used;

Baseline physical examination: frequency and constituent ratio will be calculated for the assessment results of each parameter;

Baseline electrocardiogram: heart rate, PR, QT and QTc intervals will be described using mean, standard deviation, maximum, minimum and median; frequency and constituent ratio will be calculated for ECG assessment results;

Infectious disease screening and drug abuse screening: the frequency and constituent

ratio of the assessment results of each indicator will be calculated.

7.3 Efficacy Evaluation

7.3.1 Analysis of Primary Efficacy Variable

- Time-weighted Sum of Pain Intensity Differences at rest up to 24 h after starting infusion of loading dose of investigational product ($SPID_{24}$).

The calculation formula is as follows: $SPID_t$=ΣPIDt*($TIME_t$-$TIME_{t-1}$)(h), e.g. $SPID_2$=(PI_2-PI_0)×(2-1)+(PI_1-PI_0)×(1-0.5)+ ($PI_{0.5}$-PI_0)×(0.5-0).

Note: calculations will be performed using the planned time points.

Mean, standard deviation, maximum, minimum, and median will be used to describe $SPID_{24}$ in each group. Sequential tests will be performed between the groups: the test between the high dose group and the placebo group will be performed first with α of 0.05 (two-sided). If there is a significant difference between the high dose group and the placebo group, then the test between the low dose group and the placebo group will be performed with α of 0.05 (two-sided); if there is no significant difference between the high dose group and the placebo group, only exploratory analysis will be performed for the comparison of the low dose group and the placebo group.

A group t-test will be used for the comparison between two groups, and the difference in $SPID_{24}$ between the two groups and its 95% CI will be calculated.

7.3.2 Analysis of Secondary Efficacy Variables

- Time-weighted Sum of Pain Intensity Differences at rest at 6 h, 12 h, 18 h, and 12-24 h ($SPID_6$, $SPID_{12}$, $SPID_{18}$, $SPID_{12-24}$);

The mean, standard deviation, maximum, minimum and median will be calculated, and sequential tests between groups will be performed, the same as the primary efficacy variable.

- Time-weighted Total Pain Relief at 6 h, 12 h, 18 h, 24 h, and 12-24 h ($TOTPAR_6$, $TOTPAR_{12}$, $TOTPAR_{18}$, $TOTPAR_{24}$, $TOTPAR_{12-24}$);

Calculation formula: TOTPARt=∑[PRt* (TIMEt-TIMEt-1)](h).

The mean, standard deviation, maximum, minimum and median will be calculated, and sequential tests between groups will be performed, the same as the primary efficacy variable.

- Time to the first use of rescue analgesics (h)

Sequential tests will be performed between the groups, and the comparison of differences between the groups will be performed using the log-rank test. For subjects who do not use rescue analgesics, the data will be treated as censored data with censoring time of 24 h.

- Cumulative amount of rescue analgesics from 0 h to 24 h

The mean, standard deviation, maximum, minimum and median will be calculated, and sequential tests between groups will be performed, the same as the primary efficacy variable.

- Number of treatments of rescue analgesia from 0 h to 24 h

The mean, standard deviation, maximum, minimum and median will be calculated, sequential tests between groups will be performed, and Wilcoxon rank sum test will be used.

- Total number of presses and number of effective presses of PCA pump from 0 h to 24 h

The mean, standard deviation, maximum, minimum and median will be calculated, sequential tests between groups will be performed, and Wilcoxon rank sum test will be used.

- Percentage of subjects who do not use rescue analgesics from 0 h to 24 h

The proportion of subjects who do not use rescue analgesics and its 95% CI will be calculated in each group. The 95% CI will be calculated by Clopper-Pearson method. Sequential tests will be performed between the groups and the rate difference between the two groups and its 95% CI will be calculated.

- The subject's satisfaction score with analgesic treatment

The mean, standard deviation, maximum, minimum and median will be calculated, sequential tests between groups will be performed, and Wilcoxon rank sum test will be

used.

- The investigator's satisfaction score with analgesic treatment

  The mean, standard deviation, maximum, minimum and median will be calculated, sequential tests between groups will be performed, and Wilcoxon rank sum test will be used.

7.4 Safety Evaluation

7.4.1 Drug Exposure

The subject's drug exposure in each group will be described as mean, standard deviation, maximum, minimum and median.

7.4.2 Adverse Events

TEAEs will be coded according to the current version of MedDRA at the time of coding and processed in the statistical analysis.

- The number and incidence of treatment-emergent adverse events/treatment-related adverse events, treatment-emergent serious adverse events/treatment-related serious adverse events, and adverse events leading to withdrawal from the study/treatment-related adverse events will be summarized by SOC and PT;
- Treatment-emergent adverse events/treatment-related adverse events occurring in ≥ 10%/≥ 5% of subjects (≥ 10%/≥ 5% in any group) will be summarized by PT;
- The number and incidence of adverse events of special interest, i.e., nausea, vomiting, dizziness, respiratory depression (respiratory depression defined as respiratory rate < 8 beats/min and/or oxygen saturation < 90%) during the treatment period, will be summarized;

If a subject experiences multiple AEs, he/she will be counted only once in incidence calculation; if a subject experiences the same AE for multiple times, he/she will be counted only once in the incidence calculation of this AE.

AEs will be summarized in frequency tables by system organ class (SOC) and preferred term (PT). Incidence (number of subjects: number of subjects with at least one AE) will

be calculated by system and symptom/sign.

The number and incidence will be calculated by severity and relationship to study drug, SOC and PT, respectively (for AEs with repeated occurrences, the event with the worst severity will be calculated for analysis).

A listing of all AEs and SAEs experienced by the subjects will be provided.

7.4.3 Vital Signs

Mean ± standard deviation, maximum, minimum, and median will be used to describe the measurements and changes before and after treatment.

A listing of vital signs by subject for all visits will be provided.

7.4.4 Laboratory Measurements

Mean ± standard deviation, maximum, minimum and median will be used to describe the measurements and changes of hematology, blood chemistry and coagulation function before and after treatment. Crossover classification table will be used to describe the normal and abnormal changes before and after treatment.

Urinalysis: crossover classification table will be used to describe the normal and abnormal changes before and after treatment.

The proportion of subjects with "abnormal and clinically significant" changes will be described among subjects with abnormal changes, in which the clinical significance of the abnormality will be determined by the investigator. A listing of abnormalities after treatment will be provided.

Listings of hematology, urinalysis, blood biochemistry and coagulation function data by subject at all visits will be provided.

7.4.5 Electrocardiogram

Mean ± standard deviation, maximum, minimum and median will be used to describe the measurements and changes of heart rate, PR, QT and QTc intervals before and after treatment.

Crossover classification table will be used to describe the normal and abnormal changes before and after treatment. The proportion of subjects with "abnormal and clinically significant" changes will be described among subjects with abnormal changes, in which the clinical significance of the abnormality will be determined by the investigator. A listing of abnormalities after treatment will be provided.

A listing of 12-lead ECG data by subject at all visits will be provided.

7.4.6 Physical Examination

Crossover classification table will be used to describe the normal and abnormal changes before and after treatment. The proportion of subjects with "abnormal and clinically significant" changes will be described among subjects with abnormal changes, in which the clinical significance of the abnormality will be determined by the investigator. A listing of abnormalities after treatment will be provided.

A listing of physical examination data by subject for all visits will be provided.

7.4.7 Transdermal Oxygen Saturation (SpO_2)

Mean ± standard deviation, maximum, minimum, and median will be used to describe the measurements and changes before and after treatment.

A listing of SpO_2 data by subject for all visits will be provided.

7.4.8 Concomitant Medications and Concomitant Non-drug Therapies

The use of concomitant medications during the study and the frequency of use of each drug will be summarized. Concomitant medications will be coded by ATC classification, and frequencies will be summarized and described by Anatomical Classification (ATC Level 1 coding) and Therapeutic Classification (ATC Level 2 coding).

Concomitant non-drug therapies during treatment: the frequency and constituent ratio will be calculated.

A listing of concomitant medications and concomitant non-drug therapies used by

subjects will be provided.

7.5 Sensitivity Analysis

- Time-weighted Sum of Pain Intensity Differences at rest up to 24 h after starting infusion of loading dose of investigational product ($SPID_{24}$). (Score not imputed after rescue analgesia)
- Time-weighted Sum of Pain Intensity Differences at rest up to 24 h after starting infusion of loading dose of investigational product ($SPID_{24}$). (Randomized population)

7.6 Subgroup Analysis

NA

7.7 Other Analysis

NA

8. Statistical Analysis Software

The statistical analysis will be performed using SAS 9.4.

9. References

- China Food and Drug Administration. *Drug Registration Regulation*. July 2020.
- China Food and Drug Administration. *Good Clinical Practice*. July 2020.
- International Conference on Harmonisation of Technical Requirements for Registration of Pharmaceuticals for Human Use. *E9 Statistical Principles for Clinical Trials*.
- China Food and Drug Administration. *Biostatistics Guidelines for Drug Trials*. June 2016
- China Food and Drug Administration. *Guidelines for Planning and Reporting of Clinical Trial Data Management and Statistical Analysis*. July 2016
- International Conference on Harmonisation of Technical Requirements for Registration of Pharmaceuticals for Human Use. *E3 Structure and Content of Clinical Study Reports*.

10. Appendixes
